# Post-Assay Chemical Enhancement for Highly Sensitive Lateral Flow Immunoassays: A Critical Review

**DOI:** 10.3390/bios13090866

**Published:** 2023-09-01

**Authors:** Vasily G. Panferov, Anatoly V. Zherdev, Boris B. Dzantiev

**Affiliations:** 1 A.N. Bach Institute of Biochemistry, Research Center of Biotechnology of the Russian Academy of Sciences, 119071 Moscow, Russia; vaspanferov@gmail.com (V.G.P.); zherdev@inbi.ras.ru (A.V.Z.); 2 Department of Chemistry, York University, Toronto, ON M3J 1P3, Canada

**Keywords:** immunochromatography, highly sensitive detection, signal amplification, nanoparticles, nanozymes, point-of-care testing, antibodies

## Abstract

Lateral flow immunoassay (LFIA) has found a broad application for testing in point-of-care (POC) settings. LFIA is performed using test strips—fully integrated multimembrane assemblies containing all reagents for assay performance. Migration of liquid sample along the test strip initiates the formation of labeled immunocomplexes, which are detected visually or instrumentally. The tradeoff of LFIA’s rapidity and user-friendliness is its relatively low sensitivity (high limit of detection), which restricts its applicability for detecting low-abundant targets. An increase in LFIA’s sensitivity has attracted many efforts and is often considered one of the primary directions in developing immunochemical POC assays. Post-assay enhancements based on chemical reactions facilitate high sensitivity. In this critical review, we explain the performance of post-assay chemical enhancements, discuss their advantages, limitations, compared limit of detection (LOD) improvements, and required time for the enhancement procedures. We raise concerns about the performance of enhanced LFIA and discuss the bottlenecks in the existing experiments. Finally, we suggest the experimental workflow for step-by-step development and validation of enhanced LFIA. This review summarizes the state-of-art of LFIA with chemical enhancement, offers ways to overcome existing limitations, and discusses future outlooks for highly sensitive testing in POC conditions.

## 1. Introduction

**Figure 1:**
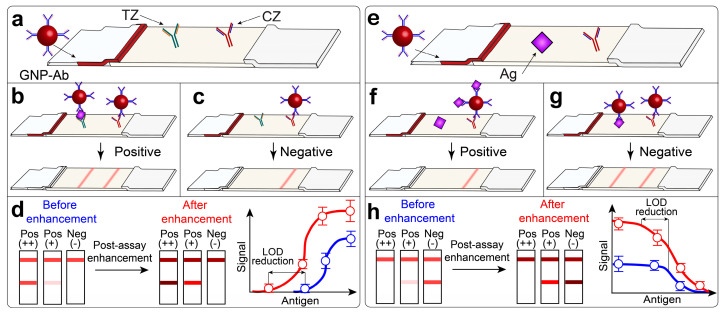
The principle of LFIA in the sandwich (**a**–**d**) and competitive (**e**–**h**) formats. (**a**) Structure of the test strip for LFIA in the sandwich format. GNP-Ab stands for the conjugate of gold nanoparticles (GNP) with antibodies. TZ stands for test zone. TZ contains antibodies against antigen. CZ stands for control zone. CZ contains binders of GNP-Ab. (**b**) LFIA for samples with antigen. (**c**) LFIA for samples without antigen. The upper parts of (**b**,**c**) show the structure of immunocomplexes in TZ and CZ after completion of LFIA. The bottom parts show the appearance of test strips after completion of LFIA. (**d**) Post-assay enhancement of sandwich LFIA. The appearance of test strips before and after enhancement and calibration plots before (blue) and after (red) enhancement are schematically shown. (**e**) Structure of the test strip for LFIA in the competitive format. TZ contains immobilized antigen (Ag). (**f**) LFIA for samples with antigen. (**g**) LFIA for samples without antigen. The upper parts of (**f**,**g**) show the structure of immunocomplexes in TZ and CZ after completion of LFIA. The bottom parts show the appearance of test strips after completion of LFIA. (**h**) Post-assay enhancement of competitive LFIA. Schematically shown are the appearance of test strips before and after enhancement, and calibration plots before (blue) and after (red) enhancement.

Lateral flow immunoassay (LFIA) is an analytical method that combines highly specific antibody–antigen interaction and the affine partitioning of target and non-target species during migration through a porous nitrocellulose membrane. LFIA is performed using multimembrane assemblies (test strips) containing all reagents predispersed (Figure 1). Usually, test strips have two zones (test zone and control zone, TZ and CZ, respectively) with immobilized immunoreagents. Liquid sample migrates along the test strip by capillary forces, rehydrates reagents, and initiates the formation of immunocomplexes [1]. For the detection of immunocomplexes, various nanosized labels are used [2,3]. Labeled immunocomplexes are usually detected visually (by the coloration of specific zones on the test strip), although alternative registration methods are being actively investigated [4,5].

LFIA can be performed in two main formats [6]. LFIA in a sandwich format (Figure 1a) is used for the detection of high-molecular (proteins, polysaccharides) or corpuscular (cells, viral particles) antigens that have multiple sites for antibody binding. For sandwich LFIA, the intensity of TZ coloration is directly related to the concentration of antigen in the sample (Figure 1b,d). The absence of TZ coloration is interpreted as the absence of the antigen in the sample (Figure 1c). LFIA in a competitive format (Figure 1e) is used for the detection of small molecules (antibiotics, pesticides, mycotoxins, drugs, etc.) that have one site to bind antibodies. For competitive LFIA, the intensity of TZ coloration is reversibly related to the concentration of antigen in the sample (Figure 1f,h). The coloration of the control zone is considered an internal positive control for each test strip and is a mandatory requirement for the validity of results in both formats (Figure 1c,g).

LFIAs find wide applications in clinical diagnostics, veterinary, food control, and environmental monitoring [7,8,9]. Such widespread use is explained by simple sample preparation, assay performance, the registration of the results, rapidity (less than 10–15 min), and low cost [7]. Because of these benefits, many LFIAs have been developed to the level of commercially available test systems (including well-known rapid tests for the detection of pregnancy, drugs, and SARS-CoV-2). However, the set of analytes for commercialized LFIAs is significantly smaller than those reported in scientific articles. One of the major reasons for this shortage is an insufficient-low limit of detection (LOD) of LFIA [7,10]. Limitations of LFIA’s applicability are especially pronounced in medical diagnostics [7]. Many disease biomarkers are presented in concentrations below the typical sub-ng/mL LOD of LFIA [11]. Thus, more sensitive methods (ELISA, PCR, cultivation on a growth media, etc.) are used for these compounds in clinical practice. However, the use of these assays is limited to equipped laboratories and trained technicians. The endeavors to reduce the LOD of LFIA should not violate the benefits of the method (i.e., user-friendliness, rapidity, and low cost). Reducing the LOD of LFIA is the priority direction in developing immunochemical point-of-care assays [5] along with multiplexing [12].

### Factors Determining the LOD of LFIA

The sensitivity of LFIA is determined by the number of parameters at all stages, from sample preparation and the selection of antibodies to registration and processing of the results [13]. However, the optimization of sample preparation, choice of antibodies, and LFIA’s conditions (type of membrane, concentration of immunoreagents) can be performed in a limited range. Available manipulations during sample preparation are restricted by the physicochemical properties of the target molecule (i.e., molecular weight, solubility in water and organic solvents, charge, etc.) and usually should be selected for each target individually. The choice of antibodies is often determined by their availability. Fundamentally, antibodies with pico-molar and lower affinity are relatively rare because of the nature of immunoresponse. Accepting that the association rate of antibody–antigen interaction is limited by the diffusion rate (*k_a_* ≈ 10^6^ M^−1^ s^−1^). The affinity is limited by the dissociation rate constant *k_d_* ≈ 10^−4^ s^−1^, which can be explained by the lack of evolutionary necessity for further reducing *k_d_* during immunoresponse [14,15]. Thus, the selection of antibodies for LFIA remains an empirical task. Varying of the interaction conditions (i.e., the creation of more favorable conditions for antibody–antigen binding) is limited to increasing the interaction time by preincubation before LFIA [16] and migration time on the test strip by changing its geometry [17].

Thus, the explicit focus on post-assay enhancement as a universal strategy seems reasonable from a practical view. Post-assay enhancement approaches based on chemical reactions are an actively developing area in LFIA [7,18]. Many existing enhancement methods are performed in a post-assay manner—e.g., registration of surface-enhanced Raman spectroscopy [19], fluorescent [20], magnetic [21], and thermo [22] signal readout. However, all these methods require additional costly equipment and have limited applicability in POC conditions. On the contrary, post-assay enhancement approaches based on chemical reactions are easy to use and can be performed by the analyst without training. These chemical reactions occur at room temperature, do not require highly toxic compounds, and facilitate a significant decrease in LOD.

Before considering post-assay enhancement approaches, one needs to establish the parameters determining the value of the registered signal in the test zone. As most scientific developments and commercial products utilize GNPs as labels, we discuss the factors influencing the LOD value on the example of GNPs as the colorimetric label. For post-assay enhancement, we will consider the test strip after sandwich LFIA as a model. The color intensity of the TZ is directly related to the concentration of labeled immunocomplexes. Khlebtsov and coauthors [23] have experimentally determined the surface density of GNPs (i.e., the number of spherical GNPs with diameters from 16 to 115 nm per mm^2^) on the membrane sufficient for the formation of the detectable colored zone. The authors experimentally showed that the number of GNPs sufficient for the coloration corresponding to visual LOD is irreversibly proportional to *d*^3.1^, where d is the diameter of the GNP. In accordance with their results, GNPs with a diameter of 16 nm require 6.5 × 10^7^ particles/mm^2^, while 115 nm GNPs require only 1.4 × 10^5^ particles/mm^2^. It is important to note that these calculations were made for GNPs being passively adsorbed on the membranes. Thus, these estimations cannot be directly transferred for quantitative characterizations of LFIAs. First, larger particles have poor colloidal stability and migration through the membranes. Thus, the larger particles will bind to the membrane non-specifically. As a result, higher background, limiting the assay sensitivity, will be observed. Second, a steric hindrance for immunobinding for larger particles may result in higher LOD values. However, considering post-assay signal amplification, one may neglect affine binding and poor colloidal stability of larger particles, as the signal amplification is performed after completing LFIA. Thus, following the results of Khlebtsov and coauthors [23], to increase the coloration of the test zone (and hence the sensitivity of the assay) after the performance of conventional LFIA, one needs to increase the size and number of GNPs.

Many efforts have been made to increase the sensitivity of LFIA, which are summarized in multiple reviews [3,7,18,24,25]. However, most reviews examine all the existing approaches for LOD reduction, from selecting high-affine binders to instrumental methods of signal registration. Thus, post-assay chemical enhancement approaches are discussed briefly, without critically evaluating the benefits and drawbacks of their variants. Although such reports are undoubtedly important, the applicability and possible LOD decrease rates of the approaches remain undiscussed. In this review, we focus only on post-assay enhancement approaches with chemical reactions for sensitivity enhancement.

In the second section, we suggest a classification of the post-assay enhancement approaches based on the signal amplification mechanism. Then we briefly discuss each approach with references to the main articles published in the area. In the third section, we discuss the developments and state-of-the-art of LFIA with an integrated signal amplification step. In the fourth section, we quantitatively compare the enhancement approaches. In addition, we report and explain discrepancies in the literature focusing on post-assay enhancement of LFIA. In the fifth section, we suggest a workflow for the development and validation of LFIA with post-assay enhancement. We believe this review will be interesting for the researchers developing LFIAs and practitioners in the industry working on commercializing such rapid tests.

## 2. Post-Assay Chemical Enhancement Approaches

Post-assay chemical enhancement approaches are focused on the sensitivity enhancement of LFIA by amplifying the registered signal from immunocomplexes in TZ. The signal is amplified by the performance of various chemical reactions.

We determine four criteria to distinguish the post-assay chemical enhancement methods from all others. Post-assay chemical enhancement methods (a) are performed after completion of conventional LFIA in situ (i.e., after the formation of immunocomplexes on the test strip); (b) are aimed at the amplification/generation of the signal from the immunocomplexes; (c) utilize chemical reactions for the amplification/generation of the signal; and (d) are performed on demand (i.e., if the coloration of the conventional LFIA is strong enough, the user can decide not to perform the additional enhancement step).

As indicated above, in the post-assay conditions, the coloration of the TZ is determined by the size and concentration of the nanosized label [23]. Thus, post-assay approaches for sensitivity enhancement can be ultimately categorized into two groups:

1. Approaches focused on the modification of physicochemical properties of the nanosized labels. The ultimate goal is increasing the “visibility” of nanoparticles on the membrane. Among these properties are size, shape, and chemical composition affecting the optical properties of nanoparticles [26,27]. Further in this paper, we focus on GNPs as the most widely used nanolabel in LFIA. The intensity of the coloration of GNPs on the membrane is determined by light absorption and scattering, and the impact of each parameter is determined by the size of the particles [23]. Larger GNPs have a higher scattering intensity and molar extinction coefficient, which provides higher coloration for the given number of particles [28,29,30]. Non-spherical GNPs also have a higher extinction coefficient than similarly sized spherical GNPs [31]. Changing the chemical composition of nanoparticles by in situ formation of metal shells (Cu, Ag, Pt) over initial GNPs also increases the molar extinction coefficient and facilitates highly sensitive detection [32]. The signal amplification is based on in situ formation of particles that can be detected at lower concentrations than initial GNPs.
2. Approaches focused on the increase of the label number are aimed at the accumulation of an additional amount of labels (driven by non-covalent binding between labels) or by catalytic conversion of a substrate to the detectable product (usually its oxidation to a colored or fluorescent product) [25]. The signal amplification is based on increasing the concentration of the registered product of the catalytic reaction.

Further in this paper, we review the methods of signal enhancement in both groups. We discuss the mechanism of signal amplification, the performance of enhancement, its advantages, and possible pitfalls. The quantitative characteristics of enhancement are discussed in Section 4 of this review.

### 2.1. Modification of Physicochemical Properties of Nanoparticles

**Figure 2:**
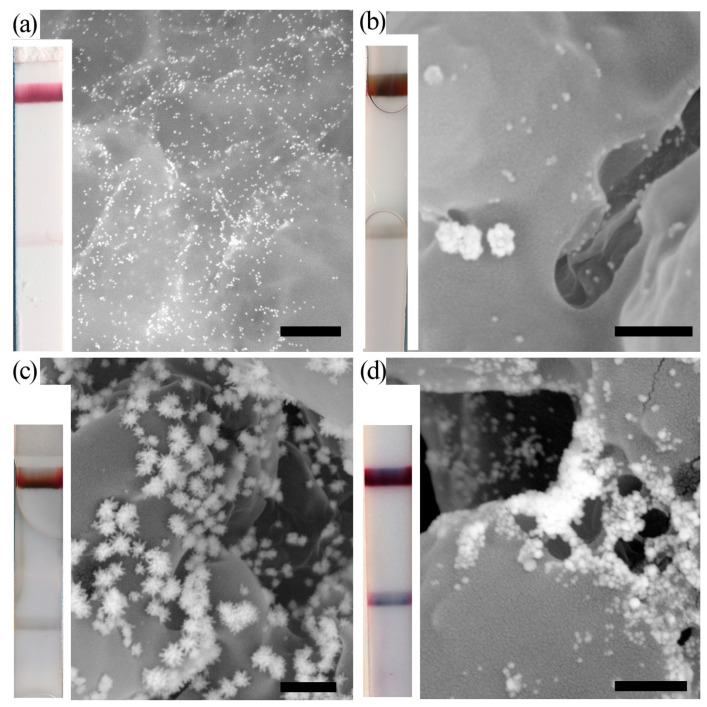
SEM microphotographs of test zones and corresponding test strips. (**a**) GNPs before enhancement. (**b**) Au@Ag nanoparticles after silver enhancement. (**c**) Au@Ag-Au nanoparticles after silver enhancement and galvanic-assisted Pt deposition. (**d**) Enlarged GNP after gold enhancement. The bars are equal to 500 nm [40].

These approaches include several chemical reactions that change the physiochemical properties of the nanosized label. Such reactions involve gold, silver, and copper enhancement. The principle of all these metal enhancements is similar. GNPs act as the catalyst in the reaction of metal salt reduction. As a result, core@shell nanoparticles (GNP as a core, reduced metal as a shell) are formed in situ (Figure 2a,b,d). Such newly formed core@shell nanoparticles are larger than the initial GNP and facilitate a higher colorimetric signal. These nanoparticles can be detected in lower concentrations, thus providing detectable coloration for the initially low/undetectable concentrations of GNPs.

Chemical reactions of metal reduction with GNPs in the colloidal solution and on the membrane (surface-bounded) have different kinetics and facilitate the formation of particles of varying morphology [33,34,35]. Bare GNPs in the solution form monodisperse core@shell nanoparticles [36], while larger and non-spherical particles are formed for immobilized GNPs [37,38]. This observation can be explained by the non-uniform diffusion of the enhancing reagents to the surface of immobilized GNPs. Using GNPs of various sizes immobilized on silica oxide, Festag and coauthors [39] showed that the size and morphology of the nanoparticles after silver and gold enhancement depends on the density of the particles, the size and charge of the particles, and the diffusion of enhancing reagents to the particles. The areas with a higher density of immobilized particles show lower enlargement of particles due to limited diffusion of enhancing reagents. This observation explains lower signal amplification for high-colored zones (high antigen concentration) than for low-colored zones (low antigen concentration). Such discrepancy in signal amplification between initially high and low colored zones does not hinder the application of enhancement, as the users are interested in amplification of low/non-detectable signals. The initial larger particles tend to form larger particles after enhancement compared with smaller ones. Also, the authors experimentally demonstrated that the stepwise addition of enhancing reagents facilitates the formation of almost three-times-larger GNPs. This observation was explained by the localized depletion of enhancing reagents. As the developers of enhanced LFIA aim for maximum signal enhancement, they must consider the possible impact of reagent depletion (especially while enhancing the areas with a high density of immobilized GNPs).

Important to note is that GNPs are not unique catalysts for the reactions of gold and silver enhancement. Wang and coauthors [41] performed silver enhancement using quantum dots as a catalyst. Dias and coauthors [42] reported gold enhancement catalyzed by silver, iron oxide, and silica nanoparticles.

In addition, methods of changing the chemical composition of nanoparticles via galvanic reaction were recently developed for LFIA enhancement [40,43]. The method is based on the electrochemical process leading to the oxidation of one metal (particles) by the ions of another metal (in solution). Metal in the particles will be oxidized and dissolved while the ions of the second metal will be deposited on the particle [44]. As a result, particles of different chemical compositions and morphologies (Figure 2c) are synthesized.

#### 2.1.1. Gold Enhancement

Gold enhancement is based on HAuCl_4_ reduction by various reducing agents (hydrogen peroxide [42], hydroxylamine [36], MES [45], and cethyltriammonium bromide-ascorbic acid [46]) in the presence of GNPs. Hydroxylamine and hydrogen peroxide are the most commonly used reducing agents for LFIA. Gold enhancement was initially developed to increase small GNPs’ visibility in immunohistochemistry [47]. Since then, gold enhancement has been widely utilized in various bioanalytical techniques [48,49,50]. Focusing on LFIA, gold enhancement is used to increase the detectability of GNP in the test zone by increasing their size.

The chemical equation of gold enhancement (for the case of hydroxylamine as the reducing agent) is the following (Equation (1)) [51].

4HAuCl_4_ + 6NH_2_OH = 4Au + 3N_2_O +3H_2_O + 16HCl (1)

As a result of this reaction, gold atoms are reduced on the surface of GNPs, forming enlarged particles. GNPs act as a catalyst in the reduction reaction of gold salt. Some articles report that the reduced gold atoms form new layers around existing GNPs [36,52,53]. Thus, no GNPs should be formed de novo in the enhancing solution (mix of HAuCl_4_ and reducing agent). However, some articles report in situ formation of GNPs in enhancing solution without adding seeds of GNPs [54,55]. It is crucial to suppress in situ formation of GNPs in the enhancing solution, as it causes non-specific background limiting LOD reduction [51,56]. Components of gold enhancing solution can be stored separately for a long time and mixed right before the enhancement. Considering all published protocols and the available commercial products (e.g., multicomponent “Goldenhance“ from Nanoprobes) to date, the long-term storage of the premixed gold enhancement is not recommended, as it may cause high background and low signal amplification due to the self-nucleation of GNPs. Thus, the components for gold enhancement should be stored separately and premixed only before use.

Gold enhancement is actively used for the post-assay signal enhancement of various analytes, e.g., viruses [57,58,59], pathogenic bacteria cells [60,61,62], high molecular weight [51,63], and small molecular weight biomarkers [64]. Duan and coauthors [51] demonstrated that gold self-nucleation in the enhancing solution (hydroxylamine was used as the reducing agent) was significantly more pronounced at higher pH and directly related to hydroxylamine concentrations.

**Figure 3:**
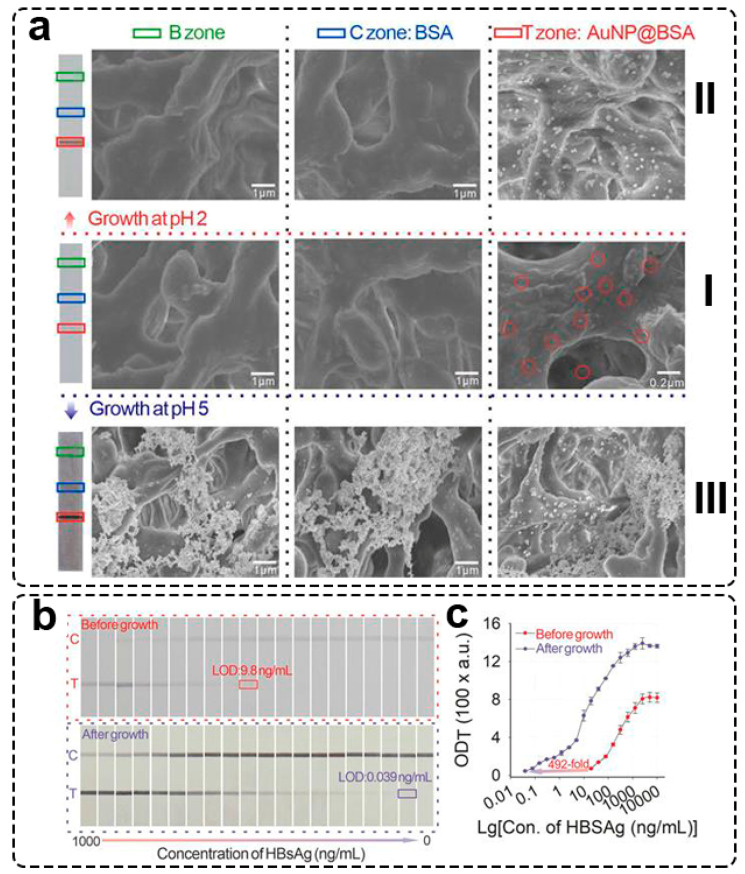
Gold enhancement for LFIA amplification. (**a**) Effect of pH on gold enhancement. Scanning electron microscopy was performed on three zones of test strips. B zone—blank membrane containing neither GNPs nor immobilized proteins. C zone contained 1 mg/mL of BSA. T zone contained 20 pM/L of BSA-GNP conjugate. I—test strip and microphotographs of three zones before gold enhancement. Red circles show the localization of GNP in T zone. II—test strip and microphotograph of three zones after gold enhancement at pH = 2. III—test strip and microphotograph of three zones after gold enhancement at pH = 5. (**b**) LFIA of hepatitis B surface antigen (HBsAg), test strips before and after gold enhancement. (**c**) Calibration plots for LFIAs before and after gold enhancement [51].

The deprotonated form (NH_2_OH) of hydroxylamine at high pH has a higher reducing ability than its protonated form (NH_3_OH) at lower pH. Duan and coauthors [51] studied the effect of pH on the gold enhancement of GNPs deposited on the membrane. Three zones on the test strip were studied (Figure 3a(I))—the blank zone (bare nitrocellulose membrane), control zone (BSA was immobilized), and test zone (conjugate of GNP with BSA was immobilized, shown with red circles, Figure 3a(I)). Gold enhancement was performed at pH = 2 and pH = 5. The authors did not observe gold nucleation in the blank (B zone, Figure 3a(II) and control (C zone, Figure 3a(II)) zones when pH = 2. Enlarged GNPs were detected only in the test zone (T zone, Figure 3a(II)). These results confirm that selected conditions for gold enhancement facilitate low background coloration. On the contrary, non-specific nucleation of GNPs in the blank zone and control zones was observed at pH = 5 (Figure 3a(III)). Such non-specific nucleation facilitates higher background staining and is not applicable for LFIA enhancement. To confirm their statement about the effect of the reducing ability of HA on the performance of gold enhancement, Duan and coauthors [51] performed gold enhancement using chemical derivatives of HA –N-tert-butylhydroxylamine (containing an electron-donating group, i.e., having a higher reducing ability than hydroxylamine) and N-hydroxyacetamine (containing an electron-withdrawing group, i.e., having a lower reducing ability than hydroxylamine). As expected, the reducing agent with the electron-donating group showed a significantly higher rate of Au^3+^ consumption with and without the addition of seed GNPs. These results prove that hydroxylamine (and analogs) reducibility is determined by the electron density of the hydroxyl group, which depends on the aminogroup’s protonation.

The formation of HCl during gold enhancement (Equation (1)) decreases the pH value and slows down the reaction due to the decline of the reducing ability of HA. However, even at low pH values, reduction of gold will occur, so the reaction (1) in minute timescale is limited by the concentration of gold salt [36]. Important to note is that pH also affects the reactivity of gold precursor [56]. Based on the pH of the solution, the various forms of ions will be in equilibrium, and the highest reactivity will be observed at low pH values [65].

For the biological samples, there are no reported inhibitors of gold enhancement. On the one hand, this is beneficial for the users, as the performance of gold enhancement does not require any additional sample preparation or test strip cleaning procedures. On the other hand, it means that there are no methods for terminating gold enhancement. Thus, washing test strips from enhancing reagents is the only option to terminate gold enhancement in a timely manner. If the gold enhancement is not terminated, a high background will be observed, hindering the visual detection of the results.

#### 2.1.2. Silver Enhancement

Silver enhancement is based on the reduction of silver salts (silver nitrate, lactate, or acetate) by the reducing agent in the presence of GNPs. Metol, ascorbic acid, ammonium iron (II) sulfate, pyrogallol, formalin, and hydroquinone were reported as the reducing agents [66,67,68]. Hydroquinone is the most commonly used reducing agent for silver enhancement that is also widely used in photography. Silver enhancement has been known for a while, being actively used to enlarge GNPs in immunohistochemistry [69,70]. Enlarged core@shell Au@Ag nanoparticles can be easily localized in tissues. GNPs are considered the catalyst in the reduction reaction of Ag^+^ ions. However, early reports show that insoluble metal sulfides can also catalyze the reaction of silver reduction [66,71].

Among three metal enhancement approaches, silver enhancement utilizes less stable reagents. Even exposure to light can catalyze the reduction of silver ions in the solution. Hydroquinone also tends to oxidize during storage with the formation of 1,4-benzoquinone. As a result of low stability, low LOD decrease and/or high background will be observed. Even thorough optimization could not completely suppress the background, as silver ions interact with proteins immobilized on the membranes [72]. These bounded ions will be reduced with the formation of Ag particles, resulting in the grey coloration of the test strip. Many efforts focused on developing more stable silver-enhancing solutions [67,73]. Among the practical recommendations (separate storage of silver salt and reducing agent in the darkened place), modified protocols were proposed [74]. As the protocols were optimized for microscopy applications, the efforts were focused on preserving the monodispersity and spherical shape of enlarged particles by slowing down the silver reduction reaction [69].

The source of silver ions is an important parameter affecting enhancement efficiency and background staining. Silver lactate and acetate are partly ionized in the solution, while silver nitrate is whole ionized salt. The high concentration of Ag^+^ ions in the solution can result in a faster reduction rate, leading to background staining [70,71]. Silver acetate is also considered “light-insensitive” [75], which makes this precursor applicable to POC settings [76].

The presence of high-molecular polymer for the stabilization of particles facilitates low background [77]. Scopsi and coauthors [70] demonstrated that Au@Ag nanoparticles enlarged in the presence of gum arabic preserve the initial spherical shape. However, such particles were smaller compared with those synthesized without polymer. Also, the protocol is time-consuming and requires around 60 min for enhancement. Using PEG and PVP as the stabilizing polymers, more rapid silver enhancement (around 10 min) was achieved, and the particles were larger and non-spherical. Silver enhancement without any protective polymer resulted in the formation of irregular silver precipitate and high background even after 5 min.

**Figure 4:**
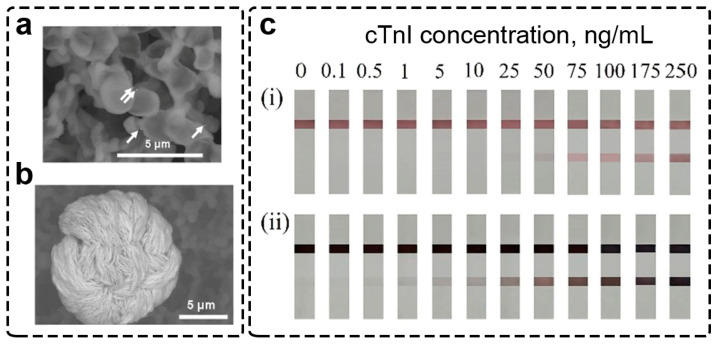
Silver enhancement for LFIA amplification. (**a**) Microphotograph of nanoparticles on the test strip before silver enhancement. The arrows show the position of nanoparticles. (**b**) Microphotograph of nanoparticles after silver enhancement [68]. (**c**) Use of silver enhancement for LFIA of cardiac troponin I. Concentrations of cardiac troponin I are shown above the test strips (i) before silver enhancement and (ii) after silver enhancement [87].

Interesting to note is that these improvements were reported for microscopy. For signal amplification in LFIA, “not optimal” protocols are used. There are no articles using polymer-stabilized silver enhancement components, and no direct comparison of silver salts on the enhancement performance was reported for LFIA. However, the optimization of this protocol was performed for microscopy usage; the silver enhancement reaction was slowed down to maintain the spherical shape of nanoparticles. For LFIA, there is no need to keep the particles spherical after enhancement. On the contrary, large and non-spherical particles (Figure 4a,b) are reported as the more efficient colorimetric labels. Enlarged Au@Ag nanoparticles have black/dark grey coloration and can be easily detected on the white background of the nitrocellulose membrane (Figure 4c). Silver enhancement is actively used for the post-assay signal enhancement of various analytes, e.g., viruses [78,79], pathogenic bacteria cells [80,81], xenobiotics in food [82,83,84], and high molecular [85] and small molecular weight biomarkers [86].

On the one hand, the low stability of silver enhancement components is a drawback for POC application. On the other hand, it provides a tool for the termination of enhancement reaction on the test strip. In one of the first articles showing silver enhancement in LFIA, the reaction was terminated by adding sodium thiosulfate solution (Equation (2)) [88].

2AgNO_3_ + Na_2_S_2_O_3_ → Ag_2_S_2_O_3_ + 2NaNO_3_ (2)

The formation of low-soluble silver thiosulfate terminated the reaction of silver enhancement due to the reduction in Ag^+^ concentration. Similarly, Panferov and coauthors used Cl^-^-containing buffer for termination silver enhancement (Equation (3)) [78].

AgNO_3_ + NaCl→ AgCl + NaNO_3_ (3)

Among all post-assay enhancements, LFIA with silver enhancement was developed to the stage of commercial products. The Fujifilm SILVAMP TB LAM assay was reported for the detection of lipoarabinomannan—a heat-stable secreted component of the outer cell wall of *Mycobacterium*—in urine [89]. The device contains two buttons that release silver-enhancing reagents to the test strip after the completion of the assay. Such LFIA with silver enhancement facilitates high clinical sensitivity and is applicable for POC diagnosis of tuberculosis. A similar principle was realized in the test system FUJI DRI-CHEM IMMUNO AG Cartridge FluAB for the simultaneous detection of influenza A and B viruses [79] and SARS-CoV-2 nucleocapsid protein [90]. Couturier and coauthors showed high reproducibility of the silver-enhanced LFIA during storage for seventeen weeks at temperatures 4–37 °C, confirming the high stability of the enhancing solutions developed by Fujifilm [91].

#### 2.1.3. Copper Enhancement

Copper enhancement is based on the reduction of copper salts (usually CuSO_4_ and CuCl_2_) by the reducing agent in the presence of GNPs. Sodium ascorbate is the most widely used reducing agent, while some other compounds (sodium borohydride, sodium citrate, and ascorbic acid) have been tested [92]. GNPs are reported to be a catalyst in the reduction of copper salt. As shown by Wei and coauthors [93], the reduction of copper sulfate by ascorbic acid can be catalyzed by Au, Pt, Pd, Ag, Fe_3_O_4_, and Cu_2_O nanoparticles as well. First, Cu^2+^ is reduced by ascorbic acid to Cu^+^ that adsorbs on the surface of nanoparticles, and can be further reduced by ascorbic acid to Cu^0^, forming core@shell nanoparticles. While GNPs showed higher activity, one needs to understand that many nanoparticles can catalyze the copper reduction reaction.

Copper enhancement can be performed with or without the addition of stabilizing polymers in the enhancing solution. Kim and coauthors [94] used a mixture of CuCl_2_ and polyethyleneimine (PEI). Amino groups of PEI bind copper ions, reducing the concentration of free ions in the solution. As a result, the self-nucleation reaction is inhibited, facilitating lower background coloration [95]. The same mechanism was reported for polyallylamine hydrochloride, another amine-containing polymer [96]. PEI acts as a capping agent, facilitating the formation of polyhedral core@shell Au@Cu nanoparticles. Multiple corners and edges, typical for polyhedral structures, increase the scattering properties of nanoparticles. PEI-assisted copper enhancement was used for colorimetric signal enhancement in dot blot assay of *Mycobacterium tuberculosis* antigens CFP-10 [92] and 85B [97] and bovine viral diarrhea virus [98]. Some articles [95,98] report two-step enhancement protocols. In the first step, PEI-Cu^2+^ complexes bind to GNPs. In the second step, sodium ascorbate is added to reduce adsorbed on GNP PEI-Cu^2+^, facilitating the formation of Au@Cu core@shell nanoparticles. Although Zhou and coauthors [95] showed the non-linear effect of PEI concentration on the increase of colorimetric signal, there is no experimental comparison of LOD reduction and background coloration for copper enhancement with and without PEI. In some applications, copper sulfate is mixed with the ascorbic acid directly on the test strip [99,100].

Compared with gold and silver enhancement methods, copper enhancement does not have a very long history of use. Kim and coauthors published one of the first articles reporting the use of copper enhancement to amplify colorimetric signal for POC assay [94]. The authors used a dot-blot assay with GNPs and utilized copper enhancement for color amplification. Later, a few articles were published reporting the use of copper enhancement for dot blot assay [92,97]. Only in 2019, Tian and coauthors [99] reported the application of copper enhancement in LFIA for the first time. Since that, very few articles have been published reporting copper enhancement for human gonadotropin and rabbit IgG [99], *E. coli* O157:H7 [95,96], and nucleocapsid of SARS-CoV-2 [100].

**Figure 5:**
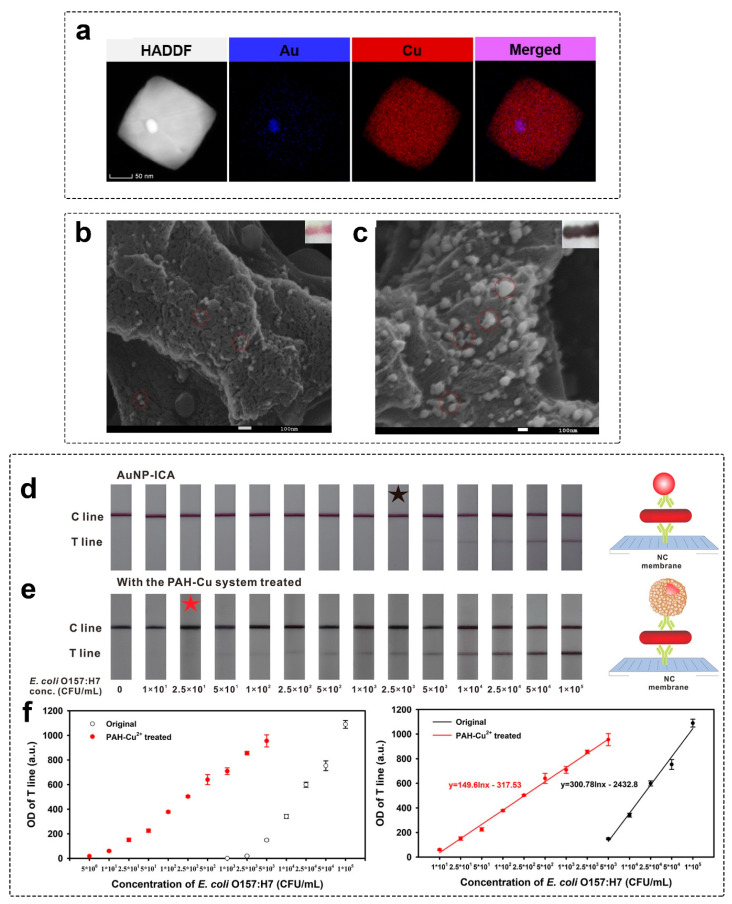
Copper enhancement for LFIA amplification. (**a**) Elemental mapping of GNPs and nanoparticles after copper enhancement [95]. (**b**) Scanning electron microphotograph of nanoparticles before copper enhancement. (**c**) Scanning electron microphotograph of nanoparticles after enhancement. Inserts show the appearance of colored zones on the test strip [99]. Copper enhancement for LFIA of *E. coli*. (**d**) Test strips before copper enhancement. (**e**) Test strips after copper enhancement. Asterisks show the visual LOD values. (**f**) Calibration plot of LFIA before and after copper enhancement [96].

The signal amplification by copper enhancement is similar to that of gold and silver enhancement. The formation of larger core@shell Au@Cu nanoparticles (Figure 5a) facilitates a higher colorimetric signal due to significantly larger scattering than initial GNPs [94]. The membrane affects nanoparticle growth, resulting in the formation of polydisperse nanoparticles (Figure 5b,c). Initially faint-colored/undetectable GNPs can be easily distinguished after copper enhancement on the test strip (Figure 5d–f).

As there are very few articles reporting the use of copper enhancement, no information about inhibitors was published. However, considering the components of enhancing solutions (Cu^2+^ ions and ascorbic acid), OH^−^ ions will interfere with the reaction. Apparently, OH^−^ ions are not applicable for reaction termination, as Cu(OH)_2_ has intense coloration, hindering visual registration of the results.

#### 2.1.4. Galvanic Replacement

Although galvanic replacement is actively used to synthesize nanosized labels for LFIA [101,102,103,104,105,106], only two articles applied galvanic replacement for post-assay signal amplification [40,43]. To perform galvanic replacement, one needs to use particles made of metal with lower reduction potential and ions in the solution with higher reduction potential. As a result, the metal with a lower reduction potential oxidizes and migrates to the solution, while the metal with a higher reduction potential reduces and deposits to the nanoparticles [44]. The signal amplification is based on the change of optical properties, morphology, and size of nanoparticles after galvanic replacement.

Panferov and coauthors [40] performed galvanic replacement for LFIA of the receptor binding domain of SARS-CoV-19. The authors used conventional spherical GNP as the colorimetric label. The post-assay enhancement procedure included two steps. In the first step, silver enhancement was performed, leading to the formation of Au@Ag core@shell nanoparticles. In the second step, the addition of HAuCl_4_ initiated the galvanic replacement of Ag atoms in the shell to Au. As a result, Au@Ag-Au nanoparticles were formed. The authors reported 61 times lower LOD compared with GNPs.

Shu and coauthors [43] applied galvanic replacement in situ for LFIA of salbutamol. Conventional LFIA used CuS nanospheres as the colorimetric label. After the performance of conventional LFIA, a solution of HAuCl_4_ was added, initiating the galvanic replacement of Cu atoms to Au. As a result of the enhancement, the authors reported a two-times-lower LOD value. Although CuS nanoparticles are not a commonly accepted nanolabel, additional studies of their stability during storage and performance in various matrices are required.

Galvanic replacement requires no heating, reducing agents, or catalysts, and can be performed directly on a test strip. The undeniable benefit of galvanic replacement is the high stability of enhancing reagents, namely salts of metals. These salts can be stored in the solution for a long time and do not require temperature control. However, there are two major restrictions on galvanic replacement for post-assay signal amplification.

First, the requirements to use metal ions with higher reduction potentials compared with metals of nanoparticles. GNPs are commonly used as the colorimetric label for LFIA. However, gold has a high reduction potential (Au^3+^ + 3e^−^→Au; reduction potential 1.5 V), and ions of metals (e.g., Ag, Pt, Pd, Cu, Co, and Ni) cannot galvanically replace gold because they all have lower reduction potential. Thus, to apply a galvanic replacement reaction, one needs to use nanoparticles made of metal with lower reduction potential (Ag nanoparticles) or core@shell nanoparticles with a shell made of metal with lower reduction potential. The application of non-gold nanoparticles may be hindered by their poor stability in matrices, non-optimized protocols of synthesis and conjugation, and higher LOD values compared with GNP in conventional LFIA.

Second, there is not enough data confirming that nanoparticles synthesized by galvanic replacement will facilitate significant LOD reduction. Changes in the optical spectra during galvanic replacement were studied in detail for various combinations of metals [107,108]. However, there are no reports assessing the optical properties of initial and synthesized during galvanic replacement nanoparticles in the colloidal solution and colorimetric signal on the membrane. Thus, further fundamental studies of optical, catalytic, and electrochemical properties of nanoparticles synthesized by this approach are required for understanding signal amplification.

### 2.2. Increasing the Number of Labels

This group of methods aims to increase the number (concentration) of labels in the test zone. Two approaches based on chemical reactions can be utilized. In the first approach, the increase in the label concentration is achieved by accumulating the additional amount of labels driven by non-covalent interaction in the test zone. Examples of such interactions are antibody–antigen, antibody–antispecies antibody, and biotin–streptavidin. As a result of such crosslinking, higher numbers of nanoparticles are accumulated in the test zone, multiplying the colorimetric signal. Amplification of the signal is achieved by attaching multiple nanoparticles to a single immunocomplex in the test zone. In the second approach, one of the components in the immunocomplex catalyzes the reaction of the substrate to product conversion [25]. A product of the catalytic reaction is detected. The signal amplification is based on the increased concentration of the label produced by a single catalyst.

#### 2.2.1. Crosslinking of Nanoparticles

In most developments, crosslinking between nanoparticles happens while they migrate through the membrane during the assay. While such implementations are easier for the user (no need for the additional stages), their analytical performance is not often not optimal because of the blocking of binding sites and uncontrollable crosslinking between nanoparticles, leading to high background [109]. To reduce the background, optimization of the nanoparticle concentrations is performed, and usually, relatively low concentrations are selected as optimal [109,110]. As a result, moderate LOD reduction values are achieved. The solution to overcome this limitation lies in the consequent crosslinking of nanoparticles. First, immunocomplexes are formed as in the conventional assay, and only after this, the additional nanoparticles are crosslinked with initial nanoparticles. Thus, post-assay enhancement approaches are methodologically applicable and favorable over conventionally used strategies. The strategy of the consequent complex assembly is typical for many bioanalytical methods, such as ELISA, biosensors, and immunohistochemistry. Immunocomplexes are assembled in multiple stages, and all non-bounded species are washed away during the washing steps. However, for one-step capillary-action driven LFIA, post-assay approaches for crosslinking of nanoparticles are methodologically hindered, and only a few reports have been published recently [111,112,113].

Hendrickson and coauthors reported multilayer GNP assembly directly on the test strip driven by antibody–antispecies antibody binding [112]. After the performance of the conventional assay, the test strip was incubated in the colloidal solution of GNP–antispecies antibodies. After two cycles of layer-by-layer assembly of GNPs, the authors achieved seven times lower LOD of okadaic acid compared to the conventional assay.

**Figure 6:**
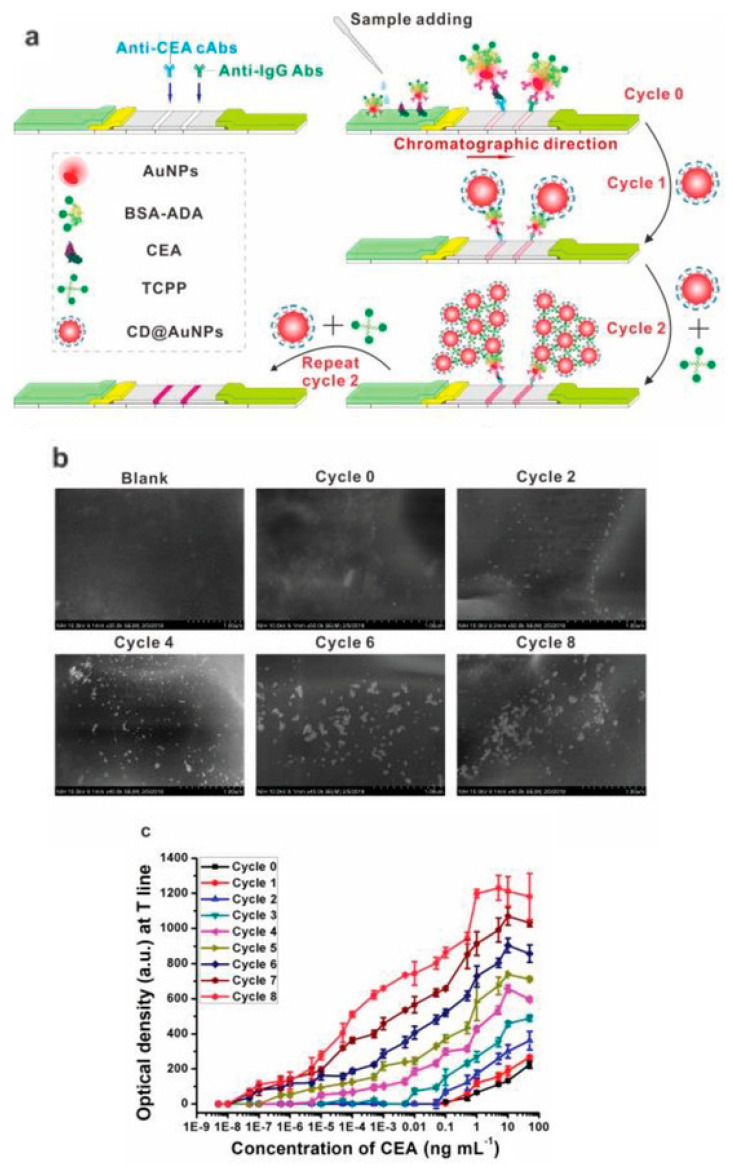
Polylayer GNP assembly for LFIA enhancement. (**a**) The principle of the approach. After completion of conventional LFIA (cycle 0), conjugates of GNP with CD and TCPP are manually added to the test strips, resulting in the polylayer assembly of GNPs. (**b**) Scanning electron microscopy microphotographs of TZ after different cycle numbers. (**c**) Calibration plots after different cycle numbers [113].

Huang and coauthors supramolecular assisted polylayer GNP assembly for highly sensitive detection of carcinoembryonic antigen and HIV-1 capsid antigen p24 [113]. The principle of the method is shown in Figure 6a. For the crosslinking, the authors used GNP modified with β-cyclodextrin (CD) and 1-adamantane acetic acid (ADA) or tetrakis(4-carboxyphenyl)porphyrin (TCPP) (Figure 6a). This host–guest polyvalent recognition results in the tight binding of GNPs and an increase in the number of nanosized labels per single immunocomplex (Figure 6b). For the conventional assay, GNPs conjugated with antibodies and BSA-ADA were used. After completion of the conventional LFIA, the second layer of GNPs was assembled by incubation with CD-conjugated GNP. The further layers (up to eight) were assembled by host–guest interaction of GNP-CD with TCPP. Each TCPP contains four binding sites of CD, facilitating polyvalent binding that results in the accumulation of multiple GNPs.

As a result of such polylayer assembly, a significantly higher coloration of the test zone was achieved (Figure 6c). Using this approach, the authors reported ultra-sensitive detection of carcinoembryonic antigen (0.1 fg/mL in contrast with 0.5 ng/mL for conventional assay) and HIV-1 capsid antigen p24 (0.01 fg/mL in contrast with 0.5 ng/mL for conventional assay). The achieved sensitivity (dozens of proteins per test strip) places the reported LFIA in a row with PCR and facilitates early-stage cancer detection and HIV diagnosis.

However, both articles mentioned above used manually performed cycles of layer-by-layer assembly of GNPs. The procedure requires additional incubation and washing steps. The requirements of multiple manually performed stages may be a significant drawback for real-life applications. Thus, solutions for less laborious signal enhancement are needed. Alternatively, electrophoresis can be used as a driving force instead of capillary actions to perform layer-by-layer assembly of nanoparticles [111,114]. Electrophoresis on the membranes is actively used for various analytical applications [115], and was recently used for post-assay enhancement of LFIA. Panferov and coauthors [111] reported post-assay electrophoresis-driven migration of biotin/streptavidin-modified GNPs for the signal enhancement of hepatitis B surface antigen LFIA. After completion of the conventional LFIA (with biotinylated GNP-antibody conjugate), GNP-streptavidin and GNP-biotin conjugates are electrophoretically dragged across the TZ and CZ. As a result, multilayers of GNPs are assembled by biotin-streptavidin interactions. Electrophoresis combines continuous washing of the test strip and GNP conjugate migration. As a result, the LOD value was reduced from 7.8 ng/mL to 0.12 ng/mL in two minutes, facilitating highly sensitive hepatitis B diagnostics. Further endeavors were dedicated to developing a portable, battery-driven device for electrophoresis [116]. However, despite the rapid procedure and progress in the miniaturization of the power supply, the performance of the assay requires the manual addition of the reagents, which complicates its implementation in POC settings. Further progress is related to the integration of automatic systems of reagent delivery.

Crosslinking of nanoparticles can be driven by various types of molecular interactions. As the binding between nanoparticles is polyvalent (i.e., multiple molecules crosslink nanoparticles), moderate/low affine bindings can be used, as the high stability (i.e., no dissociation of nanoparticles aggregates) will be determined by high avidity [117]. Post-assay crosslinking can be driven by boronate affinity (phenylboronic acid and its derivatives binding with cis-diol-containing molecules such as glycoproteins, glycans) [118], hybridization of nucleic acids [119], vancomycin-D-alanyl-D-alanyl fragment in Gram-positive bacterial cell wall [120], barnase–barstar [121], small molecule–protein [122] and other types of binding.

#### 2.2.2. Catalytic Accumulation of the Label

This group of methods is based on the transformation of the substrate into the product by enzymes and enzyme-mimicking nanoparticles (nanozymes) [123]. The signal enhancement is based on the generation of multiple molecules of detectable product by a single catalyst unit. As the colorimetric detection of peroxidases (predominantly horseradish peroxidase—HRP) and peroxidase-mimicking nanozymes is mainly implemented in LFIA enhancement articles (as well as in ELISA), our consequent speculation about LOD reduction will be performed on this example.

The concentration of the product ($\left[P\right]$, M) is determined by the catalyst’s activity (turnover number, $k_{cat}$, s^−1^), catalyst’s concentration ($\left[N_{o}\right]$, M), and the catalytic reaction time (*t*, s) (Equation (4)):

(4) $$\left[P\right]=k_{cat}\times [N_{o}]\times ∆t$$

Considering the Michaelis–Menten model of catalysis and assuming that the product can be registered in concentrations of the order 10^−6^–10^−7^ M (molar extinction coefficient for peroxidase substrates varies 10,000–39,000 M^−1^ cm^−1^ [124], facilitating reliable optical density ≥ 0.05 a.u following the Beers–Lambert law), $k_{cat}$ for HRP and most nanozymes is in the range 10^3^–10^5^ s^−1^ (Figure S1, Table S1), the reaction time is limited to 300 s, and the lowest detectable amount of catalyst can be estimated in the range (Equation (5)):

(5) $$\left[N_{o}\right]=\frac{\left[P\right]}{k_{cat}\times ∆t}\approx 10^{-12}–10^{-15}\;M$$

Further recalculations to the numbers of HRP molecules/nanozymes particles in the test zone (assuming the volume in the test zone is on a microliter scale) demonstrate that catalytic amplification facilitates the detection of 10^2^–10^5^ catalytic particles. Considering the fact that each nanoparticle can borrow multiple HRP molecules [125] and each nanozyme particle contains multiple catalytic sites on its surface [126], the estimated number of particles can be even one or two orders of magnitude lower. Compared with conventionally used 20–40 nm spherical GNPs, catalytic amplification may facilitate lower LOD from one to four orders of magnitude [23]. Although these calculations were made with many assumptions, they still allow us to show the benefits of catalytic signal enhancement and roughly estimate the expected LOD reduction range.

##### Enzymes as the Catalytic Labels

Enzymes have been extensively used for signal enhancement/generation in ELISA and biosensors. The application of enzymes for LFIA is a logical continuation of the developments in these areas. The first use of enzymes in paper-based immune tests was reported in 1985 by Zuk and co-authors [127]. The authors used paper test strips covered with antibodies against theophylline and conjugates of theophylline with glucose oxidase and HRP. These conjugates and free theophylline compete for binding with antibodies on the test strips. Afterwards, the substrates for both enzymes (glucose for glucose oxidase and 4-chloro-1-naphthol for HRP) were added. This paper is also notable as it introduced the concepts of cascade catalysis (products for one enzyme are the substrates for another) and in situ generation of hydrogen peroxide.

**Figure 7:**
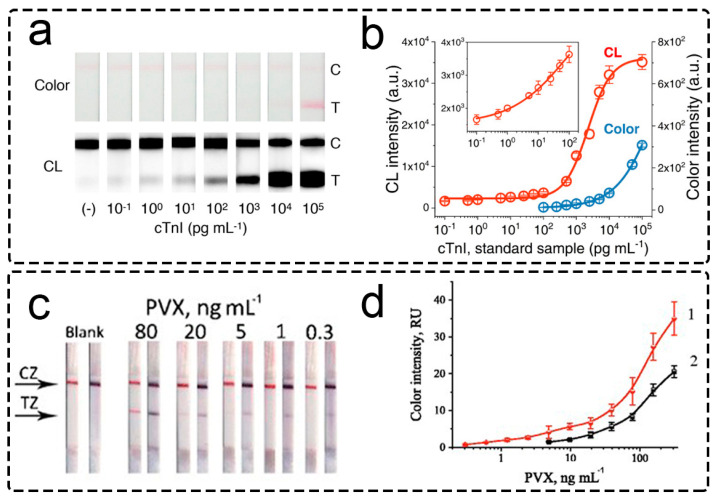
Enzyme-assisted signal amplification in LFIA. (**a**) Test strips with colorimetric and chemiluminescent signals registered after LFIA of various concentrations of human cardiac troponin I; HRP was used as the catalytic label. (**b**) Calibration plots for test strips shown in (**a**); [125]. Colorimetric signal amplification catalyzed by ALP. (**c**) Pairs of test strips (before and after ALP-catalyzed enhancement, respectively) after LFIA of potato virus X. (**d**) Calibration plots for test strips shown in (**c**) [132].

Since then, many articles have used HRP [128,129,130] and alkaline phosphatase (ALP) [131,132,133] for signal generation/enhancement (Figure 7). Chemiluminescent (luminol with hydrogen peroxide, Figure 7a,b) [129,134] and colorimetric substrates (3-amino-9-ethylcarbazole (AEC) [135], soluble [136] and insoluble [137] 3,3′,5,5′-tetramethylbenzidine (TMB), 3,3′-diaminobenzidine (DAB) without [138] and with metal enhancement [139]) were used for the signal enhancement for HRP. The used colorimetric substrates of ALP were limited by 5-bromo-4-chloro-3-indolyl phosphate with nitro blue tetrazolium (BCIP/NBT, Figure 7c,d) [132,133]. Shu and coauthors [140] demonstrated that ALP and HRP could be simultaneously detected due to the kinetics difference in substrate oxidation. After the performance of the assay, the test zone was cut and placed into a microwell plate with the combined substrate. Using time-resolved chemiluminescent detection, the authors recorded chemiluminescent signal at 2.5 s (for HRP) and 300 s (for ALP). As a result, the test strip with a single test zone can be used for the simultaneous detection of two pesticides.

The selection of enzymes for signal enhancement is determined by the analytical performance of conventional LFIA (required LOD reduction, background level) and endogenous enzyme activity in matrices. Considering the kinetics of colorimetric substrate oxidation, HRP has a higher turnover number than ALP. Although mutant forms of ALP with high turnover number values were reported [141,142], most analytical papers used commercially available mammalian ALP. Following Equation (4), ALP generates a lower amount of the product per time. Combined with the fact that ALP has significantly higher molecular weight than HRP (140–160 kDa for bovine alkaline phosphatase [143,144], 40 kDa for HRP [145]), the lower amount of ALP can be conjugated to the single GNP [146]. As a result, ALP usually generates lower product concentration and requires longer incubation time. Thus, HRP seems the preferable enzyme in the first approximation. However, the higher catalytic activity of HRP may cause issues with the background. Non-specifically bounded HRP generates high background, hindering the visual/instrumental signal registration. Thus, the application of HRP may require a more thorough optimization of buffer composition, blocking protocols, etc. The endogenous enzyme activity of matrices may significantly interfere with signal amplification [147,148,149]. Thus, for assays in matrices with high endogenous peroxidase activity (e.g., plant extracts), ALP is recommended [150,151]. The activity of enzymes is affected by the inhibitors in samples. For HRP-assisted signal enhancement, excluding sodium azide and using alternative bacteriostatic agents is highly desirable. ALP is inhibited by phosphate and pyrophosphate. Thus, for ALP-assisted signal enhancement, phosphate-containing buffers should be avoided.

The low stability of substrates can be a bottleneck of enzyme signal enhancement for POC conditions. Many efforts have been dedicated to stabilizing and increasing substrates’ shelf lives [152,153]. Some commercial products were developed (e.g., ELISA TMB Stabilized Chromogen or 1-Step Ultra TMB-Blotting Solution from ThermoFisher(Pierce Biotehnology, Rockford, IL, USA). However, most of the articles use freshly prepared substrates. Focusing on HRP-assisted signal amplification, stabilizing a two-component substrate requires separate storage of hydrogen peroxide and the second component (TMB, DAB, luminol). Storage of dry substrates will significantly simplify the assay performance in POC. TMB/DAB/luminol can be dried on the membrane and rehydrated during analysis. Hydrogen peroxide is formed in situ before/in parallel with enzyme signal enhancement. For example, urea peroxide is a dried chemical that forms hydrogen peroxide in situ after contact with a buffer [154,155]. Alternatively, hydrogen peroxide can be formed as a product in enzymatic reactions. This approach was shown for the first time in the pioneering work of Zuk and co-authors [127], where glucose was oxidized by glucose oxidase by oxygen with the formation of hydrogen peroxide. In further developments, Min-Gon Kim’s group developed an approach for in situ hydrogen peroxide formation by oxidation of choline by choline oxidase [156,157]. Such in situ substrate formation has the potential for POC conditions, especially combined with integrated signal enhancement systems (Section 3).

##### Nanozymes as the Catalytic Label

Nanozymes are nanoparticles with enzyme-mimicking activity. Nanozymes were introduced in 2007 in reports of the peroxidase-mimicking activity of Fe_3_O_4_ nanoparticles [158]. Since then, many inorganic materials with enzyme-mimicking properties have been reported (Figure S1), and nanozymes found a broad application in various analytical methods [159,160]. Nanozymes consisting of metals of the platinum group [161,162,163] and iron-containing nanozymes [164,165,166,167] are mainly considered in LFIA. Predominantly peroxidase-mimicking nanozymes are used in LFIA, while the application of oxidase-mimicking and catalase-mimicking nanozymes is also actively developing. Similar to enzymes, various organic substrates were used for peroxidase-mimicking nanozymes, including colorimetric (soluble [168] and insoluble TMB [162], 3-amino-9-ethyl-carbazole (AEC) [169], conventional [170], and metal-enhanced DAB [171]) and luminescent (luminol [172]) ones.

Oxidase-mimicking and catalase-mimicking enzymes can be beneficial because they can be detected using one-component substrates. Oxidase-mimicking nanozymes (Au@Pt nanorods [173], CeO_2_ nanoparticles [174], and MnO_2_ nanosheets [175]) catalyze the oxidation of TMB without hydrogen peroxide. Unlike peroxidase and oxidase-mimicking nanozymes, catalase-mimicking nanozymes catalyze reactions without forming colored products. Instead, the detection of oxygen formation is performed using a handheld pressure meter [176,177], by measuring the height of foam in detergent filled tube [178,179], by monitoring disposable syringe pistol displacement [180], or by monitoring 3D pressure-based foam resistance [181].

**Figure 8:**
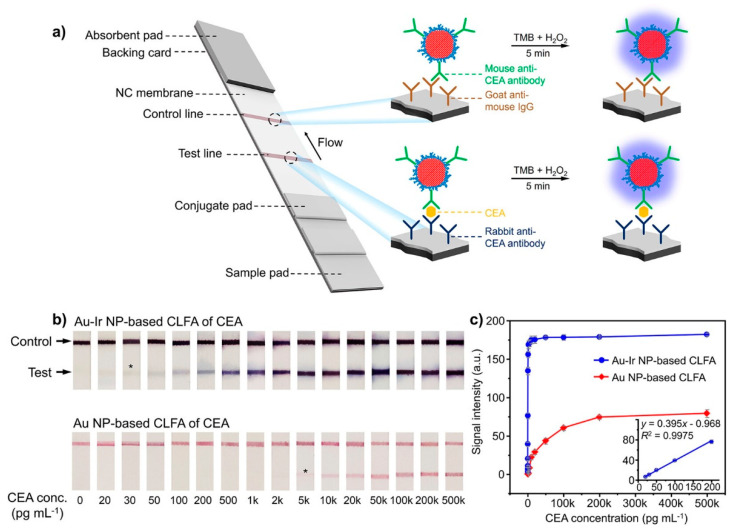
LFIA with nanozyme signal amplification. (**a**) Principle of signal amplification. After the performance of conventional LFIA, TMB substrate is added, and the nanozymes in test and control lines catalyze accumulation of colored product. (**b**) Test strips after nanozyme signal amplification (**top**) and conventional GNPs (**bottom**). Asterisks show the visual LOD values. (**c**) Calibration plots for conventional and nanozyme signal enhancement [182].

The current trend in nanozyme-based signal amplification in LFIA is the selection of the most catalytic active nanoparticles (Figure 8) [182], although one needs to understand that the high catalytic activity of nanozymes does not guarantee the high sensitivity of LFIA. Matrix effect, and the reduction in catalytic activity after conjugation and during storage, may negate the high catalytic activity of nanozymes (usually determined for bare particles) and facilitate mediocre sensitivity. Thus, the experimental confirmation of a nanozyme’s benefits in LFIA is crucial.

Wei and coauthors used Ir-coated GNPs as the colorimetric and catalytic labels [182]. Ir-coated GNPs demonstrated 10-times-higher catalytic turnover for TMB than Pt-coated GNPs (10^7^ s^−1^ and 10^6^ s^−1^, respectively). After functionalization with monoclonal antibodies against carcinoembryonic antigen, the nanozymes were used for sandwich immunoassay (Figure 8a). After conventional LFIA, the colorimetric signal was amplified by the oxidation of TMB by hydrogen peroxide catalyzed by the nanozymes. LFIAs with GNP and Ir-coated GNPs were compared (Figure 8b). The authors reported significantly lower LOD for Ir-coated GNPs after enhancement compared with conventional GNPs (Figure 8c, 7.8 pg/mL and 1.26 ng/mL of carcinoembryonic antigen, respectively). It is notable that LOD values for Pt-coated GNPs were ∼3.3 times higher compared with Ir-coated GNPs, which can be explained by the superior catalytic activity of Ir-coated GNPs. The beneficial performance of Ir-coated GNPs was confirmed using a prostate-specific antigen.

Panferov and coauthors also studied the relationship between the catalytic activity of nanozymes and LOD values of LFIA [171]. Fifteen Pt-coated GNPs with Pt concentration from 0 to 2 μM and peroxidase-mimicking activity varied from 0 to 4.39 ± 0.4 U/mg were synthesized. The LOD values were directly related to the catalytic activity of nanozyme and were varied in the range of 10^5^ CFU/mL to 3 × 10^2^ CFU/mL of phytopathogenic bacteria.

Nanozymes are often considered the functional replacement of enzymes in bioassays [183]. Being artificial nano-sized catalysts, nanozymes possess properties of both enzymes and nanoparticles. Such combinations make nanozymes potentially more applicable labels for LFIA than enzymes.

The first distinctive property of nanozymes is the tunability of their optical and catalytic properties. Many approaches for the synthesis of nanozymes with tunable absorbance wavelength [101,102,184] and catalytic activity [162,171] have been developed. These synthetic approaches are based on the precise control over the size and morphology of nanoparticles [185], the chemical composition of nanoparticles [186], and their surfaces (atomic-thin surface-dispersed active centers [162], single-atom nanocatalysts [187]). Such flexible and on-demand structural properties tuning functional properties are unachievable for enzymes [188,189,190].

The second distinctive property of nanozymes also arises from their morphology. A single nanozyme particle can be conjugated with dozens/hundreds of functional molecules (e.g., antibodies, aptamers, enzymes, etc.). In contrast, the number and availability of functional groups limit the conjugation of receptor molecules to enzymes. For example, HRP contains only two primary amine groups available for conjugation [191]. Direct conjugation of HRP with IgG using bifunctional crosslinkers resulted in a low conjugation yield of target HRP-IgG (around 2%) with the excessive formation of IgG polymers [192]. Thus, alternative and more sophisticated methods are used. The commonly used method includes the oxidation of carbohydrates in HRP by sodium periodate and consequent reductive amination with the protein [192]. However, this method is laborious, may be associated with the use of highly toxic cyanoborohydride, and often results in aggregation/precipitation of HRP conjugates [191]. Nanozymes can be conjugated with functional molecules by physical adsorption or covalent binding [193]. Physical adsorption is arguably the easiest method for the conjugation; it was used for binding antibodies to Pt nanoparticles [194], Pt-coated GNPs [195], Fe_3_O_4_ coated with polydopamine [196], VS_2_ nanosheets [197], and Prussian blue nanoparticles [198]. Covalent coupling uses bi-functional crosslinkers (usually containing sulfhydryl and carboxyl groups) and reagents for carbodiimide chemistry [199,200,201]. The concentration of receptors immobilized on nanozymes can be optimized to achieve the highest sensitivity of the assay [202]. It is noteworthy that the presence of immobilized molecules may significantly reduce catalytic activity due to the surface shielding effect [203,204]. Further in this paper, we briefly discuss this effect while considering measurements of the Michaelis–Menten constant for LFIA optimization.

The third distinctive property of nanozymes is their multifunctionality. As mentioned, nanozymes can serve as optical labels, carriers of functional molecules, and catalytic labels. Nanozymes containing Fe_3_O_4_ can be used for magnetic enrichment of target [198,205,206,207], facilitating an additional tool for LOD reduction and reducing the interference of matrix components. Finally, some articles demonstrated that Fe_3_O_4_ nanozymes could be detected using a magnetic signal reader [206] or photothermal detector [208,209]. Although there are relatively few papers published about these detection methods, their application may be promising for highly sensitive LFIAs. Registration of the magnetic signal is more beneficial than the colorimetric signal, as it facilitates the detection of nanoparticles within the total thickness of the membrane [21,210]. Photothermic registration is a promising approach, as it can be used to register nanoparticles in immunocomplexes [22,208] and increase the peroxidase-mimicking activity of nanozymes [211].

The fourth distinctive property of nanozymes is the outstandingly high stability of their catalytic properties at elevated temperatures [172,195], at high concentrations of substrates [194,212] and inhibitors [171], and over a broad pH range [213,214]. Panferov and coauthors used such high stability of peroxidase-mimicking properties of Au@Pt nanoparticles for signal amplification in plant extracts with high endogenous peroxidase activity [215]. All endogenous peroxidases in extracts were inhibited by elevated H_2_O_2_ concentration (>20 mM), while Au@Pt nanozymes maintained activity in 100-times-more-concentrated H_2_O_2_. As a result, significantly lower background staining was observed, and high LOD reduction was achieved. The use of high-stability nanozymes for post-assay amplification is favorable, as all immunocomplexes are already formed, and harsh conditions can be applied.

Actually, there is no “best-suited” nanozyme for LFIA (as opposed to ELISA, where almost all articles and commercial kits use HRP). Following the speculation based on the turnover number (Equations (4) and (5)) and experimental data [171,182], the obvious recommendation is to use nanozymes with the highest catalytic activity. As the catalysis occurs at the surface-exposed sites of nanozymes, the catalytic activity of nanozymes depends on the size and shape of nanoparticles. It was shown for nanozymes of various chemical nature (Fe_3_O_4_ [158], Prussian Blue [216], CeO_2_ [217], Pd [218]), the smaller particles tend to have higher catalytic activity compared with larger ones because of higher surface area (for the sane mass of material). Similarly, for nanozymes of various morphology and porosity, the particles with higher surface area show higher catalytic activity [219,220,221]. However, as there is no one accepted method for measuring nanozymes’ catalytic activity, comparison of activities reported in different articles can be challenging. The discussion about the calculation of nanozymes’ catalytic activity is still continued [126,222,223]. Jiang and coauthors proposed the detailed protocol for measuring the catalytic activity and kinetics of peroxidase-mimicking nanozymes [124]. In the reported protocol, the specific peroxidase-mimicking activity is recalculated to milligram of nanozyme. Recalculation of the activity to the unit of mass is applicable to enzymes, where each molecule is an active catalyst. However, using a similar recalculation for nanozymes is questionable. Nanozymes can be quantified based on the concentration/mass of particles (each nanoparticle is considered a nanozyme unit), the concentration of components of particles (each atomic component is considered a nanozyme unit), or the concentration of surface-exposed components (each surface-exposed component is considered a nanozyme unit) [223]. As reported by Zandieh and Liu [126], for 300 nm Fe_3_O_4_ nanoparticles, only 0.32% of the total Fe atoms are surface-exposed and can catalyze the reaction. Thus, the quantification of nanozymes based on their mass may cause a significant underestimation of activity. Because of the lack of a commonly accepted protocol for measuring nanozymes’ catalytic activity, *k_cat_* may be varied by six orders of magnitude! (Figure S1, Table S1). The uncertainty of nanozyme activity quantification goes beyond the application in LFIA and requires fundamental studies of the reaction mechanism, and the evaluation and quantification of active sites on nanozyme surface.

However, the lack of consistency in nanozyme quantification does not hinder the development of enhanced LFIA. While the proper kinetic characterization of nanozymes remains an important fundamental task, the developers and users of LFIA are interested in LOD values before and after catalysis. To avoid misinterpretation of kinetic data, the performance of nanozymes as the catalytic label in LFIA should be evaluated by comparing LOD values after and before catalysis [171,182,224]. Despite the issues with accurate quantification of nanozymes’ activity, at least the Michaelis constant (*K_m_,* M) should be measured for developing nanozyme-enhanced LFIA. Such measurements are often ignored for enzymes, as the new developments rely on previously optimized protocols. However, the kinetic parameters of nanozymes vary significantly from enzymes. The values of *K_m_* for H_2_O_2_ for nanozymes can be two to four orders of magnitude higher than HRP [158,194,225]. Conducting the signal enhancement using non-optimized concentrations of H_2_O_2_ (borrowed from HRP protocols) will result in the performance of enhancement in non-optimal conditions. Although the difference in *K_m_* values for TMB between HRP and nanozymes is usually less significant, the kinetic measurements for both substrates are highly recommended. Similar to the recommendations for measuring the enzyme’s kinetics [226], the optimal concentrations of substrate for nanozymes should be in the range of 0.5 × *K_m_* to 5 × *K_m_.* [124]. The measurement for *K_m_* can be performed without disputable quantification of nanozyme units and will ensure that enhancement is performed in optimal conditions. Some protocols report the use of two-to-three-order-of-magnitude-higher concentrations of H_2_O_2_ in the substrate solution for LFIA enhancement compared with conventional ELISA [169,224,227]. The presence of adsorbed molecules on the surface of nanozymes significantly reduces catalytic activity [203,204]. The adsorbed molecules shield the nanozyme surface, thus reducing substrate availability and increasing the *K_m_* value [228]. Therefore, kinetic characterization should be performed with the conjugated nanozymes, not bare particles.

**Figure 9:**
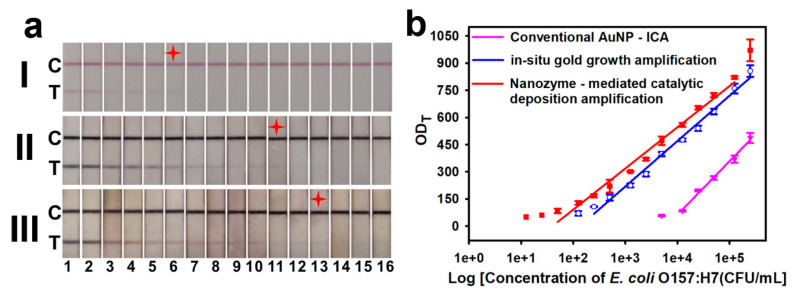
In situ restoring of Au nanozyme surface. (**a**) Test strips after conventional LFIA with GNP before gold enhancement (I), after gold enhancement (II), after gold enhancement and catalysis (III). Red asterisks show the visual LOD values. The numbers below test strips correspond to *E. coli* concentration from 2.5 × 10^5^ (1) to 50 CFU/mL (15) and negative control (16). (**b**) Calibration plots of three LFIAs shown in panel (**a**) [230].

The surface recuperation method can be used to restore the nanozyme activity after conjugation [177,229,230]. Surface recovery is performed in a post-assay manner and includes the chemical reaction for coating seed particles with bare metal surface. This approach was used for the deposition of Pt-layer and in situ synthesis of catalase-mimicking nanozymes for ELISA-like [229] and LFIA [177]. Fu and coauthors [230] proposed a two-stage post-assay enhancement approach that combines the consequent performance of gold enhancement and the peroxidase-mimicking activity of enlarged bare GNPs (Figure 9). As a result, the LOD value for *E. coli* O157:H7 was reduced from 5 × 10^3^ CFU/mL to 1.25 × 10^1^ CFU/mL for conventional GNP and nanozyme-enhanced LFIAs, respectively (Figure 9b).

Further anticipated progress in nanozyme-assisted signal enhancement in LFIA is related to the development of more catalytically active and multifunctional nanozymes. These directions partly align with the developments of fundamental nanozymology [183]. Increasing the catalytic activity can be achieved by optimization of the chemical composition of nanozymes [102,171,188,231], creating surface-dispersed catalytic centers [101,162] or single-atom catalysts [232,233]. Nanozymes with surface-dispersed catalytic centers [234] and single-atom catalysts have high catalytic activity and low consumption of precious precursors [235], making them a prospective label for LFIA and other bioanalytical methods.

## 3. Post-Assay-Integrated Signal Amplification

The predominant part of the described post-assay amplifications is performed manually. The analyst adds the enhancing solution to the test strip and monitors the signal growth. Some protocols require washing the test strips before and/or after signal amplification. Such manually performed enhancement protocols are acceptable for proof-of-concept experiments. However, any add-ons to the straightforward, one-step LFIA procedure are considered a drawback for real-life applications. The ultimate goal is to create a flawless procedure between the conventional LFIA and signal amplification—i.e., the integration of the enhancement step to the LFIA procedure. The performance of such LFIA with integrated enhancement should be similar to the conventional assay. The user adds a few drops of the sample to the test strips and detects the results after 10–15 min. Further in this paper, we review the major achievements in this area and discuss the limitations of the integrated enhancement approaches.

**Figure 10:**
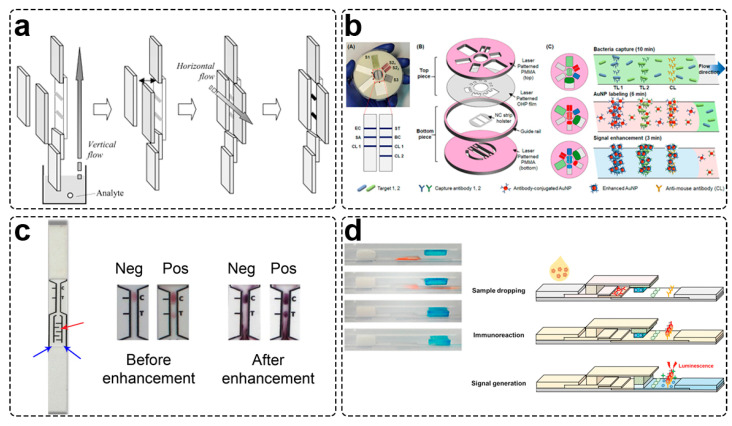
Various approaches for post-assay integrated signal amplification. (**a**) The use of additional membranes maintains the migration of enhancing reagents [136]. (**b**) Hand-driven rotatory device for the consequent delivery of immunoreagents and enhancing reagents [241]. (**c**) The test strip with wax-printed barriers for consequent delivery of immunoreagents and enhancing reagents [242]. (**d**) The 3D system with an additional membrane that comes into contact with the test strip and initiates migration of enhancing reagents after completion of conventional LFIA [157].

The attempts to integrate the signal amplification into the LFIA procedure were reported in the early developments. Se-Hwan Paek’s group reported multiple LFIAs with cross-flow design for enzyme signal amplification [136,236,237,238,239] and silver enhancement [240]. The typical design (Figure 10a) includes a vertically arranged test strip and the additional pads with the enhancement components applied horizontally. After the completion of the conventional LFIA, the horizontal pads were connected to each side of a test strip. The enhancement components were rehydrated and migrated horizontally through the test and control zones by capillary forces. Furthermore, this group developed a fully integrated enhanced LFIA, including portable chemiluminescent [237,239] and colorimetric [240] detectors. Such an implementation with fully integrated LFIA could be practically demanded as it maintains all the benefits of conventional qualitative LFIA and requires only minor and portable accessories.

Additional pads were also used for the storage of enhancing reagents. Storage in dry form can be used to increase the stability of enhancing reagents. Deng and coauthors reported the use of sodium perborate on the membrane as a way to avoid low-stable hydrogen peroxide [243]. After the performance of the conventional LFIA, a fiberglass membrane with dried luminol and sodium perborate is placed on the test strip, and a drop of water is added to initiate hydrogen peroxide formation in situ. The additional membranes with dried reagents were also reported for silver enhancement [81,244,245]. Wei and coauthors used two additional glass fiber membranes soaked with AgNO_3_ and hydroquinone [244]. After the completion of the LFIA, the test strip was covered with the fiberglass membranes, and water was added to rehydrate the silver-enhancing reagents. Although these protocols with the additional membranes simplify the amplification procedure to some extent, the enhancement procedure is not fully integrated and requires manual operations.

The next step to post-assay integrated enhancement is the combination of LFIA with hand-powered microfluidics. For such implementations, microfluidics chips serve as a tool for delivering signal enhancement components. Zangheri and coauthors used a polymeric cartridge that contains all reagents to detect HRP-catalyzed chemiluminescence on the test strip [246]. After the conventional LFIA is completed, the user simultaneously pushes reservoirs with hydrogen peroxide and luminol. The enhancement components are mixed and incubated on a test strip to initiate a reaction of signal enhancement. Later, these authors used a cartridge for the performance of an automatic HRP-catalyzed chemiluminescent LFIA for salivary cortisol detection onboard the International Space Station [247]. The cartridge contained all reagents, and the astronaut performed the assay by a simple sequence of manual operations (screwing and unscrewing of nylon screws). The developed device had a chemiluminescent reader and could facilitate the screening from sample preparation to the quantitative result. The integration of the LFIA test strip into a microfluidic cartridge was used for HRP-catalyzed chemiluminescent detection of ochratoxin A [248]. HRP-conjugate and substrates are delivered by pressing the chambers on the cartridge and squeezing the reagents into the test strip. A similar approach with a manually squeezing enhancing solution was implemented for silver enhancement of LFIA for tuberculosis diagnostics [89]. The Fujifilm SILVAMP TB LAM test system uses a cartridge containing the test strip and reservoirs with silver-enhancing reagents. After performing a conventional assay, the user manually presses the buttons on the chip, causing the release of enhancement components to the test strip (video protocol is available as a supplementary video [89]). Zhang and coauthors developed a LFIA which requires the user to press the button to start signal amplification [154]. By pressing the button, the test strip bends and comes into contact with the substrate solution. The device developed by Shin and Park allows the user to deliver multiple reagents (for assay performance, washing, and HRP-assisted signal enhancement) by manually rotating a lid around a stationary test strip [249]. By rotating the device, the test strip aligns with the reagents and adsorbent pads, initiating the fluid flow. Further improvement (Figure 10b) allows multiplex detection of foodborne pathogenic bacteria with gold enhancement. Consequent delivery of samples, conjugates, and enhancing reagent is achieved by hand-driven rotation of the device lid [241]. The reported microfluidics devices were used for the delivery of quite large volumes of enhancing solutions (40–90 µL) comparable with the volumes added manually to the test strip. Microfluidics were utilized only as the tool for the reagent delivery and did not serve additional goals such as assay miniaturization and reducing the operating volumes of reagents/samples.

Important to note is that all these developments used hand-powered microfluidics. Pumping is performed by pressing or squeezing reservoirs with the enhancement components. Although the control over liquid migration (volume, rate of migration, mixing) is poor compared with conventional pump-driven microfluidics, the user-friendliness outweighs the disadvantages. Although combining LFIA with microfluidic cartridges does not allow the performance completely automatic enhancement procedure, this approach seems an acceptable trade-off between the benefits of highly sensitive LFIA and the drawback of the additional manipulations. This approach was successfully implemented in non-laboratory conditions by testing saliva cortisol on board the International Space Station [247] and field diagnostics of tuberculosis [89], indicating that practitioners may admit its usage. Some microfluidic devices can be used for the performance of multiple assays, which makes them practically feasible.

In parallel with microfluidic chips, paper microfluidic devices were actively developed. Paper microfluidic devices are actively used for various analytical tasks. In this review, we focus on two-dimensional [155,250] and three-dimensional [125,251] paper microfluidics for delivering the enhancing reagents. Two-dimensional systems are based on controlling the hydrodynamic flow rate [64,252,253]. Such 2D systems facilitate controllable migration rates of multiple flows on a single device. Yager’s group in multiple publications reported the use of two-dimensional paper networks for the consequent delivery of reagents for gold enhancement [254,255,256]. Two-dimensional paper networks are add-ons connected to conventional test strips that act as multiple inlets, facilitating the delivery of sample, immunoreagents, washing buffers, and gold-enhancing reagents. The consequent delivery of the reagents can be achieved by the geometry of the paper network and by creating dissolvable sucrose barriers [257]. The higher the sucrose concentration, the more time is required to hydrate and overcome the barrier. The consequent delivery of the reagents can be achieved on a single test strip without add-ons by creating channels with various widths/path lengths. Panraksa and coauthors used wax printing to create delayed and non-delayed channels (Figure 10c) [242]. First, the sample migrates through the non-delayed channel (Figure 10c, shown with two blue arrows), initiating immuno-complex formation. The central channel with multiple baffles (Figure 10c, shown with the red arrow) was designed to facilitate the delayed migration of gold-enhancement components to the test zone. As a result, first immunoreaction in the test zone occurs, and then the signal is amplified by gold enhancement. The test strip contains all reagents for immunoreaction and gold enhancement in a dry form. Thus, the performance of the enhanced assay is similar to the conventional assay. No additional washing steps, incubations, pads, and solutions are required. Such integrated test strips facilitating the consequent reagent delivery for assay performance and signal amplification in “one touch” can be considered for practical applications [57,242,258,259].

The “one-touch” silver enhancement protocol was proposed by Kim and coauthors [260]. The authors used coaxial electrospinning to form core–shell nanofibers containing a water-soluble polymeric shell and silver enhancement components in the core. When the sample migrates through the membrane, it dissolves the soluble shell, resulting in a time-delayed release of silver-enhancing reagents. As a result, immunocomplexes are formed in the test zone first, and only after that, the enhancing reagents are delivered. The authors used commercial test strips and achieved 10-times-lower LOD.

Three-dimensional microfluidic systems utilize a more sophisticated device architecture to perform sequential delivery of reagents. Min-Gon Kim’s group proposed a new approach for sequential, time-delayed, and automatic reagent delivery to the test strip [157]. The authors used an additional pad with the enhancing reagents that can physically change position and come in contact with the test strip after 6 min of sample application (Figure 10d). The automatic change of position occurred due to the expansion of water-swellable tape. This approach was utilized in their further developments [125]. The authors showed that this approach could be used for the automatic delayed delivery of the enhancing reagents to perform gold enhancement, HRP, and ALP-catalyzed signal amplification.

Driven by capillary action, paper microfluidics does not require any additional instruments or manipulation. However, the practical affordability of such assays is questionable. While the construction of 2D and 3D paper microfluidics is relatively performable in laboratory conditions, its massive production for commercial test systems is doubtful. Even minor deviations from the well-established production procedures will require integrating new equipment into the existing in-lines.

A critical feature of integrated signal amplification systems that often remains overlooked is the hindered ability to control the time of the enhancement reaction. When enhancing reagents are incubated on the test strip, the user controls the reaction time with a stopwatch. Also, the enhancing reagents are added in excess (by volume and concentration), and their concentrations do not limit the reaction. For integrated signal enhancement (primarily for embodiments with delayed reagents delivery), the control over the duration of “residence time” of enhancing reagent with target zones on the test strip is hindered. The user cannot precisely determine the start (when enhancing reagents were delivered) and end (when enhancing reagents were washed) of the enhancement. As a result, the duration of the enhancement procedure may vary from test strip to test strip. Often, the enhancement time is not even reported by authors. Also, the delayed delivery of enhancing reagents with the additional flow may cause potential bias. Laminar migration of enhancing reagents does not allow adequate mixing. Thus, gradients of concentrations of enhancing reagents during their rehydration are expected along the flow. As a result of these two biases, the enhancement may be less reproducible and facilitate lower LOD reduction. Comparative studies of analytical performance (time, LOD, background) of various integrated signal amplification and conventional incubation with enhancing reagents are needed.

## 4. Comparison and Assessment of the Enhancement Approaches

After discussing the existing post-assay enhancement approaches, we focus on the comparative assessment of these approaches and discuss their applicability to POC conditions. We mention some limitations of the enhancement approaches and the idea of post-assay enhancement in general and end up with proposals for overcoming these limitations.

### 4.1. Quantitative Evaluation of the Improvements Reached by Different Enhancement Approaches

**Figure 11:**
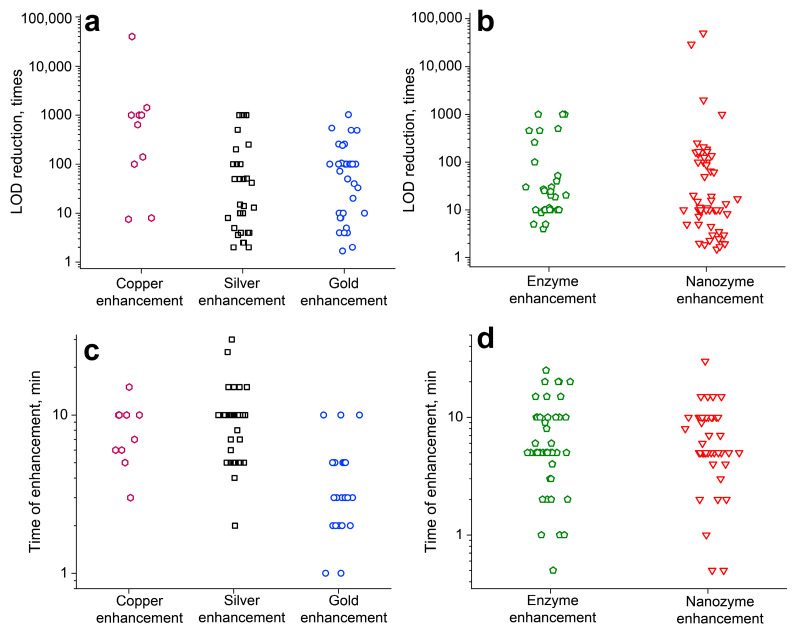
Plots demonstrating the comparable performance of various post-assay enhancement approaches. (**a**,**b**) LOD reduction values. (**c**,**d**) Time of enhancement. Data for (**a**–**d**) are summarized in Tables S2 and S3.

Although the signal amplification mechanisms are different, the enhancement approaches can be compared using quantitative parameters such as LOD values and time of enhancement. The comparison of the values of colorimetric signals cannot be performed reliably between articles, as the authors use various ways to record and quantify these signals. Having reviewed the articles published by June 2023, we selected 130 articles reporting LOD enhancement (Table S2) and 151 articles reporting amplification time (Table S3). For the comparative assessment of the enhancement approaches, we used LOD reduction values (Figure 11a) and time required for enhancement performance (Figure 11b). The absolute LOD value is determined by the affinity of immunoreagents, types of membranes, and conditions of LFIA. Thus, we argue that only the comparison of LOD reduction values, not absolute LOD values, is acceptable. We collected only LOD reduction made by using the same immunoreagents. We did not use the data where the authors reported the LOD reduction compared with the commercial LFIA or with literature-reported results with different immunoreagents. The comparison of LOD values with ELISA (even made with the same reagents) was not used. Also, we compared only LOD obtained in the same sample composition (buffer or matrix). The selected methodology facilitates the evaluation of the impact of the enhancement approach only, without biases arising from the affinity of reagents, the influence of matrix components, etc.

The time of chemical enhancement was mined from the materials and methods section. Only enhancement time (not the whole assay time) was collected. The results are summarized in Figure 11 and Tables S2 and S3.

Most articles report LOD reduction in the range of one to three orders of magnitude (Figure 11a,b). Such a significant LOD reduction is usually achieved in less than 10 min (Figure 11c,d). None of the approaches is beneficial in terms of LOD reduction. One needs to consider all advantages and limitations (Table 1) while selecting the enhancement strategy in each case (e.g., type of matrix, required LOD value, target audience, cost of the enhanced assay, etc.). The availability of reagents, properties of matrices, type of target, and many other parameters dictate the selection of the enhancement approach. The area of post-assay signal amplification is still actively developing. For example, the post-assay crosslinking approach (Section 2.2.1) was introduced recently, and all three articles [111,112,113] were published between 2019 and 2023. Many thorough articles focusing on the improvement of the existing and well-known approaches were published [51,56,261]. New types of highly active [262,263] and multifunctional [205,264] nanozymes have been developed for bioanalytical applications. The performance of post-assay signal amplification significantly broadens the applicability of LFIA in practice, facilitating affordable diagnostics [8].

### 4.2. Discrepancy in LOD-Reduction Values

**Figure 12:**
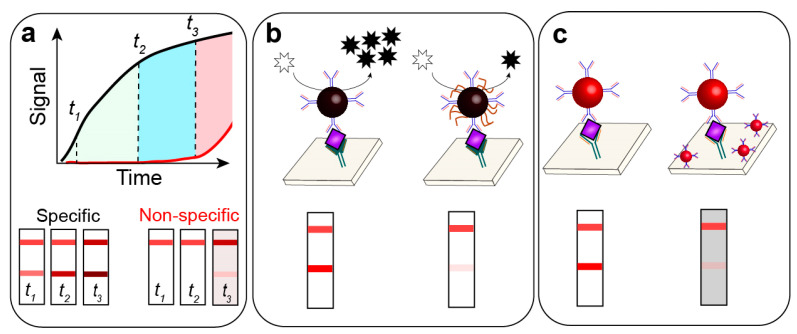
Factors causing a discrepancy in LOD reduction. (**a**) Non-optimized time of enhancement. Development of specific (black curve) and non-specific (red curve) coloration during time. (**b**) Heterogeneity of nanozyme’s catalytic activity. (**c**) Non-specific adsorption of nanolabels causing background.

Evaluating the results of LOD reduction (Figure 11a,b), one may notice that for the same approach (e.g., gold enhancement), the authors report LOD reduction from 2-times to three-to-four orders of magnitude. The non-optimal performance of some enhancements can explain such a dramatic difference in LOD reduction. As a result, the enhancement approach does not facilitate the maximal possible LOD decrease. We suggest several reasons explaining this discrepancy in LOD reduction (Figure 12) and suggest ways to overcome the limitations and implement the enhancement approaches’ full potential.

Factor 1: Non-optimized time of signal enhancement (Figure 12a). The time of the amplification is selected experimentally. The amplification time that facilitates the highest increase of the “specific” signal (test strip after LFIA of sample with antigen, Figure 12a, black curve) while keeping the “non-specific” signal (test strip after LFIA of sample without antigen, Figure 12a, red curve) minimal is considered optimal (Figure 12a, area between *t*_2_ and *t*_3_ shown in blue). Often, the developers tend to reduce the amplification time to keep LFIA in the POC paradigm. In this case (Figure 12a, area between *t*_1_ and *t*_2_ shown in green), the full potential of the enhancement is not realized, and the lower reduction of LOD is obtained. On the contrary, if the enhancement reaction is performed for too long, the increase in the “non-specific” signal limits the benefits of the “specific” signal enhancement (Figure 12a, area after *t*_3_ shown in red). The time of the signal enhancement can vary significantly (Figure 11c,d). Often, the developers do not optimize the reaction time and use the time reported in the literature. As a result, the full potential of the approach for LOD reduction may remain unrealized. Thus, the selection of optimal time (finding the “blue” area, Figure 12a) should be performed for each new development. The effect of temperature during the enhancement reaction is rarely studied, while this parameter affects the LOD reduction, background staining, and optimal enhancement time [40].

Factor 2: Non-equal chemical conditions while performing chemical reactions of signal amplifications (Figure 12b). In all considered amplifications, the functionality of the nanoparticles’ surface plays a critical role. The signal enhancement is performed after the completion of conventional LFIA, and the particles in the membrane cannot be considered functionally homogenous. Adsorbed on the surface of nanoparticles receptors and matrix components, the charge of particles and density of distribution on the membranes will significantly affect the reactivity of the particles. For LFIA amplification, it means that nanoparticles in the test zone will survive various chemical transformations. As a result, initially homogeneous particles (size, shape, charge) will transform into heterogeneously enlarged nanoparticles with different optical properties. This observation is applicable for gold, silver, and copper enhancement. As a result, accurate comparison, even within one approach, can be challenging.

The presence of adsorbed protein molecules on the GNP surface slows down the gold enhancement reactions and results in the formation of larger, non-spherical particles because of the non-uniform diffusion of reagents to the surface of the nanoparticles [37,38]. For nanozyme-assisted amplification, the surface blockage causes the reduction of catalytic activities [203,204]. Thus, nanozyme particles in the test zone cannot be considered the particles with the same catalytic properties (as accepted for bare particles) (Figure 12b). For the enzyme-assisted amplification, the activity of enzymes after binding to nanoparticles is rarely characterized. After immobilizing, part of the enzyme molecules loses catalytic activity. Thus, nanoparticles with enzyme labels are catalytically heterogeneous. Therefore, the LOD reduction with enzymes will be determined by its functionality and matrix effect that may contain inhibitors (e.g., phosphate anions for alkaline phosphatase) and endogenous enzymes causing background. In conclusion, the heterogeneity of functional properties of nanoparticles results in unequal conditions for the performance of signal amplification reactions. Such heterogeneity could be a source of the challenges in finding the optimum amplification time. Further progress in the unification of chemical amplification methods lies in the fundamental studies of nanoparticle synthesis, functional activity, and bioconjugation.

Factor 3: Various impacts of background signal. Background signal arises either from the non-specific binding of immunoreagents or the interfering activity of matrix components. The non-specific binding of immunoreagents arises from the cross-reactivity of bioreceptors and the high prevalence of non-target components in matrices (Figure 12c). Background from bioreceptors’ cross-reactivity can be diminished by using highly specific binders. The impact of the high prevalence of non-target components is a more common problem, as the target molecules are presented in the micro-to-pico molar range on the background of two-to-four orders of magnitude more concentrated non-target components. As the conventional LFIA does not have additional washing steps to exclude non-target components, the issue of non-specific binding is especially challenging. As a result of such binding, nanoparticles are non-specifically accumulated in the test zone. Usually, for conventional LFIA, the concentration of nanoparticles is low enough to generate a non-detectable signal. After chemical enhancement, both specific and non-specific signals are increased. The increased non-specific signal limits the LOD value. Unfortunately, there is no signal solution for this problem. Various blocking agents (BSA, skimmed milk, gelatin, DNA from salmon sperm, PEGs) and detergents (Tween, Triton, SDS) are used in buffer and membrane pretreatment. Elimination of background remains the issue of optimization for each LFIA development. Some matrix components may interfere with the performance of chemical amplification reactions. For example, natural peroxidases in biosamples (raw food components, plant extracts) will interfere with peroxidase-mimicking nanozymes and HRP-assisted signal amplification. Natural peroxidases catalyze the oxidation of the substrate, resulting in the significant increase of background coloration (signal) that may completely mask the “target” coloration from the nanozyme/enzyme label. In conclusion, the observed discrepancy of LOD reduction is a complex problem that arises from the functional heterogeneity of nanoparticles, unequal conditions during chemical enhancement, and matrix effects.

### 4.3. Proposal of Additional Studies in LFIA with Post-Assay Enhancement

We argue that in addition to the fundamental studies and development of new enhancement approaches, four types of comparative research (a–d) are important for the accurate evaluation of enhancement approaches.

(a) Comparison of various enhancement approaches performed with the same antigen and immunoreagents.

Most articles report the development of only one enhancement approach applied for one antigen. This format is applicable for demonstrating the benefits of the particular enhanced LFIA over conventional assay. However, the lack of articles comparing the enhancement approaches hinders the evaluation of their performance. It is necessary to rank the enhancement approaches in terms of LOD reduction, time of performance, applicability in various matrices, etc. For example, many labels have been studied in ELISA [150,265,266,267] (enzymes such as HRP, ALP, β-galactosidase, acetylcholinesterase, catalase, penicillinase). However, at the moment, the set of labels has narrowed down to HRP and partially ALP, while the other enzymes could not stand the competition due to low sensitivity, poor stability, high price, safety issues of substrates, etc. Similarly, enhancement approaches for LFIA should be evaluated and ranked. To do this, the comparison of various enhancement approaches performed by the same developer for one target using the same immunoreagents is required.

**Figure 13:**
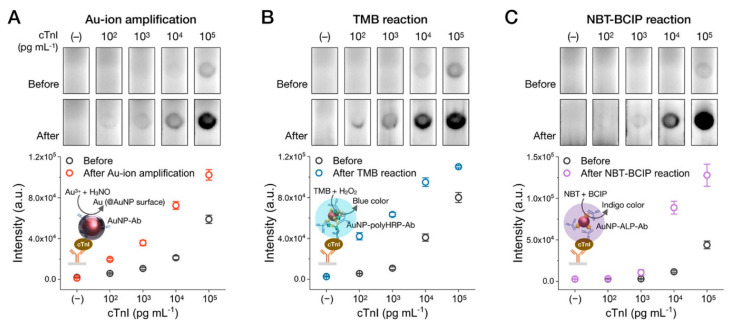
Comparison of three enhancement approaches for LFIA of human cardiac troponin I using the same immunoreagents. (**A**) Gold enhancement. (**B**) HRP-catalyzed signal enhancement. (**C**) ALP-catalyzed signal enhancement [268].

Currently, very few articles report the comparison of enhancement approaches. Phan and coauthors compared the performance of silver and copper [92] and gold and copper [97] enhancement approaches for dot blot paper assay. The authors report higher LOD reduction for copper enhancement, comparable time for copper and silver enhancement, and a more rapid procedure for gold enhancement. Tian and coauthors compared silver and copper enhancement using commercial and self-made test strips [99]. Han and coauthors [268] compared the performance of gold enhancement (Figure 13A), HRP (Figure 13B), and ALP (Figure 13C) enhancement for colorimetric signal amplification in LFIA. The authors did not report LOD values for all three enhancement approaches. However, as can be seen from calibration plots, HRP enhancement facilitates the highest enhancement of colorimetric signal at low concentrations. ALP enhancement showed the lowest signal amplification among all three studied.

In theory, multiple post-assay enhancement approaches can be combined and performed consequently, although very few papers have studied such combinations experimentally. Panferov and coauthors have shown that combination of silver enhancement (Figure 1b) and consequent galvanic replacement of silver with HAuCl_4_ (Figure 1c) facilitates 61-times-lower LOD compared with GNPs and 7-times-lower LOD compared with LFIA after silver enhancement [40]. Fu and coauthors [230] consequently performed gold enlargement and nanozyme amplification. The LOD values of *E. coli* O157:H7 were reduced 40 times compared with GNP and 10 times compared with gold enhancement.

Important to note is that both papers were not dedicated to combining multiple enhancement approaches. Panferov and coauthors [40] studied the various approaches to enlarging GNPs. Fu and coauthors [230] proposed an approach to restore the catalytic surface of nanozyme in situ. It remains unclear if the combination of enhancement approaches has an additive effect on LOD reduction. However, the increase in time and cost of reagents will be additive. As a result, more complex LFIA for multiple post-assay enhancements raises questions about the practicality of such a combination.

(b) Comparison of one enhancement approach for various antigens in different matrices performed by the same group.

Most articles use one antigen to demonstrate the benefits of the enhancements approach. Comparison of LOD values for one enhancement strategy for various antigens in different matrices is important, as it demonstrates the impact of matrix components on enhancement. Although some papers report the use of enhancement approach for LFIA of various antigens (for example, prostate-specific antigen and carcinoembryonic antigen [182]; *Escherichia coli* O157:H7, *Staphylococcus aureus*, *Salmonella typhimurium*, and *Bacillus cereus* [241]; myoglobin, cardiac troponin I, creatine kinase MB isoenzyme [177]), the compared antigens were in the same matrix. Comparison of LODs for such cases does not allow the evaluation of the impact of matrix components on signal enhancement reaction.

(c) Quantitative comparison of research (a) and/or (b) performed with an interval of time.

Such a comparison must be performed using stored and freshly prepared stock solutions of enhancing reagents. This experiment focuses on evaluating the stability of enhancing reagents and immunoreagents during storage. While the high stability of antibodies and GNP conjugates are often reported in the articles (and confirmed by the number of commercially available products), the stability of enhancing reagents usually remains undiscussed. The authors usually report using freshly prepared enhancing reagents to avoid the negative effect of storage. For further application in commercial products, one needs to measure the stability of enhancing reagents and find solutions to increase their stability if required.

(d) Comparison of the enhancement approach for one target using the same reagents performed by different groups.

With the growing concerns about reproducibility in science, it is crucial to evaluate the reproducibility of enhancement approaches. Similar research has been performed for ELISA [269], PCR [270], and biosensors [271]. The comparison experiment is focused on the study of the reproducibility of LOD values, time of amplification, the amplitude of the enhanced signal, background, linear range, etc., for each approach performed by independent laboratories.

## 5. Development and Validation of Enhanced LFIA

Highly sensitive assays (down to a single molecule) are a trend in bioanalytical chemistry [272], although one may question the practical necessity of such ultra-sensitive assays. Indeed, the set of targets that need to be detected at the level of single molecules is limited to some pathogens. The predominant set of targets needs to be detected in the pico-to-nano molar range, i.e., at least six orders of magnitude higher than the single molecule level. However, ultra-sensitive assays for the detection of target analytes are in demand because they facilitate the detection of oncoming health threats before symptoms arise and allow doctors to take preventive measurements. Also, ultrasensitive detection allows the analyst to dilute the matrix with buffer for the reduction matrix effect. Finally, even if the ultrasensitive detection is not practically needed for the particular target (e.g., human chorionic gonadotropin), the developments show a proof of concept that can be further applied to required targets [11].

**Figure 14:**
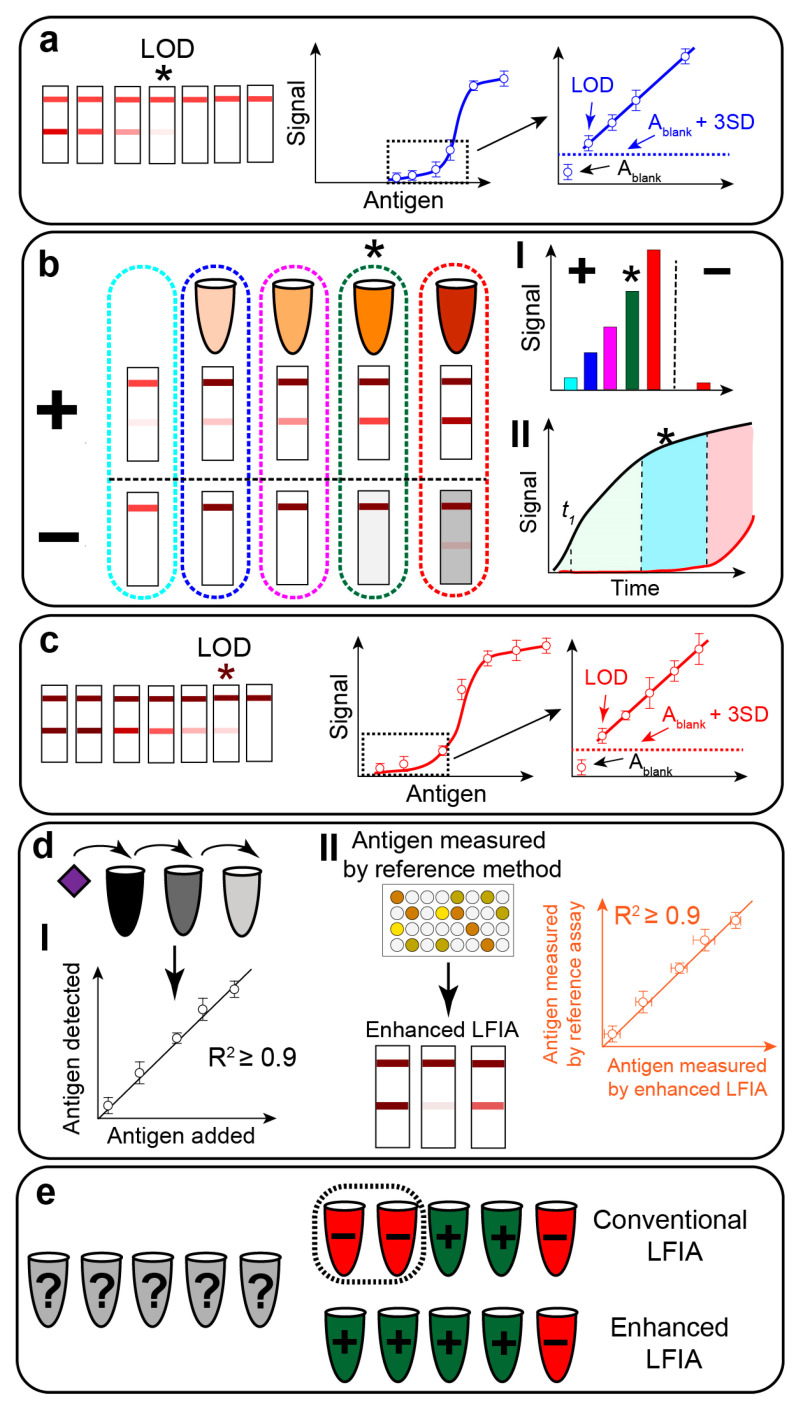
Workflow for the development of enhanced LFIA. (**a**) Determination of LOD of conventional (before enhancement) LFIA. (**b**) Optimization of the enhancement stage. Various concentrations of components in enhancing solution are used for signal amplification in positive (+) and negative (−) LFIA. Optimal concentration facilitates the highest amplification for (+) while keeping the signal for (−) minimal (I). The optimal concentration is shown with an asterisk. Optimal enhancement time is selected using an optimized enhancing solution. Optimal enhancement time facilitates maximal signal amplification for (+) while keeping signal for (−) minimal (II). The optimal time is shown as the blue area and marked with an asterisk. (**c**) Determination of LOD of enhanced LFIA. (**d**) Determination of the accuracy of enhanced LFIA. Matrix is spiked with the known concentration of the antigen. Afterward, the concentration is determined by enhanced LFIA. Correlation between spiked and measured concentrations is characterized quantitatively with R^2^ coefficient (I). The concentration of antigen is measured in real samples with reference methods and enhanced LFIA. Correlation between concentrations measured by the reference method and enhanced LFIA is characterized quantitatively with R^2^ coefficient (II). (**e**) Detection of antigen is performed in real samples with conventional and enhanced LFIA. Qualitative and/or quantitative characterization of real samples is performed by the reference method. The number of true positive and negative, false positive and negative, and specificity and sensitivity are calculated for conventional and enhanced LFIA.

However, such a race for ultra-low LOD values often sacrifices proper approbation and validation of the enhanced LFIA. We suggest a workflow of experiments for characterizing newly developed enhanced LFIA (Figure 14). The workflow starts with the quantification of LOD value for conventional LFIA (Figure 14a), optimization of post-assay enhancement (Figure 14b), quantification of LOD value for enhanced LFIA (Figure 14c), measuring accuracy of enhanced LFIA (Figure 14d), and validation of enhanced LFIA using real samples (Figure 14e). We argue that this is a required minimum for reporting enhanced LFIA. Note that the proposed workflow focuses only on post-assay chemical enhancement. The methodological recommendations about the development of conventional LFIA (i.e., all steps before quantification of LOD, Figure 14a) can be found in tutorials [273,274,275].

1. Determination of the LOD value before enhancement for conventional LFIA in the matrix (Figure 14a). LOD is a quantitative value that is determined as the target concentration that facilitates the coloration higher than the coloration of the blank probe (A_blank_) plus three standard deviations of blank (SD_blank_). From our experience, LFIA with even minimal A_blank_ is poorly applicable for post-assay signal enhancement, as it results in a high background. Often visual LOD of LFIA is reported. Visual LOD is determined as the lowest analyte concentration facilitating detectable by bare-eye coloration of the test zone. Although visual evaluation of test strips remains common in practice (e.g., pregnancy test screening, SARS-CoV-2 antigen rapid tests), for scientific developments, LOD quantification is recommended. Visual LOD determination will be subjective and depend on the visual acuity, brightness of environment light, matrix coloration, etc. Additional optimization for elimination A_blank_ before proceeding further with post-assay chemical enhancement. Determining LOD in the matrix, not the buffer, is important, as matrix components may cause higher A_blank_. To perform the first stage, the matrix is spiked with the known concentrations of the target. Each concentration is measured at least in three repeats. As a result of this step, the developer must have the LOD value of LFIA in the matrix.
2. Optimization of chemical enhancement (Figure 14b). At least two parameters need to be optimized—concentrations of enhancing reagents (Figure 14b(I)) and reaction time (Figure 14b(II)). For the particular enhancement, other variables (e.g., pH of the solution, presence of stabilized agents, method of signal enhancing solution introduction, temperature, etc.) need to be optimized, but here we focus on general parameters. Optimization of the signal enhancing reagents can be performed within the recommended ranges of concentrations and reaction time (Figure 11c,d). For these experiments, at least two samples are used. The first sample should contain the target in a concentration close to the LOD value. The second sample should be an unspiked matrix (blank probe). The goal of the optimization is to find the conditions that facilitate the highest signal enhancement for the first sample (marked with an asterisk) while keeping no signal for the second sample.
3. Determination of the LOD value after enhancement (Figure 14c). The LOD value for the optimized procedure (second stage) is determined as described in the first stage. Importantly, the developers should use A_blank_ and SD_blank_ values for enhanced LFIA, not for conventional, otherwise, the LOD value will be miscalculated. LOD reduction is calculated by dividing LOD values before and after enhancement. It is crucial to understand which LOD values can be compared for reporting enhancement effect. Often authors compare enhanced LFIA with “conventional” GNP-based LFIA, commercial test systems, or even LFIA published in other publications. We claim that LOD values should be compared exclusively between LFIA assembled with the same membranes and immunoreagents. Thus, all comparisons with literature and commercial test systems are eligible to show the superior analytical performance of developed LFIA but not the enhancement strategy. For reporting the benefits of the enhancement approach, it is necessary to compare LOD values within a single study. Also, one needs to understand the principles of signal amplification to compare LOD values before and after accurately. For example, for gold/silver/copper enhancement, one needs to compare the LOD values of LFIA with GNPs before and after the reduction of corresponding salts. Ideally, the same test strips should be used. For nanozyme signal amplification, one needs to compare LOD values before and after the addition of the substrate. The comparison of LOD values for nanozymes after catalysis with GNP will not be accurate because nanozymes as the optical label (before catalysis) may have different LOD values compared to GNPs. Thus, the developers need to clearly understand the principle of signal amplification and only compare LOD values before and after amplification within one strategy. To perform this stage, the developer spikes the matrix with the known concentration of the target and prepares the number of consequent dilutions (titration) as described for stage one. Ideally, calibration plots for LFIA before (stage 1) and after enhancement (stage 3) and LOD calculations should be performed in parallel using the same stock solution. Such performance in parallel will reduce the impact of determinate and indeterminate errors.
4. Determination of the accuracy of LFIA before and after enhancement using the spiked matrix (Figure 14d). Using the calibration plots (stages 1 and 3 for LFIAs before and after enhancement, respectively), the developer determines the linear range based on correlation coefficient (R^2^ ≥ 0.9×) [276]. After that, multiple artificially spiked matrix samples are prepared with the target concentration within the linear range. Then, conventional and enhanced LFIAs are used to quantify the target concentrations. The results can be shown as added–detected (in percent) or in a graphical way (added concentration vs. detected concentration). Values close to 100% in added–detected or R^2^ ≥ 0.99 for a graphical representation are expected for accurate LFIA.
5. Validation of conventional and enhanced LFIAs using real samples (Figure 14e). This stage aims to confirm the practical benefits of the enhanced LFIA over conventional LFIA. As the practical benefits, we understand the ability of enhanced LFIA to detect low-positive samples, while conventional LFIA report false negative results due to insufficiently low LOD value. To perform this stage, real samples containing the target in a wide range of concentrations (true positive) and without the target (true negative) are used. The concentration of the target (or at least the qualitative results) should be confirmed by an independent method (PCR, ELISA). The results of the independent method are considered the reference—i.e., true positive (presence of the target) and true negative (absence of the target). Ideally, the developer should be blinded and perform LFIA without knowing the results of the independent method. The qualitative (number of true positive/negative and number of false positive/negative) and quantitative (concentration of the target) results of conventional and enhanced LFIAs are compared with the independent method. One expects a higher number of true positives (lower number of false negatives) for enhanced LFIA compared with conventional LFIA because of lower LOD. Also, the number of false positives should be the same, meaning that the enhancement procedure does not sacrifice the specificity of the assay.

Similarly to the article of Bustin and coauthors [277], we suggest the minimum information that needs to be disclosed in an article reporting post-assay LFIA enhancement. The report should contain detailed information about the following:

Reagents—supplier, purity, reference/catalog number. If any purification or derivatization procedures were performed, they should be reported.

Enhancement procedure—the authors should report a detailed enhancement protocol that includes information about the concentration, volume and type of buffer of enhancing reagents, storage conditions of enhancing reagents, the temperature during enhancement, the necessity of washing before and after enhancement, known inhibitors of enhancement reaction, and time of enhancement reaction. The detailed procedure for matrix preparation should be reported. In addition to this list of the minimum required information, if anything else is important for the reproducibility of the enhancement approach (e.g., the order of mixing of enhancing reagents), it should be reported.

## 6. Conclusions and Perspectives

Lateral flow immunoassay has been come a long way since its introduction as a rapid pregnancy screening tool. Today, LFIA rapid tests are used in medicine, veterinary, food control, and environmental monitoring. The COVID-19 pandemic sharply emphasized the need for accessible and affordable screening tools [8]. Billions of LFIA tests were performed during the pandemic, making LFIA an indispensable instrument in the test-trace-isolate-quarantine strategy [278]. As a self-testing tool, LFIA demonstrates good acceptability by patients/end-users [279,280], which makes it perfectly suitable for a wide scope of applications. Increasing the sensitivity of LFIA is an inevitable requirement for further broadening its application areas.

The prospects for post-assay chemical enhancements lie in the development of new, more effective, and affordable methods. All bioanalytical methods include a signal amplification step—polymerases multiply the number of nucleic acids in PCR, and enzymes catalyze the reaction of product to subtract transformation in ELISA and biosensors. All these methods borrow “biological” amplification—approaches used by cells. Post-assay amplification in LFIA steps out of the line, as, in addition to biological amplification approaches (enzyme amplification), it mainly relies on “non-biological” amplification (silver, gold, copper enhancement, crosslinking of nanoparticles, nanozymes). Further developments of post-assay enhancement approaches may follow both ways—borrowing “biological” amplification or applying “artificial” amplification principles.

Considering “biological” amplification, PCR-amplification facilitates exponential amplification of the number of detectable targets. Various types of PCR-like amplification are performed before LFIA, while the test strips are used only to detect the amplified target sequences [281]. However, PCR-like amplification has not been utilized for post-assay signal enhancement on the test strip in situ. Recent developments demonstrated the performance of PCR-like amplification of nucleic acid in the integrated test strip holder [282]. Also, Posner’s group developed multiple assays with isothermal nucleic acid amplification and consequent purification on the paper device [283,284,285]. Utilizing the principle of immuno-PCR [286], where nucleic acids act as an amplifiable tag, this approach can be used for highly sensitive LFIA of various targets. The further development of post-assay PCR amplification (e.g., use of isothermal polymerases) may provide a new, highly efficient signal enhancement approach.

Considering “non-biological” amplification, progress is related to the improvement of the existing enhancement approaches and the development of new ones. For example, the improvement of the gold, silver, and copper enhancement approaches is related to the development of low-background protocols and stable, ready-to-use enhancing solutions. Such efforts require a more detailed fundamental study of metal overgrowth, kinetic of nanoparticles growth, colloidal stability of nanoparticles, etc. Considering the development of new approaches, developments in related areas of signal amplification can be adapted for LFIA, e.g., radical polymerization [287].

Also, the further progress of post-assay enhanced LFIA is undoubtedly related to the development of more user-friendly amplification protocols and their application in commercial products. The developers may even sacrifice the sensitivity to get a more user-friendly test system.

## Figures and Tables

**Table 1:** Comparison of the post-assay chemical enhancement strategies.

Enhancement Strategy	Range of LOD Reduction, Times	Duration of the Enhancement Stage, Min	Comments
Copper enhancement	7.5–40,000	3–15	High stability of enhancement reagents. No inhibitors were reported. Results readout should be done after completion of the enhancement procedure. The approach has been characterized in a small number of studies.
Silver enhancement	2–1000	2–30	Low shelf life of enhancement reagents. Light-sensitive reagents. Enhancement is inhibited by chloride and phosphate ions. Enhancement solutions are available as commercial products. Silver enhancement reaction can be terminated for delayed result readout.
Gold enhancement	1.68–1024	1–10	High stability of enhancement reagents. No inhibitors of the enhancement reaction were reported. Enhancement is faster compared to other strategies. Results readout should be done after completion of the enhancement procedure. Enhancement solutions are available as commercial products.
Enzyme enhancement	4–1000	0.5–25	High stability of enhancement reagents in a dry form. The stability of substrate solutions should be evaluated. Matrix components may inhibit the amplification reactions. Selection of the enzyme label should be done based on the endogenous enzyme activity of the matrix. Results readout should be done after completion of the enhancement procedure. Enhancement solutions are available as commercial products.
Nanozyme enhancement	1.7–50,000	0.5–30	High stability of enhancement reagents in a dry form. The stability of substrate solutions should be evaluated. Optimal concentrations of the substrates should be evaluated. Matrix components may inhibit the amplification reactions. Results readout should be done after completion of the enhancement procedure. Enhancement solutions are available as commercial products.

## Data Availability

Not applicable.

## Supplementary Materials

The following supporting information can be downloaded at: https://www.mdpi.com/article/10.3390/bios13090866/s1, Table S1: Turnover numbers for HRP and nanozymes; Figure S1: Characteristics of nanozymes as the catalytic labels; Table S2: LOD values for various enhancement methods; Table S3: Time of various enhancement methods.

## References

[1] Di Nardo F., Chiarello M., Cavalera S., Baggiani C., Anfossi L. (2021). Ten Years of Lateral Flow Immunoassay Technique Applications: Trends, Challenges and Future Perspectives. Sensors.

[2] Wang Z., Zhao J., Xu X., Guo L., Xu L., Sun M., Hu S., Kuang H., Xu C., Li A. (2022). An Overview for the Nanoparticles-Based Quantitative Lateral Flow Assay. Small Methods.

[3] Huang Y., Xu T., Wang W., Wen Y., Li K., Qian L., Zhang X., Liu G. (2020). Lateral Flow Biosensors Based on the Use of Micro- and Nanomaterials: A Review on Recent Developments. Microchim. Acta.

[4] Urusov A.E., Zherdev A.V., Dzantiev B.B. (2019). Towards Lateral Flow Quantitative Assays: Detection Approaches. Biosensors.

[5] Sena-Torralba A., Álvarez-Diduk R., Parolo C., Piper A., Merkoçi A. (2022). Toward Next Generation Lateral Flow Assays: Integration of Nanomaterials. Chem. Rev..

[6] Hristov D., Rodriguez-Quijada C., Gomez-Marquez J., Hamad-Schifferli K. (2019). Designing Paper-Based Immunoassays for Biomedical Applications. Sensors.

[7] Liu Y., Zhan L., Qin Z., Sackrison J., Bischof J.C. (2021). Ultrasensitive and Highly Specific Lateral Flow Assays for Point-of-Care Diagnosis. ACS Nano.

[8] Budd J., Miller B.S., Weckman N.E., Cherkaoui D., Huang D., Decruz A.T., Fongwen N., Han G.-R., Broto M., Estcourt C.S. (2023). Lateral Flow Test Engineering and Lessons Learned from COVID-19. Nat. Rev. Bioeng..

[9] Dzantiev B.B., Byzova N.A., Urusov A.E., Zherdev A.V. (2014). Immunochromatographic Methods in Food Analysis. TrAC Trends Anal. Chem..

[10] Bishop J.D., Hsieh H.V., Gasperino D.J., Weigl B.H. (2019). Sensitivity Enhancement in Lateral Flow Assays: A Systems Perspective. Lab Chip.

[11] Zherdev A.V., Dzantiev B.B. (2022). Detection Limits of Immunoanalytical Systems: Limiting Factors and Methods of Reduction. J. Anal. Chem..

[12] Anfossi L., Di Nardo F., Cavalera S., Giovannoli C., Baggiani C. (2018). Multiplex Lateral Flow Immunoassay: An Overview of Strategies towards High-Throughput Point-of-Need Testing. Biosensors.

[13] Zherdev A.V., Dzantiev B.B. (2018). Ways to Reach Lower Detection Limits of Lateral Flow Immunoassays. Rapid Test—Advances in Design, Format and Diagnostic Applications.

[14] Foote J., Eisen H.N. (1995). Kinetic and Affinity Limits on Antibodies Produced during Immune Responses. Proc. Natl. Acad. Sci. USA.

[15] Batista F.D., Neuberger M.S. (1998). Affinity Dependence of the B Cell Response to Antigen: A Threshold, a Ceiling, and the Importance of Off-Rate. Immunity.

[16] Razo S., Panferov V., Safenkova I., Varitsev Y., Zherdev A., Pakina E., Dzantiev B. (2018). How to Improve Sensitivity of Sandwich Lateral Flow Immunoassay for Corpuscular Antigens on the Example of Potato Virus Y?. Sensors.

[17] Rivas L., Medina-Sánchez M., de la Escosura-Muñiz A., Merkoçi A. (2014). Improving Sensitivity of Gold Nanoparticle-Based Lateral Flow Assays by Using Wax-Printed Pillars as Delay Barriers of Microfluidics. Lab Chip.

[18] Shirshahi V., Liu G. (2021). Enhancing the Analytical Performance of Paper Lateral Flow Assays: From Chemistry to Engineering. TrAC Trends Anal. Chem..

[19] Kim K., Kashefi-Kheyrabadi L., Joung Y., Kim K., Dang H., Chavan S.G., Lee M.-H., Choo J. (2021). Recent Advances in Sensitive Surface-Enhanced Raman Scattering-Based Lateral Flow Assay Platforms for Point-of-Care Diagnostics of Infectious Diseases. Sens. Actuators B Chem..

[20] Gong X., Cai J., Zhang B., Zhao Q., Piao J., Peng W., Gao W., Zhou D., Zhao M., Chang J. (2017). A Review of Fluorescent Signal-Based Lateral Flow Immunochromatographic Strips. J. Mater. Chem. B.

[21] Moyano A., Serrano-Pertierra E., Salvador M., Martínez-García J.C., Rivas M., Blanco-López M.C. (2020). Magnetic Lateral Flow Immunoassays. Diagnostics.

[22] Qin Z., Chan W.C.W., Boulware D.R., Akkin T., Butler E.K., Bischof J.C. (2012). Significantly Improved Analytical Sensitivity of Lateral Flow Immunoassays by Using Thermal Contrast. Angew. Chem. Int. Ed..

[23] Khlebtsov B.N., Tumskiy R.S., Burov A.M., Pylaev T.E., Khlebtsov N.G. (2019). Quantifying the Numbers of Gold Nanoparticles in the Test Zone of Lateral Flow Immunoassay Strips. ACS Appl. Nano Mater..

[24] Deng Y., Jiang H., Li X., Lv X. (2021). Recent Advances in Sensitivity Enhancement for Lateral Flow Assay. Microchim. Acta.

[25] Panferov V.G., Safenkova I.V., Zherdev A.V., Dzantiev B.B. (2021). Methods for Increasing Sensitivity of Immunochromatographic Test Systems with Colorimetric Detection (Review). Appl. Biochem. Microbiol..

[26] Khlebtsov N.G. (2008). Determination of Size and Concentration of Gold Nanoparticles from Extinction Spectra. Anal. Chem..

[27] Zhan L., Guo S., Song F., Gong Y., Xu F., Boulware D.R., McAlpine M.C., Chan W.C.W., Bischof J.C. (2017). The Role of Nanoparticle Design in Determining Analytical Performance of Lateral Flow Immunoassays. Nano Lett..

[28] Dolinnyi A.I. (2017). Extinction Coefficients of Gold Nanoparticles and Their Dimers. Dependence of Optical Factor on Particle Size. Colloid J..

[29] Li J., Duan H., Xu P., Huang X., Xiong Y. (2016). Effect of Different-Sized Spherical Gold Nanoparticles Grown Layer by Layer on the Sensitivity of an Immunochromatographic Assay. RSC Adv..

[30] Jain P.K., Lee K.S., El-Sayed I.H., El-Sayed M.A. (2006). Calculated Absorption and Scattering Properties of Gold Nanoparticles of Different Size, Shape, and Composition: Applications in Biological Imaging and Biomedicine. J. Phys. Chem. B.

[31] de Puig H., Tam J.O., Yen C.-W., Gehrke L., Hamad-Schifferli K. (2015). Extinction Coefficient of Gold Nanostars. J. Phys. Chem. C.

[32] Chen X., Ding L., Huang X., Xiong Y. (2022). Tailoring Noble Metal Nanoparticle Designs to Enable Sensitive Lateral Flow Immunoassay. Theranostics.

[33] Brown K.R., Lyon L.A., Fox A.P., Reiss B.D., Natan M.J. (2000). Hydroxylamine Seeding of Colloidal Au Nanoparticles. 3. Controlled Formation of Conductive Au Films. Chem. Mater..

[34] Penner R.M. (2001). Brownian Dynamics Simulations of the Growth of Metal Nanocrystal Ensembles on Electrode Surfaces in Solution: 2. The Effect of Deposition Rate on Particle Size Dispersion. J. Phys. Chem. B.

[35] Scharifker B. (1982). Theoretical and Experimental Studies of Multiple Nucleation. Electrochim. Acta.

[36] Brown K.R., Natan M.J. (1998). Hydroxylamine Seeding of Colloidal Au Nanoparticles in Solution and on Surfaces. Langmuir.

[37] Panferov V.G., Safenkova I.V., Zherdev A.V., Dzantiev B.B. (2018). Post-Assay Growth of Gold Nanoparticles as a Tool for Highly Sensitive Lateral Flow Immunoassay. Application to the Detection of Potato Virus X. Microchim. Acta.

[38] Panferov V.G., Samokhvalov A.V., Safenkova I.V., Zherdev A.V., Dzantiev B.B. (2018). Study of Growth of Bare and Protein-Modified Gold Nanoparticles in the Presence of Hydroxylamine and Tetrachloroaurate. Nanotechnol. Russ..

[39] Festag G., Steinbrück A., Csáki A., Möller R., Fritzsche W. (2007). Single Particle Studies of the Autocatalytic Metal Deposition onto Surface-Bound Gold Nanoparticles Reveal a Linear Growth. Nanotechnology.

[40] Panferov V.G., Byzova N.A., Biketov S.F., Zherdev A.V., Dzantiev B.B. (2021). Comparative Study of In Situ Techniques to Enlarge Gold Nanoparticles for Highly Sensitive Lateral Flow Immunoassay of SARS-CoV-2. Biosensors.

[41] Wang Y., Liu P., Ye Y., Hammock B.D., Zhang C. (2023). An Integrated Approach to Improve the Assay Performance of Quantum Dot-Based Lateral Flow Immunoassays by Using Silver Deposition. Microchem. J..

[42] Dias J.T., Svedberg G., Nystrand M., Andersson-Svahn H., Gantelius J. (2017). Rapid Signal Enhancement Method for Nanoprobe-Based Biosensing. Sci. Rep..

[43] Shu R., Liu S., Xu J., Wang S., Ma Y., Chen Y., Li Y., Sun J., Zhang D., Wang J. (2022). Galvanic Replacement Inspired Signal Amplification: Background-Free and Antibody-Thrift in-Situ Growth Immunochromatography. Chem. Eng. J..

[44] Xia X., Wang Y., Ruditskiy A., Xia Y. (2013). 25th Anniversary Article: Galvanic Replacement: A Simple and Versatile Route to Hollow Nanostructures with Tunable and Well-Controlled Properties. Adv. Mater..

[45] de la Rica R., Stevens M.M. (2012). Plasmonic ELISA for the Ultrasensitive Detection of Disease Biomarkers with the Naked Eye. Nat. Nanotechnol..

[46] Cao C., Li X., Lee J., Sim S.J. (2009). Homogenous Growth of Gold Nanocrystals for Quantification of PSA Protein Biomarker. Biosens. Bioelectron..

[47] Morris R.E., Ciraolo G.M., Saelinger C.B. (1991). Gold Enhancement of Gold-Labeled Probes: Gold-Intensified Staining Technique (GIST). J. Histochem. Cytochem..

[48] Zayats M., Baron R., Popov I., Willner I. (2005). Biocatalytic Growth of Au Nanoparticles: From Mechanistic Aspects to Biosensors Design. Nano Lett..

[49] Wang X., Niessner R., Knopp D. (2015). Controlled Growth of Immunogold for Amplified Optical Detection of Aflatoxin B1. Analyst.

[50] Cid-Barrio L., Ruiz Encinar J., Costa-Fernández J.M. (2020). Catalytic Gold Deposition for Ultrasensitive Optical Immunosensing of Prostate Specific Antigen. Sensors.

[51] Duan H., Ma T., Huang X., Gao B., Zheng L., Chen X., Xiong Y., Chen X. (2022). Avoiding the Self-Nucleation Interference: A PH-Regulated Gold in Situ Growth Strategy to Enable Ultrasensitive Immunochromatographic Diagnostics. Theranostics.

[52] Fan A., Cai S., Cao Z., Lau C., Lu J. (2010). Hydroxylamine-Amplified Gold Nanoparticles for the Homogeneous Detection of Sequence-Specific DNA. Analyst.

[53] Ma Z., Sui S.F. (2002). Naked-Eye Sensitive Detection of Immunoglubulin G by Enlargement of Au Nanoparticles in Vitro. Angew. Chem. Int. Ed..

[54] Habib A., Tabata M., Wu Y.G. (2005). Formation of Gold Nanoparticles by Good’s Buffers. Bull. Chem. Soc. Jpn..

[55] Panda B.R., Chattopadhyay A. (2007). Synthesis of Au Nanoparticles at “All” PH by H_2_O_2_ Reduction of HAuCl4. J. Nanosci. Nanotechnol..

[56] Presnova G.V., Zhdanov G.A., Filatova L.Y., Ulyashova M.M., Presnov D.E., Rubtsova M.Y. (2022). Improvement of Seed-Mediated Growth of Gold Nanoparticle Labels for DNA Membrane-Based Assays. Biosensors.

[57] Ruantip S., Pimpitak U., Rengpipat S., Pasomsub E., Seepiban C., Gajanandana O., Torvorapanit P., Hirankarn N., Jaru-ampornpan P., Siwamogsatham S. (2023). Self-Enhancement Lateral Flow Immunoassay for COVID-19 Diagnosis. Sens. Actuators B Chem..

[58] Thangavelu R.M., Kadirvel N., Balasubramaniam P., Viswanathan R. (2022). Ultrasensitive Nano-Gold Labelled, Duplex Lateral Flow Immunochromatographic Assay for Early Detection of Sugarcane Mosaic Viruses. Sci. Rep..

[59] Li J., Zou M., Chen Y., Xue Q., Zhang F., Li B., Wang Y., Qi X., Yang Y. (2013). Gold Immunochromatographic Strips for Enhanced Detection of Avian Influenza and Newcastle Disease Viruses. Anal. Chim. Acta.

[60] Zhao M., Yao X., Liu S., Zhang H., Wang L., Yin X., Su L., Xu B., Wang J., Lan Q. (2021). Antibiotic and Mammal IgG Based Lateral Flow Assay for Simple and Sensitive Detection of Staphylococcus Aureus. Food Chem..

[61] Bu T., Huang Q., Yan L., Huang L., Zhang M., Yang Q., Yang B., Wang J., Zhang D. (2018). Ultra Technically-Simple and Sensitive Detection for Salmonella Enteritidis by Immunochromatographic Assay Based on Gold Growth. Food Control.

[62] Wang J.Y., Chen M.H., Sheng Z.C., Liu D.F., Wu S.S., Lai W.H. (2015). Development of Colloidal Gold Immunochromatographic Signal-Amplifying System for Ultrasensitive Detection of *Escherichia coli* O157:H7 in Milk. RSC Adv..

[63] Sharma A., Tok A.I.Y., Alagappan P., Liedberg B. (2020). Gold Nanoparticle Conjugated Magnetic Beads for Extraction and Nucleation Based Signal Amplification in Lateral Flow Assaying. Sens. Actuators B Chem..

[64] Oh H.-K., Kim K., Park J., Jang H., Kim M.-G. (2021). Advanced Trap Lateral Flow Immunoassay Sensor for the Detection of Cortisol in Human Bodily Fluids. Sci. Rep..

[65] Schulz F., Homolka T., Bastús N.G., Puntes V., Weller H., Vossmeyer T. (2014). Little Adjustments Significantly Improve the Turkevich Synthesis of Gold Nanoparticles. Langmuir.

[66] Danscher G. (1981). Histochemical Demonstration of Heavy Metals—A Revised Version of the Sulphide Silver Method Suitable for Both Light and Electronmicroscopy. Histochemistry.

[67] Newman G.R., Jasani B. (1998). Silver Development in Microscopy and Bioanalysis: A New Versatile Formulation for Modern Needs. Histochem. J..

[68] Wada A., Sakoda Y., Oyamada T., Kida H. (2011). Development of a Highly Sensitive Immunochromatographic Detection Kit for H5 Influenza Virus Hemagglutinin Using Silver Amplification. J. Virol. Methods.

[69] Stierhof Y.D., Humbel B.M., Schwarz H. (1991). Suitability of Different Silver Enhancement Methods Applied to 1 Nm Colloidal Gold Particles: An Immunoelectron Microscopic Study. J. Electron Microsc. Tech..

[70] Seopsi L., Larsson L.-I., Bastholm L., Nielsen M.H. (1986). Silver-Enhanced Colloidal Gold Probes as Markers for Scanning Electron Microscopy. Histochemistry.

[71] Hacker G.W., Grimelius L., Danscher G., Bernatzky G., Muss W., Adam H. (1988). Silver Acetate Autometallography: An Alternative Enhancement Technique for Immunogold-Silver Staining (Igss) and Silver Amplification of Gold, Silver, Mercury and Zinc in Tissues. J. Histotechnol..

[72] Chevallet M., Luche S., Rabilloud T. (2006). Silver Staining of Proteins in Polyacrylamide Gels. Nat. Protoc..

[73] Danscher G., Hacker G.W., Grimelius L., Nørgaard J.O.R. (1993). Autometallographic Silver Amplification of Colloidal Gold. J. Histotechnol..

[74] Newman G.R., Jasani B. (1998). Silver Development in Microscopy and Bioanalysis: Past and Present. J. Pathol..

[75] Skutelsky E., Goyal V., Alroy J. (1987). The Use of Avidin-Gold Complex for Light Microscopic Localization of Lectin Receptors. Histochemistry.

[76] Liu R., Liu X., Tang Y., Wu L., Hou X., Lv Y. (2011). Highly Sensitive Immunoassay Based on Immunogold-Silver Amplification and Inductively Coupled Plasma Mass Spectrometric Detection. Anal. Chem..

[77] Burry R.W., Vandre D.D., Hayes D.M. (1992). Silver Enhancement of Gold Antibody Probes in Pre-Embedding Electron Microscopic Immunocytochemistry. J. Histochem. Cytochem..

[78] Panferov V.G., Safenkova I.V., Byzova N.A., Varitsev Y.A., Zherdev A.V., Dzantiev B.B. (2018). Silver-Enhanced Lateral Flow Immunoassay for Highly-Sensitive Detection of Potato Leafroll Virus. Food Agric. Immunol..

[79] Mitamura K., Shimizu H., Yamazaki M., Ichikawa M., Nagai K., Katada J., Wada A., Kawakami C., Sugaya N. (2013). Clinical Evaluation of Highly Sensitive Silver Amplification Immunochromatography Systems for Rapid Diagnosis of Influenza. J. Virol. Methods.

[80] Bazsefidpar S., Serrano-Pertierra E., Gutiérrez G., Calvo A.S., Matos M., Blanco-López M.C. (2023). Rapid and Sensitive Detection of *E. Coli* O157:H7 by Lateral Flow Immunoassay and Silver Enhancement. Microchim. Acta.

[81] Byzova N.A., Zherdev A.V., Sveshnikov P.G., Sadykhov E.G., Dzantiev B.B. (2015). Development of an Immunochromatographic Test System for the Detection of Helicobacter Pylori Antigens. Appl. Biochem. Microbiol..

[82] Yu Q., Li H., Li C., Zhang S., Shen J., Wang Z. (2015). Gold Nanoparticles-Based Lateral Flow Immunoassay with Silver Staining for Simultaneous Detection of Fumonisin B1 and Deoxynivalenol. Food Control.

[83] Xing C., Kuang H., Hao C., Liu L., Wang L., Xu C. (2014). A Silver Enhanced and Sensitive Strip Sensor for Cadmium Detection. Food Agric. Immunol..

[84] Anfossi L., Di Nardo F., Giovannoli C., Passini C., Baggiani C. (2013). Increased Sensitivity of Lateral Flow Immunoassay for Ochratoxin A through Silver Enhancement. Anal. Bioanal. Chem..

[85] Rodríguez M.O., Covián L.B., García A.C., Blanco-López M.C. (2016). Silver and Gold Enhancement Methods for Lateral Flow Immunoassays. Talanta.

[86] Apilux A., Rengpipat S., Suwanjang W., Chailapakul O. (2018). Development of Competitive Lateral Flow Immunoassay Coupled with Silver Enhancement for Simple and Sensitive Salivary Cortisol Detection. EXCLI J..

[87] Poosinuntakul N., Chanmee T., Porntadavity S., Chailapakul O., Apilux A. (2022). Silver-Enhanced Colloidal Gold Dip Strip Immunoassay Integrated with Smartphone-Based Colorimetry for Sensitive Detection of Cardiac Marker Troponin I. Sci. Rep..

[88] Shyu R.H., Shyu H.F., Liu H.W., Tang S.S. (2002). Colloidal Gold-Based Immunochromatographic Assay for Detection of Ricin. Toxicon.

[89] Broger T., Sossen B., du Toit E., Kerkhoff A.D., Schutz C., Ivanova Reipold E., Ward A., Barr D.A., Macé A., Trollip A. (2019). Novel Lipoarabinomannan Point-of-Care Tuberculosis Test for People with HIV: A Diagnostic Accuracy Study. Lancet Infect. Dis..

[90] Miyakawa K., Funabashi R., Yamaoka Y., Jeremiah S.S., Katada J., Wada A., Takei T., Shimizu K., Ozawa H., Kawakami C. (2021). SARS-CoV-2 Antigen Rapid Diagnostic Test Enhanced with Silver Amplification Technology. medRxiv.

[91] Couturier C., Wada A., Louis K., Mistretta M., Beitz B., Povogui M., Ripaux M., Mignon C., Werle B., Lugari A. (2020). Characterization and Analytical Validation of a New Antigenic Rapid Diagnostic Test for Ebola Virus Disease Detection. PLoS Negl. Trop. Dis..

[92] Phan L.M.T., Rafique R., Baek S.H., Nguyen T.P., Park K.Y., Kim E.B., Kim J.G., Park J.P., Kailasa S.K., Kim H.-J. (2018). Gold-Copper Nanoshell Dot-Blot Immunoassay for Naked-Eye Sensitive Detection of Tuberculosis Specific CFP-10 Antigen. Biosens. Bioelectron..

[93] Wei X., Liang A., Zhang S.S., Jiang Z.L. (2008). A Selective Resonance Scattering Assay for Immunoglobulin G Using Cu(II)-Ascorbic Acid-Immunonanogold Reaction. Anal. Biochem..

[94] Kim J., Park J., Lin M., Kim S., Kim G., Park S., Ko G., Nam J. (2017). Sensitive, Quantitative Naked-Eye Biodetection with Polyhedral Cu Nanoshells. Adv. Mater..

[95] Zhou Y., Chen Y., Liu Y., Fang H., Huang X., Leng Y., Liu Z., Hou L., Zhang W., Lai W. (2021). Controlled Copper in Situ Growth-Amplified Lateral Flow Sensors for Sensitive, Reliable, and Field-Deployable Infectious Disease Diagnostics. Biosens. Bioelectron..

[96] Shao Y., Xu W., Zheng Y., Wang J., Xie J., Zhu Z., Xiang X., Ye Q., Zhang Y., Xue L. (2022). Controlled PAH-Mediated Method with Enhanced Optical Properties for Simple, Stable Immunochromatographic Assays. Biosens. Bioelectron..

[97] Phan L.M.T., Kim E.B., Cheon S.A., Shim T.S., Kim H.J., Park T.J. (2020). Reliable Naked-Eye Detection of Mycobacterium Tuberculosis Antigen 85B Using Gold and Copper Nanoshell-Enhanced Immunoblotting Techniques. Sens. Actuators B Chem..

[98] Kim M.W., Park H.-J., Park C.Y., Kim J.H., Cho C.H., Phan L.M.T., Park J.P., Kailasa S.K., Lee C.-H., Park T.J. (2020). Fabrication of a Paper Strip for Facile and Rapid Detection of Bovine Viral Diarrhea Virus via Signal Enhancement by Copper Polyhedral Nanoshells. RSC Adv..

[99] Tian M., Lei L., Xie W., Yang Q., Li C.M., Liu Y. (2019). Copper Deposition-Induced Efficient Signal Amplification for Ultrasensitive Lateral Flow Immunoassay. Sens. Actuators B Chem..

[100] Peng T., Jiao X., Liang Z., Zhao H., Zhao Y., Xie J., Jiang Y., Yu X., Fang X., Dai X. (2021). Lateral Flow Immunoassay Coupled with Copper Enhancement for Rapid and Sensitive SARS-CoV-2 Nucleocapsid Protein Detection. Biosensors.

[101] Panferov V.G., Byzova N.A., Zherdev A.V., Dzantiev B.B. (2021). Peroxidase-Mimicking Nanozyme with Surface-Dispersed Pt Atoms for the Colorimetric Lateral Flow Immunoassay of C-Reactive Protein. Microchim. Acta.

[102] Bai T., Wang L., Wang M., Zhu Y., Li W., Guo Z., Zhang Y. (2022). Strategic Synthesis of Trimetallic Au@Ag–Pt Nanorattles for Ultrasensitive Colorimetric Detection in Lateral Flow Immunoassay. Biosens. Bioelectron..

[103] Bai T., Wang M., Cao M., Zhang J., Zhang K., Zhou P., Liu Z., Liu Y., Guo Z., Lu X. (2018). Functionalized Au@Ag-Au Nanoparticles as an Optical and SERS Dual Probe for Lateral Flow Sensing. Anal. Bioanal. Chem..

[104] Gao Z., Ye H., Wang Q., Kim M.J., Tang D., Xi Z., Wei Z., Shao S., Xia X. (2020). Template Regeneration in Galvanic Replacement: A Route to Highly Diverse Hollow Nanostructures. ACS Nano.

[105] Hu X., Wan J., Peng X., Zhao H., Shi D., Mai L., Yang H., Zhao Y., Yang X. (2019). Calorimetric Lateral Flow Immunoassay Detection Platform Based on the Photothermal Effect of Gold Nanocages with High Sensitivity, Specificity, and Accuracy. Int. J. Nanomed..

[106] Wang J., Zhang L., Huang Y., Dandapat A., Dai L., Zhang G., Lu X., Zhang J., Lai W., Chen T. (2017). Hollow Au-Ag Nanoparticles Labeled Immunochromatography Strip for Highly Sensitive Detection of Clenbuterol. Sci. Rep..

[107] Merkoçi F., Patarroyo J., Russo L., Piella J., Genç A., Arbiol J., Bastús N.G., Puntes V. (2020). Understanding Galvanic Replacement Reactions: The Case of Pt and Ag. Mater. Today Adv..

[108] Chen J., Wiley B., McLellan J., Xiong Y., Li Z.Y., Xia Y. (2005). Optical Properties of Pd-Ag and Pt-Ag Nanoboxes Synthesized via Galvanic Replacement Reactions. Nano Lett..

[109] Taranova N.A., Urusov A.E., Sadykhov E.G., Zherdev A.V., Dzantiev B.B. (2017). Bifunctional Gold Nanoparticles as an Agglomeration-Enhancing Tool for Highly Sensitive Lateral Flow Tests: A Case Study with Procalcitonin. Microchim. Acta.

[110] Taranova N.A., Slobodenuyk V.D., Zherdev A.V., Dzantiev B.B. (2021). Network of Gold Conjugates for Enhanced Sensitive Immunochromatographic Assays of Troponins. RSC Adv..

[111] Panferov V.G., Ivanov N.A., Mazzulli T., Brinc D., Kulasingam V., Krylov S.N. (2023). Electrophoresis-Assisted Multilayer Assembly of Nanoparticles for Sensitive Lateral Flow Immunoassay**. Angew. Chem. Int. Ed..

[112] Hendrickson O.D., Zvereva E.A., Zherdev A.V., Dzantiev B.B. (2022). Cascade-Enhanced Lateral Flow Immunoassay for Sensitive Detection of Okadaic Acid in Seawater, Fish, and Seafood. Foods.

[113] Huang X., Zhou Y., Ding L., Yu G., Leng Y., Lai W., Xiong Y., Chen X. (2019). Supramolecular Recognition-Mediated Layer-by-Layer Self-Assembled Gold Nanoparticles for Customized Sensitivity in Paper-Based Strip Nanobiosensors. Small.

[114] Sena-Torralba A., Alvarez-Diduk R., Parolo C., Torné-Morató H., Müller A., Merkoçi A. (2021). Paper-Based Electrophoretic Bioassay: Biosensing in Whole Blood Operating via Smartphone. Anal. Chem..

[115] Nanthasurasak P., Cabot J.M., See H.H., Guijt R.M., Breadmore M.C. (2017). Electrophoretic Separations on Paper: Past, Present, and Future-A Review. Anal. Chim. Acta.

[116] Panferov V.G., Ivanov N.A., Brinc D., Fabros A., Krylov S.N. (2023). Electrophoretic Assembly of Antibody–Antigen Complexes Facilitates 1000 Times Improvement in the Limit of Detection of Serological Paper-Based Assay. ACS Sens..

[117] Rao J., Lahiri J., Isaacs L., Weis R.M., Whitesides G.M. (1998). A Trivalent System from Vancomycin.D-Ala-D-Ala with Higher Affinity than Avidin.Biotin. Science.

[118] Wu P., Song J., Zuo W., Zhu J., Meng X., Yang J., Liu X., Jiang H., Zhang D., Dai J. (2023). A Universal Boronate Affinity Capture-Antibody-Independent Lateral Flow Immunoassay for Point-of-Care Glycoprotein Detection. Talanta.

[119] Javani A., Javadi-Zarnaghi F., Rasaee M.J. (2017). A Multiplex Protein-Free Lateral Flow Assay for Detection of MicroRNAs Based on Unmodified Molecular Beacons. Anal. Biochem..

[120] You Q., Zhang X., Wu F.-G., Chen Y. (2019). Colorimetric and Test Stripe-Based Assay of Bacteria by Using Vancomycin-Modified Gold Nanoparticles. Sens. Actuators B Chem..

[121] Deev S.M., Lebedenko E.N. (2009). Antibody Engineering: Molecular Constructor on the Basis of Barnase-Barstar Module. Russ. J. Bioorganic Chem..

[122] Favalli N., Bassi G., Pellegrino C., Millul J., De Luca R., Cazzamalli S., Yang S., Trenner A., Mozaffari N.L., Myburgh R. (2021). Stereo- and Regiodefined DNA-Encoded Chemical Libraries Enable Efficient Tumour-Targeting Applications. Nat. Chem..

[123] Calabria D., Calabretta M.M., Zangheri M., Marchegiani E., Trozzi I., Guardigli M., Michelini E., Di Nardo F., Anfossi L., Baggiani C. (2021). Recent Advancements in Enzyme-Based Lateral Flow Immunoassays. Sensors.

[124] Jiang B., Duan D., Gao L., Zhou M., Fan K., Tang Y., Xi J., Bi Y., Tong Z., Gao G.F. (2018). Standardized Assays for Determining the Catalytic Activity and Kinetics of Peroxidase-like Nanozymes. Nat. Protoc..

[125] Han G.-R., Ki H., Kim M.-G. (2020). Automated, Universal, and Mass-Producible Paper-Based Lateral Flow Biosensing Platform for High-Performance Point-of-Care Testing. ACS Appl. Mater. Interfaces.

[126] Zandieh M., Liu J. (2021). Nanozyme Catalytic Turnover and Self-Limited Reactions. ACS Nano.

[127] Zuk R.F., Ginsberg V.K., Houts T., Rabble J., Merrick H., Ullman E.F., Fischer M.M., Chung Slzto C., Stiso S.N., Utman D.J. (1985). Enzyme Immunochromatography—A Quantitative Immunoassay Requinng No Instrumentation. Clin. Chem..

[128] Zhang C., Zhang Y., Wang S. (2006). Development of Multianalyte Flow-through and Lateral-Flow Assays Using Gold Particles and Horseradish Peroxidase as Tracers for the Rapid Determination of Carbaryl and Endosulfan in Agricultural Products. J. Agric. Food Chem..

[129] Kim H.-S., Ko H., Kang M.-J., Pyun J.-C. (2010). Highly Sensitive Rapid Test with Chemiluminescent Signal Bands. BioChip J..

[130] Cho I.-H., Irudayaraj J. (2013). Lateral-Flow Enzyme Immunoconcentration for Rapid Detection of Listeria Monocytogenes. Anal. Bioanal. Chem..

[131] Watanabe H., Satake A., Kido Y., Tsuji A. (2002). Monoclonal-Based Enzyme-Linked Immunosorbent Assay and Immunochromatographic Rapid Assay for Dihydrostreptomycin in Milk. Anal. Chim. Acta.

[132] Panferov V.G., Safenkova I.V., Varitsev Y.A., Zherdev A.V., Dzantiev B.B. (2018). Enhancement of Lateral Flow Immunoassay by Alkaline Phosphatase: A Simple and Highly Sensitive Test for Potato Virus X. Microchim. Acta.

[133] Ono T., Kawamura M., Arao S., Nariuchi H. (2003). A Highly Sensitive Quantitative Immunochromatography Assay for Antigen-Specific IgE. J. Immunol. Methods.

[134] Kim H.T., Jin E., Lee M.H. (2021). Portable Chemiluminescence-Based Lateral Flow Assay Platform for the Detection of Cortisol in Human Serum. Biosensors.

[135] He Y., Zhang S., Zhang X., Baloda M., Gurung A.S., Xu H., Zhang X., Liu G. (2011). Ultrasensitive Nucleic Acid Biosensor Based on Enzyme-Gold Nanoparticle Dual Label and Lateral Flow Strip Biosensor. Biosens. Bioelectron..

[136] Cho J.-H., Paek E.-H., Cho I.-H., Paek S.-H. (2005). An Enzyme Immunoanalytical System Based on Sequential Cross-Flow Chromatography. Anal. Chem..

[137] Samsonova J.V., Safronova V.A., Osipov A.P. (2015). Pretreatment-Free Lateral Flow Enzyme Immunoassay for Progesterone Detection in Whole Cows’ Milk. Talanta.

[138] Hendrickson O.D., Zvereva E.A., Panferov V.G., Solopova O.N., Zherdev A.V., Sveshnikov P.G., Dzantiev B.B. (2022). Application of Au@Pt Nanozyme as Enhancing Label for the Sensitive Lateral Flow Immunoassay of Okadaic Acid. Biosensors.

[139] Hendrickson O.D., Zvereva E.A., Zherdev A.V., Dzantiev B.B. (2022). Ultrasensitive Lateral Flow Immunoassay of Phycotoxin Microcystin-LR in Seafood Based on Magnetic Particles and Peroxidase Signal Amplification. Food Control.

[140] Shu Q., Wang L., Ouyang H., Wang W., Liu F., Fu Z. (2017). Multiplexed Immunochromatographic Test Strip for Time-Resolved Chemiluminescent Detection of Pesticide Residues Using a Bifunctional Antibody. Biosens. Bioelectron..

[141] Wang J., Majkova Z., Bever C.R.S., Yang J., Gee S.J., Li J., Xu T., Hammock B.D. (2015). One-Step Immunoassay for Tetrabromobisphenol A Using a Camelid Single Domain Antibody–Alkaline Phosphatase Fusion Protein. Anal. Chem..

[142] Manes T., Hoylaerts M.F., Müller R., Lottspeich F., Hölke W., Millán J.L. (1998). Genetic Complexity, Structure, and Characterization of Highly Active Bovine Intestinal Alkaline Phosphatases. J. Biol. Chem..

[143] Fosset M., Chappelet-Tordo D., Lazdunski M. (1974). Intestinal Alkaline Phosphatase. Physical Properties and Quaternary Structure. Biochemistry.

[144] Neumann H., Lustig A. (1980). The Activation of Alkaline Phosphatase by Effector Molecules. A Combined Kinetic and Hydrodynamic Study. Eur. J. Biochem..

[145] Rennke H.G., Venkatachalam M.A. (1979). Chemical Modification of Horseradish Peroxidase. Preparation and Characterization of Tracer Enzymes with Different Isoelectric Points. J. Histochem. Cytochem..

[146] Sotnikov D.V., Berlina A.N., Ivanov V.S., Zherdev A.V., Dzantiev B.B. (2019). Adsorption of Proteins on Gold Nanoparticles: One or More Layers?. Colloids Surf. B Biointerfaces.

[147] Kim L.H.-Y., Plaza K., Thomas S.R., Draijer C., Radford K., Peters-Golden M., Mukherjee M., Nair P. (2018). Endogenous Peroxidases in Sputum Interfere with Horse-Radish Peroxidase-Based ELISAs. J. Immunol. Methods.

[148] Jiang S., Penner M.H. (2017). Overcoming Reductant Interference in Peroxidase-Based Assays for Hydrogen Peroxide Quantification. J. Agric. Food Chem..

[149] Patsoukis N., Papapostolou I., Georgiou C.D. (2005). Interference of Non-Specific Peroxidases in the Fluorescence Detection of Superoxide Radical by Hydroethidine Oxidation: A New Assay for H_2_O_2_. Anal. Bioanal. Chem..

[150] Abraham A., Albrechtsen S.E. (2001). Comparison of Penicillinase, Urease and Alkaline Phosphatase as Labels in Enzyme-Linked Immunosorbent Assay (ELISA) for the Detection of Plant Viruses. J. Plant Dis. Prot..

[151] Europe O. (2015). PM 7/125 (1) ELISA Tests for Viruses. EPPO Bull..

[152] Frey A., Meckelein B., Externest D., Schmidt M.A. (2000). A Stable and Highly Sensitive 3,3′,5,5′-Tetramethylbenzidine-Based Substrate Reagent for Enzyme-Linked Immunosorbent Assays. J. Immunol. Methods.

[153] Ramachandran S., Fu E., Lutz B., Yager P. (2014). Long-Term Dry Storage of an Enzyme-Based Reagent System for ELISA in Point-of-Care Devices. Analyst.

[154] Zhang J., Gui X., Zheng Q., Chen Y., Ge S., Zhang J., Xia N. (2019). An HRP-Labeled Lateral Flow Immunoassay for Rapid Simultaneous Detection and Differentiation of Influenza A and B Viruses. J. Med. Virol..

[155] Byers K.M., Bird A.R., Cho H.D.D., Linnes J.C. (2020). Fully Dried Two-Dimensional Paper Network for Enzymatically Enhanced Detection of Nucleic Acid Amplicons. ACS Omega.

[156] Joung H.A., Oh Y.K., Kim M.G. (2014). An Automatic Enzyme Immunoassay Based on a Chemiluminescent Lateral Flow Immunosensor. Biosens. Bioelectron..

[157] Kim K., Joung H.A., Han G.R., Kim M.G. (2016). An Immunochromatographic Biosensor Combined with a Water-Swellable Polymer for Automatic Signal Generation or Amplification. Biosens. Bioelectron..

[158] Gao L., Zhuang J., Nie L., Zhang J., Zhang Y., Gu N., Wang T., Feng J., Yang D., Perrett S. (2007). Intrinsic Peroxidase-like Activity of Ferromagnetic Nanoparticles. Nat. Nanotechnol..

[159] Wu J., Wang X., Wang Q., Lou Z., Li S., Zhu Y., Qin L., Wei H. (2019). Nanomaterials with Enzyme-like Characteristics (Nanozymes): Next-Generation Artificial Enzymes (II). Chem. Soc. Rev..

[160] Huang Y., Ren J., Qu X. (2019). Nanozymes: Classification, Catalytic Mechanisms, Activity Regulation, and Applications. Chem. Rev..

[161] Wei Z., Xi Z., Vlasov S., Ayala J., Xia X. (2020). Nanocrystals of Platinum-Group Metals as Peroxidase Mimics for in Vitro Diagnostics. Chem. Commun..

[162] Gao Z., Ye H., Tang D., Tao J., Habibi S., Minerick A., Tang D., Xia X. (2017). Platinum-Decorated Gold Nanoparticles with Dual Functionalities for Ultrasensitive Colorimetric in Vitro Diagnostics. Nano Lett..

[163] Yang H., He Q., Pan J., Shen D., Xiao H., Cui X., Zhao S. (2021). A Pt-Ir Nanocube Amplified Lateral Flow Immunoassay for Dehydroepiandrosterone. Analyst.

[164] He Q., Yang H., Chen Y., Shen D., Cui X., Zhang C., Xiao H., Eremin S.A., Fang Y., Zhao S. (2020). Prussian Blue Nanoparticles with Peroxidase-Mimicking Properties in a Dual Immunoassays for Glycocholic Acid. J. Pharm. Biomed. Anal..

[165] Zhao B., Huang Q., Dou L., Bu T., Chen K., Yang Q., Yan L., Wang J., Zhang D. (2018). Prussian Blue Nanoparticles Based Lateral Flow Assay for High Sensitive Determination of Clenbuterol. Sens. Actuators B Chem..

[166] Luo Y., Luo H., Zou S., Jiang J., Duan D., Chen L., Gao L. (2023). An In Situ Study on Nanozyme Performance to Optimize Nanozyme-Strip for Aβ Detection. Sensors.

[167] Yang D., Wang L., Jia T., Lian T., Yang K., Li X., Wang X., Xue C. (2023). Au/Fe3O4-Based Nanozymes with Peroxidase-like Activity Integrated in Immunochromatographic Strips for Highly-Sensitive Biomarker Detection. Anal. Methods.

[168] Jiang T., Song Y., Du D., Liu X., Lin Y. (2016). Detection of P53 Protein Based on Mesoporous Pt-Pd Nanoparticles with Enhanced Peroxidase-like Catalysis. ACS Sens..

[169] Zhang J., Yu Q., Qiu W., Li K., Qian L., Zhang X., Liu G. (2019). Gold-Platinum Nanoflowers as a Label and as an Enzyme Mimic for Use in Highly Sensitive Lateral Flow Immunoassays: Application to Detection of Rabbit IgG. Microchim. Acta.

[170] Duan D., Fan K., Zhang D., Tan S., Liang M., Liu Y., Zhang J., Zhang P., Liu W., Qiu X. (2015). Nanozyme-Strip for Rapid Local Diagnosis of Ebola. Biosens. Bioelectron..

[171] Panferov V.G., Safenkova I.V., Zherdev A.V., Dzantiev B.B. (2020). Urchin Peroxidase-Mimicking Au@Pt Nanoparticles as a Label in Lateral Flow Immunoassay: Impact of Nanoparticle Composition on Detection Limit of Clavibacter Michiganensis. Microchim. Acta.

[172] Park J.-M., Jung H.-W., Chang Y.W., Kim H.-S., Kang M.-J., Pyun J.-C. (2015). Chemiluminescence Lateral Flow Immunoassay Based on Pt Nanoparticle with Peroxidase Activity. Anal. Chim. Acta.

[173] Li N., Xi X., Zhu J., Wu X., Zhang X., Wang S., Wen W. (2022). High Sensitivity and Rapid Detection of Hepatitis B Virus DNA Using Lateral Flow Biosensors Based on Au@Pt Nanorods in the Absence of Hydrogen Peroxide. Analyst.

[174] Kong D.Y., Heo N.S., Kang J.W., Lee J.B., Kim H.J., Kim M. (2022). Il Nanoceria-Based Lateral Flow Immunoassay for Hydrogen Peroxide-Free Colorimetric Biosensing for C-Reactive Protein. Anal. Bioanal. Chem..

[175] Cai X., Liang M., Ma F., Zhang Z., Tang X., Jiang J., Guo C., Ramzy Mohamed S., Abdel Goda A., Dawood D.H. (2022). Nanozyme-Strip Based on MnO2 Nanosheets as a Catalytic Label for Multi-Scale Detection of Aflatoxin B1 with an Ultrabroad Working Range. Food Chem..

[176] Lin B., Guan Z., Song Y., Song E., Lu Z., Liu D., An Y., Zhu Z., Zhou L., Yang C. (2018). Lateral Flow Assay with Pressure Meter Readout for Rapid Point-of-Care Detection of Disease-Associated Protein. Lab Chip.

[177] Huang D., Lin B., Song Y., Guan Z., Cheng J., Zhu Z., Yang C. (2019). Staining Traditional Colloidal Gold Test Strips with Pt Nanoshell Enables Quantitative Point-of-Care Testing with Simple and Portable Pressure Meter Readout. ACS Appl. Mater. Interfaces.

[178] Liu L., Zhao G., Dou W. (2020). An Unplugged and Quantitative Foam Based Immunochromatographic Assay for *Escherichia coli* O157:H7 Using Nanozymes to Catalyze Hydrogen Peroxide Decomposition Reaction. Microchem. J..

[179] Liu L., Liu J., Huang H., Li Y., Zhao G., Dou W. (2019). A Quantitative Foam Immunoassay for Detection of *Escherichia coli* O157:H7 Based on Bimetallic Nanocatalyst-gold Platinum. Microchem. J..

[180] Wang K.-Y., Bu S.-J., Ju C.-J., Han Y., Ma C.-Y., Liu W.-S., Li Z.-Y., Li C.-T., Wan J.-Y. (2019). Disposable Syringe-Based Visual Immunotest for Pathogenic Bacteria Based on the Catalase Mimicking Activity of Platinum Nanoparticle-Concanavalin A Hybrid Nanoflowers. Microchim. Acta.

[181] Yu Z., Cai G., Liu X., Tang D. (2020). Platinum Nanozyme-Triggered Pressure-Based Immunoassay Using a Three-Dimensional Polypyrrole Foam-Based Flexible Pressure Sensor. ACS Appl. Mater. Interfaces.

[182] Wei Z., Luciano K., Xia X. (2022). Catalytic Gold-Iridium Nanoparticles as Labels for Sensitive Colorimetric Lateral Flow Assay. ACS Nano.

[183] Zhou Y., Liu B., Yang R., Liu J. (2017). Filling in the Gaps between Nanozymes and Enzymes: Challenges and Opportunities. Bioconjug. Chem..

[184] Gao Z., Shao S., Gao W., Tang D., Tang D., Zou S., Kim M.J., Xia X. (2021). Morphology-Invariant Metallic Nanoparticles with Tunable Plasmonic Properties. ACS Nano.

[185] Yang T., Ahn J., Shi S., Wang P., Gao R., Qin D. (2021). Noble-Metal Nanoframes and Their Catalytic Applications. Chem. Rev..

[186] Wang Y., Xianyu Y. (2023). Tuning the Plasmonic and Catalytic Signals of Au@Pt Nanoparticles for Dual-Mode Biosensing. Biosens. Bioelectron..

[187] Jiao L., Xu W., Wu Y., Yan H., Gu W., Du D., Lin Y., Zhu C. (2021). Single-Atom Catalysts Boost Signal Amplification for Biosensing. Chem. Soc. Rev..

[188] Kong L., Chen D., Zhang X., Zhou L., Deng Y., Wei S. (2023). Controllable Fabrication of Hg–Pd–PdO Heterostructures as Efficient Peroxidase Mimics for Carcinoembryonic Antigen Detection. ACS Appl. Nano Mater..

[189] Huang Z., Liu B., Liu J. (2020). Enhancing the Peroxidase-like Activity and Stability of Gold Nanoparticles by Coating a Partial Iron Phosphate Shell. Nanoscale.

[190] Jin S., Wu C., Ye Z., Ying Y. (2019). Designed Inorganic Nanomaterials for Intrinsic Peroxidase Mimics: A Review. Sens. Actuators B Chem..

[191] Hermanson G.T., Hermanson G.-B.T. (2013). Antibody Modification and Conjugation. Bioconjugate Techniques.

[192] Nakane K. (1974). Peroxidase-Labeled a New Antibody Method Conjugation. J. Histochem. Cytochem..

[193] Tao X., Wang X., Liu B., Liu J. (2020). Conjugation of Antibodies and Aptamers on Nanozymes for Developing Biosensors. Biosens. Bioelectron..

[194] Gao Z., Xu M., Hou L., Chen G., Tang D. (2013). Irregular-Shaped Platinum Nanoparticles as Peroxidase Mimics for Highly Efficient Colorimetric Immunoassay. Anal. Chim. Acta.

[195] Gao Z., Xu M., Lu M., Chen G., Tang D. (2015). Urchin-like (Gold Core)@(Platinum Shell) Nanohybrids: A Highly Efficient Peroxidase-Mimetic System for in Situ Amplified Colorimetric Immunoassay. Biosens. Bioelectron..

[196] Lai X., Zhang G., Zeng L., Xiao X., Peng J., Guo P., Zhang W., Lai W. (2021). Synthesis of PDA-Mediated Magnetic Bimetallic Nanozyme and Its Application in Immunochromatographic Assay. ACS Appl. Mater. Interfaces.

[197] Chen Y., Ren J., Yin X., Li Y., Shu R., Wang J., Zhang D. (2022). Vanadium Disulfide Nanosheet Boosts Optical Signal Brightness as a Superior Enzyme Label to Improve the Sensitivity of Lateral Flow Immunoassay. Anal. Chem..

[198] Liang J., Liu Z., Fang Y., Shen X., Xu Z., Lei H., Huang X., Li X. (2023). Two Kinds of Lateral Flow Immunoassays Based on Multifunctional Magnetic Prussian Blue Nanoenzyme and Colloidal Gold for the Detection of 38 β-Agonists in Swine Urine and Pork. Food Chem..

[199] Xia X., Zhang J., Lu N., Kim M.J., Ghale K., Xu Y., McKenzie E., Liu J., Ye H. (2015). Pd-Ir Core-Shell Nanocubes: A Type of Highly Efficient and Versatile Peroxidase Mimic. ACS Nano.

[200] Ye H., Yang K., Tao J., Liu Y., Zhang Q., Habibi S., Nie Z., Xia X. (2017). An Enzyme-Free Signal Amplification Technique for Ultrasensitive Colorimetric Assay of Disease Biomarkers. ACS Nano.

[201] Tian M., Xie W., Zhang T., Liu Y., Lu Z., Li C.M., Liu Y. (2020). A Sensitive Lateral Flow Immunochromatographic Strip with Prussian Blue Nanoparticles Mediated Signal Generation and Cascade Amplification. Sens. Actuators B Chem..

[202] Hendrickson O.D., Zvereva E.A., Pridvorova S.M., Dzantiev B.B., Zherdev A. (2023). V The Use of Au@Pt Nanozyme to Perform Ultrasensitive Immunochromatographic Detection of Banned Pork Additives in Meat Products. Food Control.

[203] Chen Z.J., Huang Z., Huang S., Zhao J.L., Sun Y., Xu Z.L., Liu J. (2021). Effect of Proteins on the Oxidase-like Activity of CeO2nanozymes for Immunoassays. Analyst.

[204] McVey C., Logan N., Thanh N.T.K., Elliott C., Cao C. (2019). Unusual Switchable Peroxidase-Mimicking Nanozyme for the Determination of Proteolytic Biomarker. Nano Res..

[205] Chen R., Chen X., Zhou Y., Lin T., Leng Y., Huang X., Xiong Y. (2022). “Three-in-One” Multifunctional Nanohybrids with Colorimetric Magnetic Catalytic Activities to Enhance Immunochromatographic Diagnosis. ACS Nano.

[206] Zheng C., Jiang Q., Wang K., Li T., Zheng W., Cheng Y., Ning Q., Cui D. (2022). Nanozyme Enhanced Magnetic Immunoassay for Dual-Mode Detection of Gastrin-17. Analyst.

[207] Kim M.S., Kweon S.H., Cho S., An S.S.A., Kim M.I., Doh J., Lee J. (2017). Pt-Decorated Magnetic Nanozymes for Facile and Sensitive Point-of-Care Bioassay. ACS Appl. Mater. Interfaces.

[208] Liang M., Cai X., Gao Y., Yan H., Fu J., Tang X., Zhang Q., Li P. (2022). A Versatile Nanozyme Integrated Colorimetric and Photothermal Lateral Flow Immunoassay for Highly Sensitive and Reliable Aspergillus Flavus Detection. Biosens. Bioelectron..

[209] Zhang X., Zhu X., Li Y., Hai X., Bi S. (2023). A Colorimetric and Photothermal Dual-Mode Biosensing Platform Based on Nanozyme-Functionalized Flower-like DNA Structures for Tumor-Derived Exosome Detection. Talanta.

[210] Guteneva N.V., Znoyko S.L., Orlov A.V., Nikitin M.P., Nikitin P.I. (2019). Rapid Lateral Flow Assays Based on the Quantification of Magnetic Nanoparticle Labels for Multiplexed Immunodetection of Small Molecules: Application to the Determination of Drugs of Abuse. Microchim. Acta.

[211] Zhu Y., Zhao R., Feng L., Wang C., Dong S., Zyuzin M.V., Timin A., Hu N., Liu B., Yang P. (2023). Dual Nanozyme-Driven PtSn Bimetallic Nanoclusters for Metal-Enhanced Tumor Photothermal and Catalytic Therapy. ACS Nano.

[212] Gao Z., Lv S., Xu M., Tang D. (2017). High-Index {: Hk 0} Faceted Platinum Concave Nanocubes with Enhanced Peroxidase-like Activity for an Ultrasensitive Colorimetric Immunoassay of the Human Prostate-Specific Antigen. Analyst.

[213] Chen T.M., Tian X.M., Huang L., Xiao J., Yang G.W. (2017). Nanodiamonds as PH-Switchable Oxidation and Reduction Catalysts with Enzyme-like Activities for Immunoassay and Antioxidant Applications. Nanoscale.

[214] Yang H., He Q., Chen Y., Shen D., Xiao H., Eremin S.A., Cui X., Zhao S. (2020). Platinum Nanoflowers with Peroxidase-like Property in a Dual Immunoassay for Dehydroepiandrosterone. Microchim. Acta.

[215] Panferov V.G., Safenkova I.V., Zherdev A.V., Dzantiev B.B. (2021). The Steadfast Au@Pt Soldier: Peroxide-Tolerant Nanozyme for Signal Enhancement in Lateral Flow Immunoassay of Peroxidase-Containing Samples. Talanta.

[216] Khramtsov P., Kropaneva M., Minin A., Bochkova M., Timganova V., Maximov A., Puzik A., Zamorina S., Rayev M. (2022). Prussian Blue Nanozymes with Enhanced Catalytic Activity: Size Tuning and Application in ELISA-like Immunoassay. Nanomaterials.

[217] Asati A., Santra S., Kaittanis C., Nath S., Perez J.M. (2009). Oxidase-Like Activity of Polymer-Coated Cerium Oxide Nanoparticles. Angew. Chem. Int. Ed..

[218] Fu Y., Zhang H., Dai S., Zhi X., Zhang J., Li W. (2015). Glutathione-Stabilized Palladium Nanozyme for Colorimetric Assay of Silver(I) Ions. Analyst.

[219] Singh N., Savanur M.A., Srivastava S., D’Silva P., Mugesh G. (2017). A Redox Modulatory Mn_3_O_4_ Nanozyme with Multi-Enzyme Activity Provides Efficient Cytoprotection to Human Cells in a Parkinson’s Disease Model. Angew. Chemie Int. Ed..

[220] Tian R., Sun J., Qi Y., Zhang B., Guo S., Zhao M. (2017). Influence of VO_2_ Nanoparticle Morphology on the Colorimetric Assay of H_2_O_2_ and Glucose. Nanomaterials.

[221] Liu S., Lu F., Xing R., Zhu J.J. (2011). Structural Effects of Fe_3_O_4_ Nanocrystals on Peroxidase-like Activity. Chem. Eur. J..

[222] Robert A., Meunier B. (2022). How to Define a Nanozyme. ACS Nano.

[223] Zandieh M., Liu J. (2023). Nanozymes: Definition, Activity, and Mechanisms. Adv. Mater..

[224] Loynachan C.N., Thomas M.R., Gray E.R., Richards D.A., Kim J., Miller B.S., Brookes J.C., Agarwal S., Chudasama V., McKendry R.A. (2018). Platinum Nanocatalyst Amplification: Redefining the Gold Standard for Lateral Flow Immunoassays with Ultrabroad Dynamic Range. ACS Nano.

[225] Mu J., Wang Y., Zhao M., Zhang L. (2012). Intrinsic Peroxidase-like Activity and Catalase-like Activity of Co_3_O_4_ Nanoparticles. Chem. Commun..

[226] Punekar N.S., Punekar N.S. (2018). Good Kinetic Practices. ENZYMES: Catalysis, Kinetics and Mechanisms.

[227] Jin X., Chen L., Zhang Y., Wang X., Zhou N. (2021). A Lateral Flow Strip for On-Site Detection of Tobramycin Based on Dual-Functional Platinum-Decorated Gold Nanoparticles. Analyst.

[228] Zheng X., Liu Q., Jing C., Li Y., Li D., Luo W., Wen Y., He Y., Huang Q., Long Y.-T. (2011). Catalytic Gold Nanoparticles for Nanoplasmonic Detection of DNA Hybridization. Angew. Chem..

[229] Li J., Liu F., Zhu Z., Liu D., Chen X., Song Y., Zhou L., Yang C. (2018). In Situ Pt Staining Method for Simple, Stable, and Sensitive Pressure-Based Bioassays. ACS Appl. Mater. Interfaces.

[230] Fu J., Zhou Y., Huang X., Zhang W., Wu Y., Fang H., Zhang C., Xiong Y. (2020). Dramatically Enhanced Immunochromatographic Assay Using Cascade Signal Amplification for Ultrasensitive Detection of *Escherichia coli* O157:H7 in Milk. J. Agric. Food Chem..

[231] Sun Y., Xie Z., Pei F., Hu W., Feng S., Hao Q., Liu B., Mu X., Lei W., Tong Z. (2022). Trimetallic Au@Pd@Pt Nanozyme-Enhanced Lateral Flow Immunoassay for the Detection of SARS-CoV-2 Nucleocapsid Protein. Anal. Methods.

[232] Lyu Z., Ding S., Tieu P., Fang L., Li X., Li T., Pan X., Engelhard M.H., Ruan X., Du D. (2022). Single-Atomic Site Catalyst Enhanced Lateral Flow Immunoassay for Point-of-Care Detection of Herbicide. Research.

[233] Cai X., Ma F., Jiang J., Yang X., Zhang Z., Jian Z., Liang M., Li P., Yu L. (2023). Fe-N-C Single-Atom Nanozyme for Ultrasensitive, on-Site and Multiplex Detection of Mycotoxins Using Lateral Flow Immunoassay. J. Hazard. Mater..

[234] Xia X., Figueroa-Cosme L., Tao J., Peng H.-C., Niu G., Zhu Y., Xia Y. (2014). Facile Synthesis of Iridium Nanocrystals with Well-Controlled Facets Using Seed-Mediated Growth. J. Am. Chem. Soc..

[235] Lori O., Elbaz L. (2020). Recent Advances in Synthesis and Utilization of Ultra-low Loading of Precious Metal-based Catalysts for Fuel Cells. ChemCatChem.

[236] Cho J.-H., Han S., Paek E.-H., Cho I., Paek S. (2006). Plastic ELISA-on-a-Chip Based on Sequential Cross-Flow Chromatography. Anal. Chem..

[237] Cho I.H., Paek E.H., Kim Y.K., Kim J.H., Paek S.H. (2009). Chemiluminometric Enzyme-Linked Immunosorbent Assays (ELISA)-on-a-Chip Biosensor Based on Cross-Flow Chromatography. Anal. Chim. Acta.

[238] Cho J.H., Kim M.H., Mok R.S., Jeon J.W., Lim G.S., Chai C.Y., Paek S.H. (2014). Two-Dimensional Paper Chromatography-Based Fluorescent Immunosensor for Detecting Acute Myocardial Infarction Markers. J. Chromatogr. B Anal. Technol. Biomed. Life Sci..

[239] Lim G.-S., Seo S.-M., Paek S.-H., Kim S.-W., Jeon J.-W., Kim D.-H., Cho I.-H., Paek S.-H. (2015). Chemiluminometric Immunosensor for High-Sensitivity Cardiac Troponin I Employing a Polymerized Enzyme Conjugate as a Tracer. Sci. Rep..

[240] Cho I.H., Seo S.M., Paek E.H., Paek S.H. (2010). Immunogold-Silver Staining-on-a-Chip Biosensor Based on Cross-Flow Chromatography. J. Chromatogr. B Anal. Technol. Biomed. Life Sci..

[241] Shin J.H., Hong J., Go H., Park J., Kong M., Ryu S., Kim K.-P., Roh E., Park J.-K. (2018). Multiplexed Detection of Foodborne Pathogens from Contaminated Lettuces Using a Handheld Multistep Lateral Flow Assay Device. J. Agric. Food Chem..

[242] Panraksa Y., Apilux A., Jampasa S., Puthong S., Henry C.S., Rengpipat S., Chailapakul O. (2021). A Facile One-Step Gold Nanoparticles Enhancement Based on Sequential Patterned Lateral Flow Immunoassay Device for C-Reactive Protein Detection. Sens. Actuators B Chem..

[243] Deng J., Yang M., Wu J., Zhang W., Jiang X. (2018). A Self-Contained Chemiluminescent Lateral Flow Assay for Point-of-Care Testing. Anal. Chem..

[244] Yang W., Li X., Liu G., Zhang B., Zhang Y., Kong T., Tang J., Li D., Wang Z. (2011). A Colloidal Gold Probe-Based Silver Enhancement Immunochromatographic Assay for the Rapid Detection of Abrin-A. Biosens. Bioelectron..

[245] Panferov V.G., Safenkova I.V., Varitsev Y.A., Drenova N.V., Kornev K.P., Zherdev A.V., Dzantiev B.B. (2016). Development of the Sensitive Lateral Flow Immunoassay with Silver Enhancement for the Detection of Ralstonia Solanacearum in Potato Tubers. Talanta.

[246] Zangheri M., Di Nardo F., Mirasoli M., Anfossi L., Nascetti A., Caputo D., De Cesare G., Guardigli M., Baggiani C., Roda A. (2016). Chemiluminescence Lateral Flow Immunoassay Cartridge with Integrated Amorphous Silicon Photosensors Array for Human Serum Albumin Detection in Urine Samples. Anal. Bioanal. Chem..

[247] Zangheri M., Mirasoli M., Guardigli M., Di Nardo F., Anfossi L., Baggiani C., Simoni P., Benassai M., Roda A. (2019). Chemiluminescence-Based Biosensor for Monitoring Astronauts’ Health Status during Space Missions: Results from the International Space Station. Biosens. Bioelectron..

[248] Zangheri M., Di Nardo F., Calabria D., Marchegiani E., Anfossi L., Guardigli M., Mirasoli M., Baggiani C., Roda A. (2021). Smartphone Biosensor for Point-of-Need Chemiluminescence Detection of Ochratoxin A in Wine and Coffee. Anal. Chim. Acta.

[249] Shin J.H., Park J.K. (2016). Functional Packaging of Lateral Flow Strip Allows Simple Delivery of Multiple Reagents for Multistep Assays. Anal. Chem..

[250] Panraksa Y., Jang I., Carrell C.S., Amin A.G., Chailapakul O., Chatterjee D., Henry C.S. (2022). Simple Manipulation of Enzyme-Linked Immunosorbent Assay (ELISA) Using an Automated Microfluidic Interface. Anal. Methods.

[251] Han K.N., Choi J.-S., Kwon J. (2016). Three-Dimensional Paper-Based Slip Device for One-Step Point-of-Care Testing. Sci. Rep..

[252] Park J., Shin J.H., Park J.K. (2016). Pressed Paper-Based Dipstick for Detection of Foodborne Pathogens with Multistep Reactions. Anal. Chem..

[253] Preechakasedkit P., Teekayupak K., Citterio D., Ruecha N. (2022). Improvement in Sensitivity for Lateral Flow Immunoassay of Ferritin Using Novel Device Design Based on Gold-Enhanced Gold Nanoparticles. Sci. Rep..

[254] Fu E., Kauffman P., Lutz B., Yager P. (2010). Chemical Signal Amplification in Two-Dimensional Paper Networks. Sens. Actuators B Chem..

[255] Fu E., Liang T., Houghtaling J., Ramachandran S., Ramsey S.A., Lutz B., Yager P. (2011). Enhanced Sensitivity of Lateral Flow Tests Using a Two-Dimensional Paper Network Format. Anal. Chem..

[256] Fu E., Liang T., Spicar-Mihalic P., Houghtaling J., Ramachandran S., Yager P. (2012). Two-Dimensional Paper Network Format That Enables Simple Multistep Assays for Use in Low-Resource Settings in the Context of Malaria Antigen Detection. Anal. Chem..

[257] Lutz B., Liang T., Fu E., Ramachandran S., Kauffman P., Yager P. (2013). Dissolvable Fluidic Time Delays for Programming Multi-Step Assays in Instrument-Free Paper Diagnostics. Lab Chip.

[258] Mulvaney S.P., Kidwell D.A., Lanese J.N., Lopez R.P., Sumera M.E., Wei E. (2020). Catalytic Lateral Flow Immunoassays (CLFIA^TM^): Amplified Signal in a Self-Contained Assay Format. Sens. Bio-Sens. Res..

[259] Han D.K., Oh J., Lee J., Cho Y.G., Park J.S., Choi J.S., Kim D.S., Kwon J. (2021). Paper-Based Multiplex Analytical Device for Simultaneous Detection of Clostridioides Difficile Toxins and Glutamate Dehydrogenase. Biosens. Bioelectron..

[260] Kim W., Lee S., Jeon S. (2018). Enhanced Sensitivity of Lateral Flow Immunoassays by Using Water-Soluble Nanofibers and Silver-Enhancement Reactions. Sens. Actuators B Chem..

[261] Yang X., Huang R., Xiong L., Chen F., Sun W., Yu L. (2023). A Colorimetric Aptasensor for Ochratoxin A Detection Based on Tetramethylrhodamine Charge Effect-Assisted Silver Enhancement. Biosensors.

[262] Komkova M.A., Karyakina E.E., Karyakin A.A. (2018). Catalytically Synthesized Prussian Blue Nanoparticles Defeating Natural Enzyme Peroxidase. J. Am. Chem. Soc..

[263] Xi Z., Wei K., Wang Q., Kim M.J., Sun S., Fung V., Xia X. (2021). Nickel–Platinum Nanoparticles as Peroxidase Mimics with a Record High Catalytic Efficiency. J. Am. Chem. Soc..

[264] Wu J., Qin K., Yuan D., Tan J., Qin L., Zhang X., Wei H. (2018). Rational Design of Au@Pt Multibranched Nanostructures as Bifunctional Nanozymes. ACS Appl. Mater. Interfaces.

[265] Porstmann B., Porstmann T., Nugel E., Evers U. (1985). Which of the Commonly Used Marker Enzymes Gives the Best Results in Colorimetric and Fluorimetric Enzyme Immunoassays: Horseradish Peroxidase, Alkaline Phosphatase or β-Galactosidase?. J. Immunol. Methods.

[266] Lakshmi Kumari G., Dhir R.N. (2003). Comparative Studies with Penicillinase, Horseradish Peroxidase, and Alkaline Phosphatase as Enzyme Labels in Developing Enzyme Immunoassay of Cortisol. J. Immunoass. Immunochem..

[267] He J., Wild D.B.T.-T.I.H. (2013). Practical Guide to ELISA Development. The Immunoassay Handbook.

[268] Han G.R., Koo H.J., Ki H., Kim M.G. (2020). Paper/Soluble Polymer Hybrid-Based Lateral Flow Biosensing Platform for High-Performance Point-of-Care Testing. ACS Appl. Mater. Interfaces.

[269] Dargatz D.A., Byrum B.A., Collins M.T., Goyal S.M., Hietala S.K., Jacobson R.H., Kopral C.A., Martin B.M., McCluskey B.J., Tewari D. (2004). A Multilaboratory Evaluation of a Commercial Enzyme-Linked Immunosorbent Assay Test for the Detection of Antibodies against Mycobacterium Avium Subsp. Paratuberculosis in Cattle. J. Vet. Diagn. Investig..

[270] Pecson B.M., Darby E., Haas C.N., Amha Y.M., Bartolo M., Danielson R., Dearborn Y., Di Giovanni G., Ferguson C., Fevig S. (2021). Reproducibility and Sensitivity of 36 Methods to Quantify the SARS-CoV-2 Genetic Signal in Raw Wastewater: Findings from an Interlaboratory Methods Evaluation in the U.S. Environ. Sci. Water Res. Technol..

[271] Ley B., Satyagraha A.W., Kibria M.G., Armstrong J., Bancone G., Bei A.K., Bizilj G., Brito M., Ding X.C., Domingo G.J. (2022). Repeatability and Reproducibility of a Handheld Quantitative G6PD Diagnostic. PLoS Negl. Trop. Dis..

[272] Farka Z., Mickert M.J., Pastucha M., Mikušová Z., Skládal P., Gorris H.H. (2020). Advances in Optical Single-Molecule Detection: En Route to Supersensitive Bioaffinity Assays. Angew. Chem. Int. Ed..

[273] Parolo C., Sena-Torralba A., Bergua J.F., Calucho E., Fuentes-Chust C., Hu L., Rivas L., Álvarez-Diduk R., Nguyen E.P., Cinti S. (2020). Tutorial: Design and Fabrication of Nanoparticle-Based Lateral-Flow Immunoassays. Nat. Protoc..

[274] Hsieh H., Dantzler J., Weigl B. (2017). Analytical Tools to Improve Optimization Procedures for Lateral Flow Assays. Diagnostics.

[275] Millipore M. Rapid Lateral Flow Test Strips Considerations for Product Development. https://www.merckmillipore.com/INTERSHOP/web/WFS/Merck-RU-Site/ru_RU/-/USD/ShowDocument-Pronet?id=201306.15671.

[276] Moosavi S.M., Ghassabian S., Stauffer M.T. (2018). Linearity of Calibration Curves for Analytical Methods: A Review of Criteria for Assessment of Method Reliability. Calibration and Validation of Analytical Methods—A Sampling of Current Approaches.

[277] Bustin S.A., Benes V., Garson J.A., Hellemans J., Huggett J., Kubista M., Mueller R., Nolan T., Pfaffl M.W., Shipley G.L. (2009). The MIQE Guidelines: Minimum Information for Publication of Quantitative Real-Time PCR Experiments. Clin. Chem..

[278] World Health Organization The ACT-Accelerator: Two Years of Impact. https://www.who.int/publications/m/item/the-act-accelerator--two-years-of-impact.

[279] Davies B., Araghi M., Moshe M., Gao H., Bennet K., Jenkins J., Atchison C., Darzi A., Ashby D., Riley S. (2021). Acceptability, Usability, and Performance of Lateral Flow Immunoassay Tests for Severe Acute Respiratory Syndrome Coronavirus 2 Antibodies: REACT-2 Study of Self-Testing in Nonhealthcare Key Workers. Open Forum Infect. Dis..

[280] Møller I.J.B., Utke A.R., Rysgaard U.K., Østergaard L.J., Jespersen S. (2022). Diagnostic Performance, User Acceptability, and Safety of Unsupervised SARS-CoV-2 Rapid Antigen-Detecting Tests Performed at Home. Int. J. Infect. Dis..

[281] Zheng C., Wang K., Zheng W., Cheng Y., Li T., Cao B., Jin Q., Cui D. (2021). Rapid Developments in Lateral Flow Immunoassay for Nucleic Acid Detection. Analyst.

[282] Kim K., Lee B., Park J.H., Park J.-H., Lee K.J., Kwak T.J., Son T., Shin Y.-B., Im H., Kim M.-G. (2023). Rapid PCR Kit: Lateral Flow Paper Strip with Joule Heater for SARS-CoV-2 Detection. Mater. Horizons.

[283] Bender A.T., Sullivan B.P., Zhang J.Y., Juergens D.C., Lillis L., Boyle D.S., Posner J.D. (2021). HIV Detection from Human Serum with Paper-Based Isotachophoretic RNA Extraction and Reverse Transcription Recombinase Polymerase Amplification. Analyst.

[284] Sullivan B.P., Chou Y.S., Bender A.T., Martin C.D., Kaputa Z.G., March H., Song M., Posner J.D. (2022). Quantitative Isothermal Amplification on Paper Membranes Using Amplification Nucleation Site Analysis. Lab Chip.

[285] Bender A.T., Borysiak M.D., Levenson A.M., Lillis L., Boyle D.S., Posner J.D. (2018). Semiquantitative Nucleic Acid Test with Simultaneous Isotachophoretic Extraction and Amplification. Anal. Chem..

[286] Chang L., Li J., Wang L. (2016). Immuno-PCR: An Ultrasensitive Immunoassay for Biomolecular Detection. Anal. Chim. Acta.

[287] Kim S., Sikes H.D. (2020). Radical Polymerization Reactions for Amplified Biodetection Signals. Polym. Chem..

