# Supplementary material for: Post-Assay Chemical Enhancement for Highly Sensitive Lateral Flow Immunoassays: A Critical Review
Source: Biosensors (Basel). 2023 Sep 1;13(9):866. doi: 10.3390/bios13090866 (PMC10526817; doi:10.3390/bios13090866)
Supplement: Supplementary file 1 [file biosensors-13-00866-s001.zip › biosensors-2516199-supplementary.pdf]

Supplementary material

Post-Assay Chemical Enhancement for Highly Sensitive Lateral Flow Immunoassays: A Critical Review

Vasily G. Panferov [1,2], Anatoly V. Zherdev [1] and Boris B. Dzantiev [1,*]

[1] A.N. Bach Institute of Biochemistry, Research Center of Biotechnology of the Russian Academy of Sciences, , 119071 Moscow, Russia
[2] Department of Chemistry, York University, Toronto, ON M3J 1P3, Canada
* Correspondence: dzantiev@inbi.ras.ru

Table S1. Turnover numbers for HRP and nanozymes.

Type of catalyst	k_{cat}, s^{-1}	Ref
HRP	4.00×10^3	[1]
HRP	3.62×10^3	[2]
HRP	1.9×10^3	[3]
HRP	5.18×10^3	[4]
HRP	4×10^3	[5]
HRP	3.5×10^3	[6]
Pd-Ir nanoparticles<br>Pd nanoparticles	1.1×10^5<br>4.8×10^3	[7]
Fe_3O_4 nanoparticles	3.02×10^4	[1]
Apoferritin paired gold clusters	5.8×10^4	[8]
BSA-stabilized Au nanoclusters	7.24×10^4	[2]
Au nanoclusters	4.49×10^4	[4]
Co_3O_4	1.83×10^2	[9]
Gold nanoclusters<br>Gold nanoclusters deposited on graphene oxide	260 (pH = 4)<br>236 (pH = 4)	[5]
GNP	734.38	[10]
Pd@Pt<br>Pd	3.1×10^4<br>1.2×10^4	[11]
Pt	2.27×10^4	[12]
Fe_2O_3 nanoparticles coated with Prussian blue	3.43×10^3	[13]
High-index {hk0} faceted Pt concave nanocubes<br>Pt nanospehres	5.98×10^6<br>1.62×10^6	[14]

Pt nanoparticles stabilized with various molecules<br>PVP, MW 55 kDa<br>CTAB<br>PVP, MW 10 kDa<br>PVP, MW 360 kDa	 8.2×10^5<br>4.6×10^5<br>4.4×10^5<br>1.2×10^6	[15]
Rh nanoparticles	387	[16]
Au@Pt with various Au and Pt ratio<br>Au@Pt 0.10<br>Au@Pt 0.17<br>Au@Pt 0.25	 0.96×10^3<br>1.92×10^3<br>5.74×10^3	[17]
Au@Pt with various Au and Pt ratio<br>Au@Pt 0.17<br>Au@Pt 0.25	 8.2×10^3<br>1.4×10^4	[18]
Au@Pt<br>Au@PtCu	$1.5\pm0.1 \times 10^3$<br>$5.4\pm0.5 \times 10^3$	[19]
Au@Pt<br>Au@PtAg with various Ag concentration<br>Au@PtAg0.5<br>Au@PtAg0.33	$4.2\pm0.3 \times 10^2$<br><br>$1.3\pm0.1 \times 10^2$<br>$2.2\pm0.1 \times 10^2$	[20]
Pd-Ir cubes<br>Pd cubes	1.9×10^6<br>6.9×10^4	[21]
Co_3O_4	376	[22]
Fe_3O_4 nanoparticles	2.94×10^4	[23]
Fe_3O_4 nanoparticles<br>Fe_3O_4 nanotubes	3.02×10^4<br>9.81×10^5	[24]
$Mn_{0.5}Fe_{0.5}Fe_2O$ nanoparticles	1.4×10^4	[25]
MnO_2-BSA	192	[26]
MnO_2 of various shape<br>Nanosheet<br>Nanosphere<br>Nanowire	1.7×10^7<br>1.5×10^7<br>2.76×10^7<br>2.61×10^4	[27]
RuO_2 nanoparticles	5.4×10^3	[28]
MoS_2	0.55×10^4	[29]
Prussian blue nanoparticles	1.16×10^5	[30]
Prussian blue nanoparticles<br>Metal-organic framework encapsulated with Prussian blue nanoparticles	2.3×10^2<br>9.9×10^2	[31]
Fe_3O_4-Pt	2.2×10^3	[32]
Pd@Pt	6.5×10^4	[33]

Pd@Pt-HRP	1.9×10^5	
Au@Pt	1.48×10^3	[34]
Pd@Au	118	[35]
Au@HRP, various pH<br>3<br>4.7<br>7.4	 1.7×10^3<br>7.4×10^3<br>0.7×10^3	[6]
GNP	367.82	[36]
Prussian blue nanoparticles of various diameters, nm	10-1000000	[37]
Au<br>Au-Pt<br>Au-Ir	1.02×10^4<br>1.48×10^6<br>1.07×10^7	[38]
Ir/WO with various ratio Ir/W<br>Ir/W ≈ 10%<br>Ir/W ≈ 20%<br>Ir/W ≈ 30%	 1.3×10^4<br>5.7×10^4<br>3.5×10^4	[39]
Pt<br>Ni-Pt of various ratio<br>Ni-Pt2<br>Ni-Pt1<br>Ni-Pt0.5	9.7×10^5<br><br>8.0×10^6<br>4.5×10^7<br>1.7×10^7	[40]
Pd-Ir of various size, nm<br>3.3<br>5.9<br>9.8<br>13.0	 9.4×10^4<br>3.4×10^5<br>9.6×10^5<br>1.2×10^6	[41]

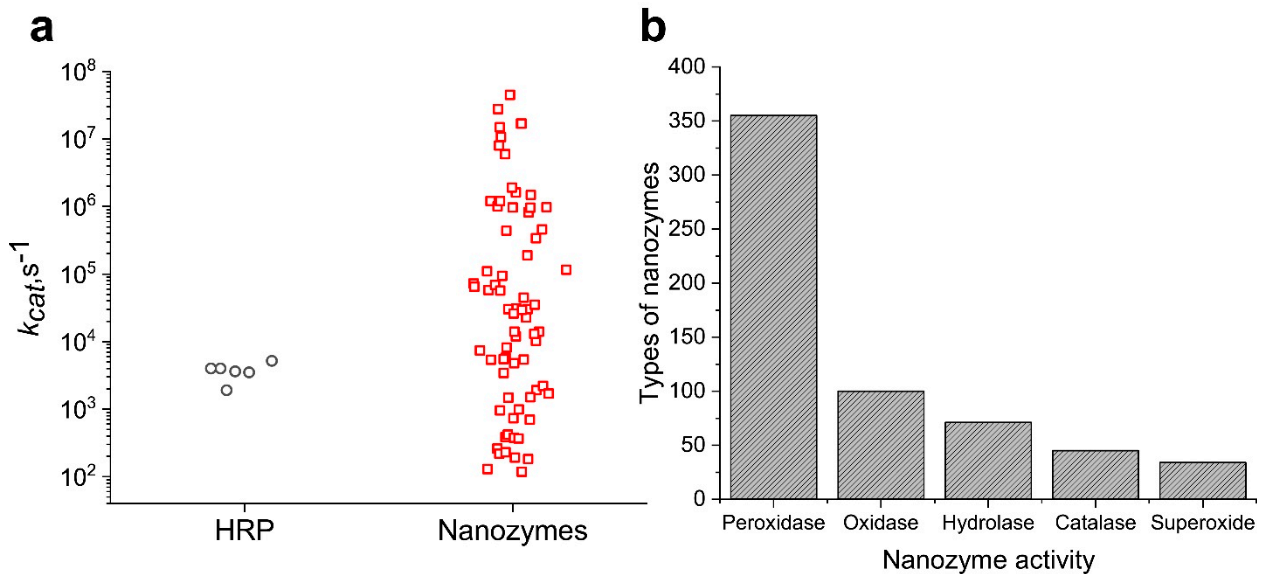

Figure S1. Characteristics of nanozymes as the catalytic labels. (a) the scatter plot of *k_{cat}* values for HRP and nanozymes (data in table S1); (b) types of nanozymes activity. Types of nanozymes activity were taken from [42].

Table S2. LOD values for various enhancement methods.

Target	LOD before enhancement	LOD after enhancement	LOD Enhancement, times	References
**Copper enhancement**				
P24 antigen	2 ng/mL	50 fg/mL	40000	[43]
E. coli	3.84 × 10^3 CFU/mL	6 CFU/mL	640	[43]
SARS-CoV-2 Ab	1:20 dilution	1:2000	100	[43]
Mycobacterium tuberculosis antigen CFP-10		7.6 pg/mL	8	[44]
Mycobacterium tuberculosis antigen 85B	1.57 ng/mL	0.21 ng/mL	7.5	[45]
E. coli	3.5 × 10^3 CFU/mL	25 CFU	140	[46]
Rabbit IgG	10 ng/mL	10 pg/mL	1000	[47]
Human chorionic gonadotropin	10 ng/mL	10 pg/mL	1000	[47]
Nucleocapsid protein SARS-CoV-2	10 ng/mL	10 pg/mL	1000	[48]
Bovine viral diarrhea virus			1426	[49]
**Silver enhancement**				
Mycobacterium tuberculosis antigen CFP-10		7.6 pg/mL	2	[44]
Receptor binding domain of SARS-CoV-2	488 pg/mL	61 pg/mL	8	[50]
Cardiac troponin I	0.82 ng/mL	0.016 ng/mL	51	[51]
Hemagglutinin of H5N1 avian influenza virus	250 ng/mL	0.5 ng/mL	250	[52]
Abrin-a	10 ng/mL	100 pg/mL	100	[53]
Potato virus X	4.8 ng/mL	-	10	[54]
Salmonella cells	10^7 cells/mL	10^4 cells/mL	1000	[55]
Ochratoxin A	11±4 μg/L	0.8±0.1 μg/L	14	[56]
Cadmium	20 μg/L	5 μg/L	4	[57]
Botulinum neurotoxin type B	50 ng/mL	50 pg/mL	1000	[58]
Botulinum neurotoxin type A	50 ng/mL	1 ng/mL	50	[59]
Fumonisin B1	5 ng/mL (cut-off value)	2 ng/mL (cut-off value)	2.5	[60]
Deoxynivalenol	80 ng/mL (cut-off value)	40 ng/mL (cut-off value)	2	

Helicobacter pylori cell wall antigens	0.4 µg/mL (Ag 0100)<br>0.5 µg/mL (Ag 0103, 0104)	0.03 µg/mL (Ag 0100)<br>0.05 µg/mL (Ag 0103, 0104)	13<br>10	[61]
Prostate-specific antigen	0.5 ng/mL	0.1 ng/mL	5	[62]
Ralstonia solanacearum	2 × 10^3 CFU/mL	2 × 10^2 CFU/mL	10	[63]
Cortisol	1.8 ng/mL	0.5 ng/mL	3.6	[64]
Cardiac troponin I	10 ng/mL	0.24 ng/mL	42	[65]
Potato leafroll virus	3 ng/mL	0.2 ng/mL	15	[66]
Staphylococcal *e*nterotoxin B	10 ng/mL	0.1 ng/mL	100	[67]
Interleukin-6	50 pg/mL (buffer)<br>200 pg/mL (serum)	1 pg/mL (serum)<br>50 pg/mL (serum)	50<br>4	[68]
Cardiac troponin I	25 ng/mL	0.5 ng/mL	50	[69]
Microcystin-LR	0.2 ng/mL	0.05 ng/mL	4	[70]
Staphylococcal enterotoxin B	1 ng/mL	10 pg/mL	100	[71]
Ricin	50 ng/mL	100 pg/mL	500	[72]
Hg	0.5 ppb	0.0005	1000	[73]
miRNA	1 fmol	5 amol	200	[74]
Sodium pentachlorophenate	10 ng/mL	4 ng/mL	2.5	[75]
E. coli O157:H7	2 × 10^6 CFU/mL	2 × 10^3 CFU/mL	1000	[76]
		**Gold enhancement**		
Mycobacterium tuberculosis antigen 85B	1.57	0.93 ng/mL	1.68	[45]
Receptor binding domain of SARS-CoV-2	488 pg/mL	1 pg/mL	488	[50]
E. coli O157:H7	5 × 10^3 CFU/mL	1.25 × 10^2 CFU/mL	40	[77]
Vegetative insecticidal protein S	100 ng/mL	1 ng/mL	100	[78]
Human chorionic gonadotropin	1.2±0.5 mIU/mL	0.3±0.2 mIU/mL	4 (reported by the authors)	[79]
Plasmodium falciparum histidine rich protein 2 (PfHRP2)	10.4±4.4 ng/mL	2.9±1.2 ng/mL	4 (reported by the authors)	[80]
Avian Influenza virus<br>Newcastle disease virus	2^{-2} dilution<br>2^{-4} dilution (commercial test strip)<br>2^{-2} dilution	2^{-10} dilution<br>2^{-12} dilution (commercial; test strip)<br>2^{-12} dilution	256<br>256<br>1024	[81]
E. coli O157:H7	4 × 10^4 CFU/mL	5 × 10^3 CFU/mL	8	[82]

Salmonella typhimurium	10^7 CFU/mL	10^6 CFU/mL	10	[83]
Norovirus	10^7 copies/mL	9.5×10^4 copies/mL	105	[84]
Protein G molecules	2×10^5 molecules/spot for GNP<br>2×10^4 molecules/spot for Ag nanoparticles<br>2×10^7 molecules/spot for magnetic nanoparticles	2×10^3 molecules/spot for GNP<br>2×10^2 molecules/spot for Ag nanoparticles<br>2×10^5 molecules/spot for magnetic nanoparticles	100<br>100<br>100	[85]
Salmonella enteritidis	10^6 CFU/mL	10^4 CFU/mL	100	[86]
Potato virus X	4.1 ng/mL	17 pg/mL	240	[87]
Ralstonia solanacearum	1×10^6 cells/mL (buffer)<br>1×10^6 cells/mL (potato tuber extract)	2×10^4 cells/mL (buffer)<br>3×10^4 cells/mL (potato tuber extract)	50<br>33	[88]
Cardiac troponin I	500 pg/mL	0.92 pg/mL	543	[89]
Staphylococcus aureus	10^5 CFU/mL	10^3 CFU/mL	100	[90]
C-reactive protein	0.5 µg/mL	0.1 µg/mL	5	[91]
Sugarcane streak mosaic virus protein	1 ng/mL	10 pg/mL	100	[92]
Ferritin	10 ng/mL	0.5 ng/mL	20	[93]
Hepatitis B surface antigen<br>P24 of HIV	9.75 ng/mL<br>900 pg/mL	0.0198 ng/mL<br>12.5 pg/mL	492<br>72	[94]<br>[94]
Fatty acid-binding protein	0.1 ng/mL	0.05 ng/mL	2	[95]
N protein of SARS-CoV-2	5 ng/mL	0.5 ng/mL	10	[96]
Short DNA sequence	20 pM	0.2 pM	100	[97]
Atrazine	1:800 dilution	1:3200 dilution	4	[98]
Cortisol	1 ng/mL	0.1 ng/mL	10	[99]
Cardiac troponin I-C-T	1 ng/mL	0.1 ng/mL	10	[100]
Hg	0.5 ppb	0.01	50	[73]
Glutamate dehydrogenase, toxin A, and toxin B of *Clostridioides difficile*		0.16 ng/mL<br>0.09 ng/mL<br>0.03 ng/mL	5-8	[101]
		**Enzyme**		
Myoglobin	1 ug/mL	10 ng/mL	100	[102]

Human IgG	30.5 ± 1.9 ng/mL;	8.2 ± 1.2 ng/mL<br>18.2 ± 0.9 ng/mL	4	[103]
DNA		0.025 ng/mL	1000	[104]
E. coli	10^5 CFU/mL	100 CFU/mL	1000	[105]
C-reactive protein	13.65 ng/mL	0.03 ng/mL	455	[106]
Hepatitis B	3.6 ng/mL	0.12 ng/mL	30	[107]
Nucleic acids			5	[108]
Listeria monocytogenes, *Escherichia coli* O157:H7 and *Yersinia enterocolitica*			5-10	[109]
Human IgG	50	5	10	[110]
Cardiac troponin I	216 pg/mL	0.84 pg/mL	257	[111]
Cardiac troponin I	0.82 ng/mL	0.027 ng/mL	30	[112]
a-fetoprotein	5 ng/mL	0.27 ng/mL	18.5	[113]
DNA	65 fmol	1.25 fmol	52	[114]
CRP	13.65	0.03	455	[106]
Microcystin-leucine-arginine	550 pg/mL	50 pg/mL	11	[115]
Japanese cedar pollen		4 IU/mL	20	[116]
Influenza viruses	Different LODs for different strains		10-40	[117]
Potato virus X	8	0.3 ng/mL	27	[118]
Carcinoembryonic antigen<br>Alpha fetoprotein<br>Procalcitonin	5 ng/mL<br>5 ng/mL<br>10 pg/mL	0.2 ng/mL<br>0.21 ng/mL<br>0.02 pg/mL	25<br>24<br>500	[119]
Cardiac troponin I	607 pg/mL	30 pg/mL	20.2	[120]
Human IgG	36.4 ng/mL	8.2 ng/mL	4	[103]
microRNA-224	75 pm	7.5 pM	10	[121]
Carbaryl<br>Endosulfan	100 ug/L<br>10 ug/L	10 ug/L<br>1 ug/L	10<br>10	[122]
8-hydroxy-2’-deoxyguanosine	2 ng/mL	0.23 ng/mL	8.7	[123]
P24	10 ng/mL	10 pg/mL	1000	[124]
Dengue virus	DENV-1 (2.5 ng/mL)	DENV-1 (0.25 ng/mL)<br>DENV-2 (0.1 ng/mL)	10	[125]

	DENV-2 (1 ng/mL)	DENV-3 (0.25 ng/mL)	10	
	DENV-3 (2.5 ng/mL)	DENV-4 (1 ng/mL)	10	
	DENV-4 (10 ng/mL)		10	
		**Non-classified**		
Receptor binding domain of SARS-CoV-2	488 pg/mL	8 pg/mL	61	[50]
Okadaic acid	0.2 ng/mL	0.03 ng/mL	6.7	[126]
Carcinoembryonic antigen	0.5 ng/mL	0.1 fg/mL	5×10^6	[127]
HIV-1 capsid p24	0.5 ng/mL	0.01 fg/mL	5×10^7	[127]
Salbutamol				[128]
		**Nanozyme**		
3-phenoxybenzoic acid	0.1 ng/mL	0.005 ng/mL	20	[129]
Salmonella enteritidis	10^6 CFU/mL	20 CFU/mL	50000	[130]
E. coli 0157:H7		34 CFU/mL	29411	
Human chorionic gonadotropin	3.7 ng/mL (GNP)	0.025 ng/mL	148	[32]
P24		0.8 pg/mL	100	[131]
Carcinoembryonic antigen`	1.26 ng/mL (GNP)	7.8 pg/mL (Au@Ir)<br>27.5 pg/mL (Au@Pt)	161	[132]
Prostate-specific antigen	0.41 ng/mL (GNP)	2.2. pg/mL	186	
Procalcitonin	0.068 ng/mL	0.0005 ng/mL	136	[133]
P53	100 ng/mL	0.05 ng/mL	2000	[134]
Dehydroepiandrosterone	0.43ng/mL	0.032ng/mL	13.4	[135]
Dehydroepiandrosterone	100 ng/mL	50 ng/mL	2	[135]
Gastrin-17	110 pg/mL	10 pg/mL	11	[136]
Tobramycin	0.09 nM	0.02 nM	4.5	[137]
N-protein of SARS-CoV-2	1 ng/mL	0.1 ng/mL	10	[138]
Stremtomycin	0.9 ng/mL	0.06 ng/mL	15	[139]
Plasmodium lactate dehydrogenase	10 ng/mL	0.1 ng/mL	100	[140]
Chlorpyrifos	0.1 ng/mL	0.033 ng/mL	3	[141]
E. coli	3.3×10^5 CFU/mL	3.3×10^4 CFU/mL	10	[142]

N-protein of SARS-CoV-2	0.632 ng/mL	0.037 ng/mL	17	[143]
Human chorionic gonadotropin	8 mIU/mL	0.05 mIU/mL	160	[144]
Extracellular vesicles	5.73×10^7 vesicles/uL	2.49×10^7 vesicles/uL	2.3	[145]
Okadaic acid	1.5 ng/mL	0.5 ng/mL	3	[146]
E. coli O157:H7<br>*Salmonella typhimurium*	10^4<br>5×10^3	10^3<br>5×10^2	10<br>10	[147]
Cardiac troponin I	2 ng/mL	20 pg/mL	100	[148]
Aspergillus flavus mycelium biomass	8.7 ng/mL	0.45 ng/mL	19.3	[149]
Ractopamine<br>Clenbuterol	1 ng/mL<br>1 ng/mL	0.12 ng/mL<br>0.2 ng/mL	8.3<br>5	[150]
Acetochlor<br>Fenpropathrin	1 ng/mL	0.1 ng/mL	10	[151]
E. coli O157:H7	100 CFU/mL	10 CFU/mL	10	[152]
SARS-CoV-2 spike antigen	25 ng/mL	0.1 ng/mL	250	[153]
Glycoprotein of Ebola virus	10 ng/mL	1 ng/mL	10	[154]
Ractopamine<br>Clenbuterol	0.3 ng/mL<br>4 ng/mL	0.2 ng/mL<br>2 ng/mL	1.5<br>2	[155]
Furazolidone	1 ng/mL	0.4 ng/mL	2.5	[156]
Aflatoxin B_1	0.109 ng/mL	0.015 ng/mL	7.3	[157]
Aflatoxin B1	90 pg/mL	23 pg/mL	3.5	[158]
E. coli O157:H7	1.25×10^2 CFU/mL	1.25×10^1 CFU/mL	10	[77]
E. coli O157:H7	1×10^4 CFU/mL (buffer)<br>5×10^4 CFU/mL (milk)	1×10^2 CFU/mL (buffer)<br>1×10^3 CFU/mL (milk)	100<br>50	[159]
Aflatoxin B1<br>Fumonisin B1	44.5 pg/mL<br>157 pg/mL	2.8 pg/mL<br>13.9 pg/mL	15.9<br>11.3	[160]
Human chorionic gonadotropin	25 mIU/mL	15 mIU/mL	1.7	[161]
Clavibacter michiganensis	10^5 CFU/mL (low Pt concentration)<br>5×10^4 CFU/mL (high Pt concentration)	10^4 CFU/mL (low platinum concentration)<br>3×10^2 CFU/mL (high Pt concentration)	10<br>167	[162]

C-reactive protein	980 pg/mL	15 pg/mL	65	[163]
Rabbit IgG	47 pg/mL	5 pg/mL	9.4	[164]
Prostate-specific antigen	272 pg/mL	3.1 pg/mL	87.7	[165]
Dichlorophenoxyacetic acid (2,4-D)	1.54 ng/mL (buffer)<br>1.82 ng/mL (urine)	0.82 ng/mL (urine)<br>0.93 ng/mL (urine)	1.87<br>1.95	[166]
Staphylococcal enterotoxin B	250 pg/mL	1.2 pg/mL	208	[167]
Rabbit IgG	10 ng/mL	10 pg/mL	1000	[168]
Potato virus X	500 pg/mL	4 pg/mL	125	[169]
DNA	4.45 pM	0.43 pM	10	[170]
C-reactive protein	19.75 ng/mL	0.32 ng/mL	62	[171]
MicroRNA-21	50 pM	0.3 pM	167	[172]
SARS-CoV-2 nucleic acid copies	1000 copies/mL	200 copies/mL	5	[173]
Salmonella typhimurium	10^4 CFU/mL	10^3 CFU/mL	10	[174]
Staphylococcus aureus	10^3 CFU/mL	18 CFU/mL	55	[175]

Table S3. Time of various enhancement methods.

Antigen	Time, min	References
**Copper enhancement**		
CFP-10 antigen	10	[44]
Mycobacterium tuberculosis antigen 85B (Ag85B)	5	[45]
IgG and prostate-specific antigen	6	[176]
Bovine viral diarrhea virus	10	[49]
GlcNAc- conjugated BSA	6	[177]
Nucleocapsid protein of SARS-CoV-2	3	[48]
Amyloid β Oligomers 42	10	[178]
Anthrax DNA<br>Norovirus	10	[179]
E. coli O157:H7	15	[46]
HIV-1 capsid p24 antigen<br>*E. coli* O157:H7	7	[43]
Human chorionic gonadotropin<br>Rabbit IgG	10	[47]
**Silver enhancement**		
CFP-10 antigen	10	[44]
HCG<br>Rabbit IgG	10	[47]
15 various proteins	15	[180]
Plasmodium falciparum histidine-rich protein 2	25	[181]
Pesticides	15	[182]
RBD of SARS-CoV-2	2	[50]
Botulinum neurotoxin type B	5	[58]
Nucleotides	5	[183]
IgG	10	[184]
Botulinum neurotoxin type A	5	[59]
Prostate-specific antigen	5	[185]
Cardiac troponin I	7	[51]
Abrin-a	10	[53]
Potato virus X	10	[54]
Ochratoxin A	10	[56]

Salmonella nucleic acid	7	[55]
Cadmium	10	[57]
Helicobacter pylori antigens	10	[61]
Fumonisin B_1<br>Deoxynivalenol	6	[60]
Prostate-specific antigen	10	[62]
Ralstonia solanacearum	10	[63]
Potato leafroll virus	5	[66]
Interleukin-6	10 (buffer)<br>30 (serum)	[68]
Microcystin-LR	10	[70]
Cardiac troponin I	15	[69]
Cortisol	5	[64]
Staphylococcal enterotoxin B	5	[67]
Staphylococcal enterotoxin B	5	[71]
Ricin	8	[72]
Hg	10	[73]
miRNA	15	[74]
Sodium pentachlorophenate	5	[75]
E. coli O157:H7	4	[76]
**Gold enhancement**		
Mycobacterium tuberculosis antigen 85B (Ag85B)	3	[45]
IgG	5	[186]
Atrazine	2	[98]
Vip-S protein	2	[78]
Avian influenza virus<br>Newcastle disease virus	5	[81]
E. coli O157:H7	5	[82]
Pesticides	10	[182]
DNA sequences	2	[97]
Fatty acid binding protein	1	[95]
Sugarcane mosaic viruses	2	[92]
HBsAg<br>P24	10	[94]

Staphylococcus aureus	5	[90]
RBD of SARS-CoV-2	3	[50]
E. coli O157:H7	2	[77]
Ralstonia solanacearum	3	[88]
Potato virus X	1	[87]
E. coli O157:H7, *Salmonella typhimurium*, *Staphylococcus aureus, Bacillus cereus*	3	[187]
Salmonella enteritidis	10	[86]
Protein G	5	[85]
RBD of SARS-CoV-2	3	[50]
Cardiac troponin I-C-T	5	[100]
Hg	3	[73]
**Enzyme**		
Monensin	10-20	[188]
IgE specific for Japanese cedar pollen	20	[116]
Hepatitis B surface antigen	5	[107]
Cardiac troponin I	5	[189]
DNA	5	[114]
Cardiac troponin I	0.5	[112]
IgG	2	[190]
DNA	6	[104]
Activity of acetylcholinesterase	2	[191]
Trypanosoma mRNA sequences	20-30	[192]
Progesterone	5	[193]
IgG	5	[110]
Listeria monocytogenes	5	[194]
C-reactive protein	10	[195]
Cortisol	<1	[196]
Progesterone	5	[197]
Aflatoxin B1<br>Fumonisin B1	4	[198]
Cardiac troponin I	5	[199]

C-reactive protein	20	[106]
MicroRNA-224	9	[121]
E. coli 0157:H7<br>*Salmonella typhimurium*	5	[200]
E. coli 0157:H7	4	[201]
Alpha fetoprotein<br>Carcinoembryonic antigen	1	[119]
Human serum albumin	2	[202]
Plasmodium falciparum histidine-rich protein 2	8<br>10<br>25	[181]
Plasmodium falciparum HRP	5	[203]
α-fetoprotein<br>Folic acid	1	[113]
Potato virus X	5	[118]
Cortisol	15	[204]
Cardiac troponin I	6	[111]
Nucleic acid sequence	15	[108]
Cardiac troponin I	5	[120]
Cortisol	5	[205]
Ochratoxin A	15	[206]
Microcystin-LR	2	[115]
α-fetoprotein<br>Carcinoembryonic antigen	3	[207]
Lipoarabinomannan	10	[208]
DNA sequence	3	[209]
IgG	10	[103]
P24	10	[124]
C-reactive protein	20	[106]
Dengue virus	10	[125]
**Nanozymes**		
Ebola virus	7	[154]
P53	10	[134]
E. coli O157:H7	10	[210]

Human chorionic gonadotropin	5	[32]
Salmonella enteritidis<br>*E. coli* O157:H7	10	[130]
DNA of *Enterobacter sakazakii*	5	[211]
Prostate-specific antigen	5	[165]
Butyrylcholinesterase activity	5	[212]
P24	5	[131]
Myoglobin	10	[213]
E. coli O157:H7	4 in buffer<br>8 in milk	[159]
Acetochlor<br>Fenpropathrin	10	[151]
Myoglobin<br>Human chorionic gonadotropin<br>Luteinizing hormone	30	[214]
Human chorionic gonadotropin	4	[215]
Rabbit IgG	5	[164]
Cancer antigen 125	10	[216]
Okadaic acid	2	[146]
Protein A	5	[217]
2,4- dichlorophenoxyacetic acid	10	[166]
3-Phenoxybenzoic Acid	5	[129]
Human cardiac troponin I	5	[148]
E. coli O157:H7	15	[152]
17β-estradiol	7	[218]
Procalcitonin	10	[133]
C-reactive protein	3	[219]
Human chorionic gonadotropin	15	[161]
Carcinoembryonic antigen Prostate-specific antigen	2	[132]
Gastrin-17	10	[136]
Extracellular vesicles	15	[145]
Aflatoxin B1	9	[157]
N-protein SARS-CoV-2	15	[143]
Hepatitis B virus DNA	10	[220]

Staphylococcal enterotoxin B	5	[167]
Salmonella typhimurium<br>*E. coli* O157:H7	0.5	[147]
Human chorionic gonadotropin	10	[144]
Amyloid β protein	5	[221]
Fumonisin B<br>Aflatoxin B1	1	[160]
Salbutamol	2	[222]
DNA	5	[170]
C-reactive protein	0.5	[171]
MicroRNA-21	5	[172]
SARS-CoV-2 nucleic acid copies	6	[173]
Salmonella typhimurium	5	[174]

References

1. Gao, L.; Zhuang, J.; Nie, L.; Zhang, J.; Zhang, Y.; Gu, N.; Wang, T.; Feng, J.; Yang, D.; Perrett, S.; et al. Intrinsic Peroxidase-like Activity of Ferromagnetic Nanoparticles. *Nat. Nanotechnol.* **2007**, *2*, 577–583, doi:10.1038/nnano.2007.260.
2. Wang, X.-X.; Wu, Q.; Shan, Z.; Huang, Q.-M. BSA-Stabilized Au Clusters as Peroxidase Mimetics for Use in Xanthine Detection. *Biosens. Bioelectron.* **2011**, *26*, 3614–3619, doi:10.1016/j.bios.2011.02.014.
3. Drozd, M.; Pietrzak, M.; Parzuchowski, P.; Mazurkiewicz-Pawlicka, M.; Malinowska, E. Peroxidase-like Activity of Gold Nanoparticles Stabilized by Hyperbranched Polyglycidol Derivatives over a Wide PH Range. *Nanotechnology* **2015**, *26*, doi:10.1088/0957-4484/26/49/495101.
4. Hu, D.; Sheng, Z.; Fang, S.; Wang, Y.; Gao, D.; Zhang, P.; Gong, P.; Ma, Y.; Cai, L. Folate Receptor-Targeting Gold Nanoclusters as Fluorescence Enzyme Mimetic Nanoprobes for Tumor Molecular Colocalization Diagnosis. *Theranostics* **2014**, *4*, 142–153, doi:10.7150/thno.7266.
5. Tao, Y.; Lin, Y.; Huang, Z.; Ren, J.; Qu, X. Incorporating Graphene Oxide and Gold Nanoclusters: A Synergistic Catalyst with Surprisingly High Peroxidase-like Activity over a Broad PH Range and Its Application for Cancer Cell Detection. *Adv. Mater.* **2013**, *25*, 2594–2599, doi:10.1002/adma.201204419.
6. Männel, M.J.; Kreuzer, L.P.; Goldhahn, C.; Schubert, J.; Hartl, M.J.; Chanana, M. Catalytically Active Protein Coatings: Toward Enzymatic Cascade Reactions at the Intercolloidal Level. *ACS Catal.* **2017**, *7*, 1664–1672, doi:10.1021/acscatal.6b03072.
7. Ye, H.; Yang, K.; Tao, J.; Liu, Y.; Zhang, Q.; Habibi, S.; Nie, Z.; Xia, X. An Enzyme-Free Signal Amplification Technique for Ultrasensitive Colorimetric Assay of Disease Biomarkers. *ACS Nano* **2017**, *11*, 2052–2059, doi:10.1021/acsnano.6b08232.
8. Jiang, X.; Sun, C.; Guo, Y.; Nie, G.; Xu, L. Peroxidase-like Activity of Apoferritin Paired Gold Clusters for Glucose Detection. *Biosens. Bioelectron.* **2015**, *64*, 165–170, doi:10.1016/j.bios.2014.08.078.
9. Mu, J.; Wang, Y.; Zhao, M.; Zhang, L. Intrinsic Peroxidase-like Activity and Catalase-like Activity of Co3O4 Nanoparticles. *Chem. Commun.* **2012**, *48*, 2540, doi:10.1039/c2cc17013b.
10. Lou, D.; Tian, Y.; Zhang, Y.; Yin, J.; Yang, T.; He, C.; Ma, M.; Yu, W.; Gu, N. Peroxidase-Like Activity of Gold Nanoparticles and Their Gold Staining Enhanced ELISA Application. *J. Nanosci. Nanotechnol.* **2017**, *18*, 951–958, doi:10.1166/jnn.2018.13977.
11. Wei, J.; Chen, X.; Shi, S.; Mo, S.; Zheng, N. An Investigation of the Mimetic Enzyme Activity of Two-Dimensional Pd-Based Nanostructures. *Nanoscale* **2015**, *7*, 19018–19026, doi:10.1039/c5nr05675f.
12. Gao, Z.; Xu, M.; Hou, L.; Chen, G.; Tang, D. Irregular-Shaped Platinum Nanoparticles as Peroxidase Mimics for Highly Efficient Colorimetric Immunoassay. *Anal. Chim. Acta* **2013**, *776*, 79–86, doi:10.1016/j.aca.2013.03.034.
13. Zhang, X.Q.; Gong, S.W.; Zhang, Y.; Yang, T.; Wang, C.Y.; Gu, N. Prussian Blue Modified Iron Oxide Magnetic Nanoparticles and Their High Peroxidase-like Activity. *J. Mater. Chem.* **2010**, *20*, 5110–5116, doi:10.1039/c0jm00174k.
14. Gao, Z.; Lv, S.; Xu, M.; Tang, D. High-Index {: Hk 0} Faceted Platinum Concave Nanocubes with Enhanced Peroxidase-like Activity for an Ultrasensitive Colorimetric Immunoassay of the Human Prostate-Specific Antigen. *Analyst* **2017**, *142*, 911–917, doi:10.1039/c6an02722a.
15. Ye, H.; Liu, Y.; Chhabra, A.; Lilla, E.; Xia, X. Polyvinylpyrrolidone (PVP)-Capped Pt Nanocubes with Superior Peroxidase-like Activity. *ChemNanoMat* **2017**, *3*, 33–38, doi:10.1002/cnma.201600268.
16. Choleva, T.G.; Gatselou, V.A.; Tsogas, G.Z.; Giokas, D.L. Intrinsic Peroxidase-like Activity of Rhodium Nanoparticles, and Their Application to the Colorimetric Determination of Hydrogen Peroxide and Glucose. *Microchim. Acta* **2018**, *185*, doi:10.1007/s00604-017-2582-8.
17. Liu, J.; Hu, X.; Hou, S.; Wen, T.; Liu, W.; Zhu, X.; Yin, J.J.; Wu, X. Au@Pt Core/Shell Nanorods with Peroxidase- and Ascorbate Oxidase-like Activities for Improved Detection of Glucose. *Sensors Actuators, B Chem.* **2012**, *166–167*, 708–714, doi:10.1016/j.snb.2012.03.045.
18. He, W.; Liu, Y.; Yuan, J.; Yin, J.J.; Wu, X.; Hu, X.; Zhang, K.; Liu, J.; Chen, C.; Ji, Y.; et al. Au@Pt Nanostructures as Oxidase and Peroxidase Mimetics for Use in Immunoassays. *Biomaterials* **2011**, *32*, 1139–1147, doi:10.1016/j.biomaterials.2010.09.040.
19. Hu, X.; Saran, A.; Hou, S.; Wen, T.; Ji, Y.; Liu, W.; Zhang, H.; Wu, X. Rod-Shaped Au@PtCu Nanostructures with Enhanced Peroxidase-like Activity and Their ELISA Application. *Chinese Sci. Bull.* **2014**, *59*, 2588–2596, doi:10.1007/s11434-014-0316-4.
20. Hu, X.; Saran, A.; Hou, S.; Wen, T.; Ji, Y.; Liu, W.; Zhang, H.; He, W.; Yin, J.J.; Wu, X. Au@PtAg Core/Shell Nanorods: Tailoring Enzyme-like Activities via Alloying. *RSC Adv.* **2013**, *3*, 6095–6105, doi:10.1039/c3ra23215h.
21. Xia, X.; Zhang, J.; Lu, N.; Kim, M.J.; Ghale, K.; Xu, Y.; McKenzie, E.; Liu, J.; Ye, H. Pd-Ir Core-Shell Nanocubes: A Type of Highly Efficient and Versatile Peroxidase Mimic. *ACS Nano* **2015**, *9*, 9994–10004, doi:10.1021/acsnano.5b03525.
22. Jia, H.; Yang, D.; Han, X.; Cai, J.; Liu, H.; He, W. Peroxidase-like Activity of the Co3O4 Nanoparticles Used for Biodetection and Evaluation of Antioxidant Behavior. *Nanoscale* **2016**, *8*, 5938–5945, doi:10.1039/c6nr00860g.
23. Gao, T.; Mu, C.; Shi, H.; Shi, L.; Mao, X.; Li, G. Embedding Capture-Magneto-Catalytic Activity into a Nanocatalyst for the Determination of Lipid Kinase. *ACS Appl. Mater. Interfaces* **2018**, *10*, 59–65, doi:10.1021/acsami.7b10857.
24. Fan, H.; Yi, J.; Yang, Y.; Kho, K.; Tan, H.-R.; Shen, Z.; Ding, J.; Sun, X.; Olivo, M.C.; Feng, Y. Single-Crystalline MFe 2 O 4 Nanotubes/Nanorings Synthesized by Thermal Transformation Process for Biological Applications. *ACS Nano* **2009**, *3*, 2798–2808, doi:10.1021/nn9006797.
25. Bhattacharya, D.; Baksi, A.; Banerjee, I.; Ananthakrishnan, R.; Maiti, T.K.; Pramanik, P. Development of Phosphonate Modified Fe(1-x)MnxFe 2O4 Mixed Ferrite Nanoparticles: Novel Peroxidase Mimetics in Enzyme Linked Immunosorbent Assay. *Talanta* **2011**, *86*, 337–348, doi:10.1016/j.talanta.2011.09.026.
26. Liu, X.; Wang, Q.; Zhao, H.; Zhang, L.; Su, Y.; Lv, Y. BSA-Templated MnO2 Nanoparticles as Both Peroxidase and Oxidase Mimics. *Analyst* **2012**, *137*, 4552–4558, doi:10.1039/c2an35700c.

27. Wan, Y.; Qi, P.; Zhang, D.; Wu, J.; Wang, Y. Manganese Oxide Nanowire-Mediated Enzyme-Linked Immunosorbent Assay. *Biosens. Bioelectron.* **2012**, *33*, 69–74, doi:10.1016/j.bios.2011.12.033.
28. Deng, H.; Shen, W.; Peng, Y.; Chen, X.; Yi, G.; Gao, Z. Nanoparticulate Peroxidase/Catalase Mimetic and Its Application. *Chem. - A Eur. J.* **2012**, *18*, 8906–8911, doi:10.1002/chem.201200643.
29. Yu, J.; Ma, D.; Mei, L.; Gao, Q.; Yin, W.; Zhang, X.; Yan, L.; Gu, Z.; Ma, X.; Zhao, Y. Peroxidase-like Activity of MoS2 Nanoflakes with Different Modifications and Their Application for H2O2 and Glucose Detection. *J. Mater. Chem. B* **2018**, *6*, 487–498, doi:10.1039/c7tb02676e.
30. Zhang, W.; Hu, S.; Yin, J.J.; He, W.; Lu, W.; Ma, M.; Gu, N.; Zhang, Y. Prussian Blue Nanoparticles as Multienzyme Mimetics and Reactive Oxygen Species Scavengers. *J. Am. Chem. Soc.* **2016**, *138*, 5860–5865, doi:10.1021/jacs.5b12070.
31. Su, L.; Xiong, Y.; Yang, H.; Zhang, P.; Ye, F. Prussian Blue Nanoparticles Encapsulated inside a Metal-Organic Framework via in Situ Growth as Promising Peroxidase Mimetics for Enzyme Inhibitor Screening. *J. Mater. Chem. B* **2016**, *4*, 128–134, doi:10.1039/c5tb01924a.
32. Kim, M.S.; Kweon, S.H.; Cho, S.; An, S.S.A.; Kim, M. Il; Doh, J.; Lee, J. Pt-Decorated Magnetic Nanozymes for Facile and Sensitive Point-of-Care Bioassay. *ACS Appl. Mater. Interfaces* **2017**, *9*, 35133–35140, doi:10.1021/acsami.7b12326.
33. Wang, W.; Du, R.; Dong, C.; Yan, J.; Zhang, L. A Modified Sensitive ELISA Based on Dual Catalysis of Pd@Pt Porous Nanoparticles and Horseradish Peroxidase. *Sensors Actuators, B Chem.* **2019**, *284*, 475–484, doi:10.1016/j.snb.2019.01.002.
34. Wei, D.; Zhang, X.; Chen, B.; Zeng, K. Using Bimetallic Au@Pt Nanozymes as a Visual Tag and as an Enzyme Mimic in Enhanced Sensitive Lateral-Flow Immunoassays: Application for the Detection of Streptomycin. *Anal. Chim. Acta* **2020**, *1126*, 106–113, doi:10.1016/j.aca.2020.06.009.
35. Singh, S.; Tripathi, P.; Kumar, N.; Nara, S. Colorimetric Sensing of Malathion Using Palladium-Gold Bimetallic Nanozyme. *Biosens. Bioelectron.* **2017**, *92*, 280–286, doi:10.1016/j.bios.2016.11.011.
36. Khoris, I.M.; Takemura, K.; Lee, J.; Hara, T.; Abe, F.; Suzuki, T.; Park, E.Y. Enhanced Colorimetric Detection of Norovirus Using In-Situ Growth of Ag Shell on Au NPs. *Biosens. Bioelectron.* **2019**, *126*, 425–432, doi:10.1016/j.bios.2018.10.067.
37. Komkova, M.A.; Karyakina, E.E.; Karyakin, A.A. Catalytically Synthesized Prussian Blue Nanoparticles Defeating Natural Enzyme Peroxidase. *J. Am. Chem. Soc.* **2018**, *140*, 11302–11307, doi:10.1021/jacs.8b05223.
38. Wei, Z.; Luciano, K.; Xia, X. Catalytic Gold-Iridium Nanoparticles as Labels for Sensitive Colorimetric Lateral Flow Assay. *ACS Nano* **2022**, *16*, 21609–21617, doi:10.1021/acsnano.2c10574.
39. Xi, Z.; Gao, W.; Biby, A.; Floyd, A.; Xia, X. Ultrasmall Iridium Nanoparticles as Efficient Peroxidase Mimics for Colorimetric Bioassays. *ACS Appl. Nano Mater.* **2022**, *5*, 6089–6093, doi:10.1021/acsanm.2c01226.
40. Xi, Z.; Wei, K.; Wang, Q.; Kim, M.J.; Sun, S.; Fung, V.; Xia, X. Nickel–Platinum Nanoparticles as Peroxidase Mimics with a Record High Catalytic Efficiency. *J. Am. Chem. Soc.* **2021**, *143*, 2660–2664, doi:10.1021/jacs.0c12605.
41. Xi, Z.; Gao, W.; Xia, X. Size Effect in Pd−Ir Core-Shell Nanoparticles as Nanozymes. *ChemBioChem* **2020**, *21*, 2440–2444, doi:10.1002/cbic.202000147.
42. Wu, J.; Wang, X.; Wang, Q.; Lou, Z.; Li, S.; Zhu, Y.; Qin, L.; Wei, H. Nanomaterials with Enzyme-like Characteristics (Nanozymes): Next-Generation Artificial Enzymes (II). *Chem. Soc. Rev.* **2019**, *48*, 1004–1076, doi:10.1039/C8CS00457A.
43. Zhou, Y.; Chen, Y.; Liu, Y.; Fang, H.; Huang, X.; Leng, Y.; Liu, Z.; Hou, L.; Zhang, W.; Lai, W.; et al. Controlled Copper in Situ Growth-Amplified Lateral Flow Sensors for Sensitive, Reliable, and Field-Deployable Infectious Disease Diagnostics. *Biosens. Bioelectron.* **2021**, *171*, 112753, doi:10.1016/j.bios.2020.112753.
44. Phan, L.M.T.; Rafique, R.; Baek, S.H.; Nguyen, T.P.; Park, K.Y.; Kim, E.B.; Kim, J.G.; Park, J.P.; Kailasa, S.K.; Kim, H.-J.; et al. Gold-Copper Nanoshell Dot-Blot Immunoassay for Naked-Eye Sensitive Detection of Tuberculosis Specific CFP-10 Antigen. *Biosens. Bioelectron.* **2018**, *121*, 111–117, doi:10.1016/j.bios.2018.08.068.
45. Phan, L.M.T.; Kim, E.B.; Cheon, S.A.; Shim, T.S.; Kim, H.J.; Park, T.J. Reliable Naked-Eye Detection of Mycobacterium Tuberculosis Antigen 85B Using Gold and Copper Nanoshell-Enhanced Immunoblotting Techniques. *Sensors Actuators, B Chem.* **2020**, *317*, 128220, doi:10.1016/j.snb.2020.128220.
46. Shao, Y.; Xu, W.; Zheng, Y.; Wang, J.; Xie, J.; Zhu, Z.; Xiang, X.; Ye, Q.; Zhang, Y.; Xue, L.; et al. Controlled PAH-Mediated Method with Enhanced Optical Properties for Simple, Stable Immunochromatographic Assays. *Biosens. Bioelectron.* **2022**, *206*, 114150, doi:10.1016/j.bios.2022.114150.
47. Tian, M.; Lei, L.; Xie, W.; Yang, Q.; Li, C.M.; Liu, Y. Copper Deposition-Induced Efficient Signal Amplification for Ultrasensitive Lateral Flow Immunoassay. *Sensors Actuators B Chem.* **2019**, *282*, 96–103, doi:10.1016/j.snb.2018.11.028.
48. Peng, T.; Jiao, X.; Liang, Z.; Zhao, H.; Zhao, Y.; Xie, J.; Jiang, Y.; Yu, X.; Fang, X.; Dai, X. Lateral Flow Immunoassay Coupled with Copper Enhancement for Rapid and Sensitive SARS-CoV-2 Nucleocapsid Protein Detection. *Biosensors* **2021**, *12*, 13, doi:10.3390/bios12010013.
49. Kim, M.W.; Park, H.-J.; Park, C.Y.; Kim, J.H.; Cho, C.H.; Phan, L.M.T.; Park, J.P.; Kailasa, S.K.; Lee, C.-H.; Park, T.J. Fabrication of a Paper Strip for Facile and Rapid Detection of Bovine Viral Diarrhea Virus via Signal Enhancement by Copper Polyhedral Nanoshells. *RSC Adv.* **2020**, *10*, 29759–29764, doi:10.1039/D0RA03677C.
50. Panferov, V.G.; Byzova, N.A.; Biketov, S.F.; Zherdev, A. V; Dzantiev, B.B. Comparative Study of In Situ Techniques to Enlarge Gold Nanoparticles for Highly Sensitive Lateral Flow Immunoassay of SARS-CoV-2. *Biosensors* **2021**, *11*, 229, doi:10.3390/bios11070229.
51. Cho, I.H.; Seo, S.M.; Paek, E.H.; Paek, S.H. Immunogold-Silver Staining-on-a-Chip Biosensor Based on Cross-Flow Chromatography. *J. Chromatogr. B Anal. Technol. Biomed. Life Sci.* **2010**, *878*, 271–277, doi:10.1016/j.jchromb.2009.07.016.
52. Wada, A.; Sakoda, Y.; Oyamada, T.; Kida, H. Development of a Highly Sensitive Immunochromatographic Detection Kit for H5 Influenza Virus Hemagglutinin Using Silver Amplification. *J. Virol. Methods* **2011**, *178*, 82–86, doi:10.1016/j.jviromet.2011.08.017.

53. Yang, W.; Li, X.; Liu, G.; Zhang, B.; Zhang, Y.; Kong, T.; Tang, J.; Li, D.; Wang, Z. A Colloidal Gold Probe-Based Silver Enhancement Immunochromatographic Assay for the Rapid Detection of Abrin-A. *Biosens. Bioelectron.* **2011**, *26*, 3710–3713, doi:10.1016/j.bios.2011.02.016.
54. Drygin, Y.F.; Blintsov, A.N.; Grigorenko, V.G.; Andreeva, I.P.; Osipov, A.P.; Varitzev, Y. a.; Uskov, A.I.; Kravchenko, D. V.; Atabekov, J.G. Highly Sensitive Field Test Lateral Flow Immunodiagnostics of PVX Infection. *Appl. Microbiol. Biotechnol.* **2012**, *93*, 179–189, doi:10.1007/s00253-011-3522-x.
55. Liu, C.C.; Yeung, C.Y.; Chen, P.H.; Yeh, M.K.; Hou, S.Y. Salmonella Detection Using 16S Ribosomal DNA/RNA Probe-Gold Nanoparticles and Lateral Flow Immunoassay. *Food Chem.* **2013**, *141*, 2526–2532, doi:10.1016/j.foodchem.2013.05.089.
56. Anfossi, L.; Di Nardo, F.; Giovannoli, C.; Passini, C.; Baggiani, C. Increased Sensitivity of Lateral Flow Immunoassay for Ochratoxin A through Silver Enhancement. *Anal. Bioanal. Chem.* **2013**, *405*, 9859–9867, doi:10.1007/s00216-013-7428-6.
57. Xing, C.; Kuang, H.; Hao, C.; Liu, L.; Wang, L.; Xu, C. A Silver Enhanced and Sensitive Strip Sensor for Cadmium Detection. *Food Agric. Immunol.* **2014**, *25*, 287–300, doi:10.1080/09540105.2013.781140.
58. Chiao, D.J.; Shyu, R.H.; Hu, C.S.; Chiang, H.Y.; Tang, S.S. Colloidal Gold-Based Immunochromatographic Assay for Detection of Botulinum Neurotoxin Type B. *J. Chromatogr. B Anal. Technol. Biomed. Life Sci.* **2004**, *809*, 37–41, doi:10.1016/j.jchromb.2004.05.033.
59. Chiao, D.-J.; Wey, J.-J.; Shyu, R.-H.; Tang, S.-S. Monoclonal Antibody-Based Lateral Flow Assay for Detection of Botulinum Neurotoxin Type A. *Hybridoma* **2008**, *27*, 31–35, doi:10.1089/hyb.2007.0550.
60. Yu, Q.; Li, H.; Li, C.; Zhang, S.; Shen, J.; Wang, Z. Gold Nanoparticles-Based Lateral Flow Immunoassay with Silver Staining for Simultaneous Detection of Fumonisin B1 and Deoxynivalenol. *Food Control* **2015**, *54*, 347–352, doi:10.1016/j.foodcont.2015.02.019.
61. Byzova, N.A.; Zherdev, A. V.; Sveshnikov, P.G.; Sadykhov, E.G.; Dzantiev, B.B. Development of an Immunochromatographic Test System for the Detection of Helicobacter Pylori Antigens. *Appl. Biochem. Microbiol.* **2015**, *51*, 608–617, doi:10.1134/S000368381505004X.
62. Rodríguez, M.O.; Covián, L.B.; García, A.C.; Blanco-López, M.C. Silver and Gold Enhancement Methods for Lateral Flow Immunoassays. *Talanta* **2016**, *148*, 272–278, doi:10.1016/j.talanta.2015.10.068.
63. Panferov, V.G.; Safenkova, I. V.; Varitsev, Y.A.; Drenova, N. V.; Kornev, K.P.; Zherdev, A. V.; Dzantiev, B.B. Development of the Sensitive Lateral Flow Immunoassay with Silver Enhancement for the Detection of Ralstonia Solanacearum in Potato Tubers. *Talanta* **2016**, *152*, 521–530, doi:10.1016/j.talanta.2016.02.050.
64. Apilux, A.; Rengpipat, S.; Suwanjang, W.; Chailapakul, O. Development of Competitive Lateral Flow Immunoassay Coupled with Silver Enhancement for Simple and Sensitive Salivary Cortisol Detection. *EXCLI J.* **2018**, *17*, 1198–1209, doi:10.17179/excli2018-1824.
65. Kim, W.; Lee, S.; Jeon, S. Enhanced Sensitivity of Lateral Flow Immunoassays by Using Water-Soluble Nanofibers and Silver-Enhancement Reactions. *Sensors Actuators, B Chem.* **2018**, *273*, 1323–1327, doi:10.1016/j.snb.2018.07.045.
66. Panferov, V.G.; Safenkova, I. V.; Byzova, N.A.; Varitsev, Y.A.; Zherdev, A. V.; Dzantiev, B.B. Silver-Enhanced Lateral Flow Immunoassay for Highly-Sensitive Detection of Potato Leafroll Virus. *Food Agric. Immunol.* **2018**, *29*, 445–457, doi:10.1080/09540105.2017.1401044.
67. Gholamzad, M.; Seyedkhan, Z.; Morovati Khamsi, H.; Khorshidi, M. High Sensitive Detection of Staphylococcal Enterotoxin B by Silver Enhanced Lateral Flow Assay. *Iran. J. Med. Microbiol.* **2020**, *14*, 307–313, doi:10.30699/ijmm.14.4.307.
68. Rahbar, M.; Wu, Y.; Subramony, J.A.; Liu, G. Sensitive Colorimetric Detection of Interleukin-6 via Lateral Flow Assay Incorporated Silver Amplification Method. *Front. Bioeng. Biotechnol.* **2021**, *9*, 1–8, doi:10.3389/fbioe.2021.778269.
69. Poosinuntakul, N.; Chanmee, T.; Porntadavity, S.; Chailapakul, O.; Apilux, A. Silver-Enhanced Colloidal Gold Dip Strip Immunoassay Integrated with Smartphone-Based Colorimetry for Sensitive Detection of Cardiac Marker Troponin I. *Sci. Rep.* **2022**, *12*, 19866, doi:10.1038/s41598-022-24458-1.
70. Zvereva, E.A.; Hendrickson, O.D.; Zherdev, A. V.; Dzantiev, B.B. Silver-Enhanced Lateral Flow Immunoassay for Detection of Microcystin-LR in Drinking Water. *Int. J. Environ. Anal. Chem.* **2022**, *00*, 1–10, doi:10.1080/03067319.2022.2086052.
71. Rong-Hwa, S.; Shiao-Shek, T.; Der-Jiang, C.; Yao-Wen, H. Gold Nanoparticle-Based Lateral Flow Assay for Detection of Staphylococcal Enterotoxin B. *Food Chem.* **2010**, *118*, 462–466, doi:10.1016/j.foodchem.2009.04.106.
72. Shyu, R.H.; Shyu, H.F.; Liu, H.W.; Tang, S.S. Colloidal Gold-Based Immunochromatographic Assay for Detection of Ricin. *Toxicon* **2002**, *40*, 255–258, doi:10.1016/S0041-0101(01)00193-3.
73. Yao, L.; Teng, J.; Qu, H.; Zhu, M.; Zheng, L.; Xue, F.; Chen, W. Paper Matrix Based Array for Rapid and Sensitive Optical Detection of Mercury Ions Using Silver Enhancement. *Microchim. Acta* **2017**, *184*, 569–576, doi:10.1007/s00604-016-2052-8.
74. Hou, S.Y.; Hsiao, Y.L.; Lin, M.S.; Yen, C.C.; Chang, C.S. MicroRNA Detection Using Lateral Flow Nucleic Acid Strips with Gold Nanoparticles. *Talanta* **2012**, *99*, 375–379, doi:10.1016/j.talanta.2012.05.067.
75. Wang, Y.; Liu, P.; Ye, Y.; Hammock, B.D.; Zhang, C. An Integrated Approach to Improve the Assay Performance of Quantum Dot-Based Lateral Flow Immunoassays by Using Silver Deposition. *Microchem. J.* **2023**, *192*, 108932, doi:10.1016/j.microc.2023.108932.
76. Bazsefidpar, S.; Serrano-Pertierra, E.; Gutiérrez, G.; Calvo, A.S.; Matos, M.; Blanco-López, M.C. Rapid and Sensitive Detection of E. Coli O157:H7 by Lateral Flow Immunoassay and Silver Enhancement. *Microchim. Acta* **2023**, *190*, 264, doi:10.1007/s00604-023-05834-8.
77. Fu, J.; Zhou, Y.; Huang, X.; Zhang, W.; Wu, Y.; Fang, H.; Zhang, C.; Xiong, Y. Dramatically Enhanced Immunochromatographic Assay Using Cascade Signal Amplification for Ultrasensitive Detection of Escherichia Coli O157:H7 in Milk. *J. Agric. Food Chem.* **2020**, *68*, 1118–1125, doi:10.1021/acs.jafc.9b07076.

78. Kumar, R.; Singh, C.K.; Kamle, S.; Sinha, R.P.; Bhatnagar, R.K.; Kachru, D.N. Development of Nanocolloidal Gold Based Immunochromatographic Assay for Rapid Detection of Transgenic Vegetative Insecticidal Protein in Genetically Modified Crops. *Food Chem.* **2010**, *122*, 1298–1303, doi:10.1016/j.foodchem.2010.03.086.
79. Fu, E.; Liang, T.; Houghtaling, J.; Ramachandran, S.; Ramsey, S. a; Lutz, B.; Yager, P. Enhanced Sensitivity of Lateral Flow Tests Using a Two-Dimensional Paper Network Format. *Anal. Chem.* **2011**, *83*, 7941–7946, doi:10.1021/ac201950g.
80. Fu, E.; Liang, T.; Spicar-Mihalic, P.; Houghtaling, J.; Ramachandran, S.; Yager, P. Two-Dimensional Paper Network Format That Enables Simple Multistep Assays for Use in Low-Resource Settings in the Context of Malaria Antigen Detection. *Anal. Chem.* **2012**, *84*, 4574–4579, doi:10.1021/ac300689s.
81. Li, J.; Zou, M.; Chen, Y.; Xue, Q.; Zhang, F.; Li, B.; Wang, Y.; Qi, X.; Yang, Y. Gold Immunochromatographic Strips for Enhanced Detection of Avian Influenza and Newcastle Disease Viruses. *Anal. Chim. Acta* **2013**, *782*, 54–58, doi:10.1016/j.aca.2013.04.022.
82. Wang, J.Y.; Chen, M.H.; Sheng, Z.C.; Liu, D.F.; Wu, S.S.; Lai, W.H. Development of Colloidal Gold Immunochromatographic Signal-Amplifying System for Ultrasensitive Detection of Escherichia Coli O157:H7 in Milk. *RSC Adv.* **2015**, *5*, 62300–62305, doi:10.1039/c5ra13279g.
83. Park, J.; Shin, J.H.; Park, J.K. Pressed Paper-Based Dipstick for Detection of Foodborne Pathogens with Multistep Reactions. *Anal. Chem.* **2016**, *88*, 3781–3788, doi:10.1021/acs.analchem.5b04743.
84. Han, K.N.; Choi, J.-S.; Kwon, J. Three-Dimensional Paper-Based Slip Device for One-Step Point-of-Care Testing. *Sci. Rep.* **2016**, *6*, 25710, doi:10.1038/srep25710.
85. Dias, J.T.; Svedberg, G.; Nystrand, M.; Andersson-Svahn, H.; Gantelius, J. Rapid Signal Enhancement Method for Nanoprobe-Based Biosensing. *Sci. Rep.* **2017**, *7*, 6837, doi:10.1038/s41598-017-07030-0.
86. Bu, T.; Huang, Q.; Yan, L.; Huang, L.; Zhang, M.; Yang, Q.; Yang, B.; Wang, J.; Zhang, D. Ultra Technically-Simple and Sensitive Detection for Salmonella Enteritidis by Immunochromatographic Assay Based on Gold Growth. *Food Control* **2018**, *84*, 536–543, doi:10.1016/j.foodcont.2017.08.036.
87. Panferov, V.G.; Safenkova, I. V.; Zherdev, A. V.; Dzantiev, B.B. Post-Assay Growth of Gold Nanoparticles as a Tool for Highly Sensitive Lateral Flow Immunoassay. Application to the Detection of Potato Virus X. *Microchim. Acta* **2018**, *185*, doi:10.1007/s00604-018-3052-7.
88. Razo, S.C.; Panferova, N.A.; Panferov, V.G.; Safenkova, I. V.; Drenova, N. V.; Varitsev, Y.A.; Zherdev, A. V.; Pakina, E.N.; Dzantiev, B.B. Enlargement of Gold Nanoparticles for Sensitive Immunochromatographic Diagnostics of Potato Brown Rot. *Sensors* **2019**, *19*, 153, doi:10.3390/s19010153.
89. Han, G.R.; Koo, H.J.; Ki, H.; Kim, M.G. Paper/Soluble Polymer Hybrid-Based Lateral Flow Biosensing Platform for High-Performance Point-of-Care Testing. *ACS Appl. Mater. Interfaces* **2020**, *12*, 34564–34575, doi:10.1021/acsami.0c07893.
90. Zhao, M.; Yao, X.; Liu, S.; Zhang, H.; Wang, L.; Yin, X.; Su, L.; Xu, B.; Wang, J.; Lan, Q.; et al. Antibiotic and Mammal IgG Based Lateral Flow Assay for Simple and Sensitive Detection of Staphylococcus Aureus. *Food Chem.* **2021**, *339*, 127955, doi:10.1016/j.foodchem.2020.127955.
91. Panraksa, Y.; Apilux, A.; Jampasa, S.; Puthong, S.; Henry, C.S.; Rengpipat, S.; Chailapakul, O. A Facile One-Step Gold Nanoparticles Enhancement Based on Sequential Patterned Lateral Flow Immunoassay Device for C-Reactive Protein Detection. *Sensors Actuators, B Chem.* **2021**, *329*, 129241, doi:10.1016/j.snb.2020.129241.
92. Thangavelu, R.M.; Kadirvel, N.; Balasubramaniam, P.; Viswanathan, R. Ultrasensitive Nano-Gold Labelled, Duplex Lateral Flow Immunochromatographic Assay for Early Detection of Sugarcane Mosaic Viruses. *Sci. Rep.* **2022**, *12*, 4144, doi:10.1038/s41598-022-07950-6.
93. Preechakasedkit, P.; Teekayupak, K.; Citterio, D.; Ruecha, N. Improvement in Sensitivity for Lateral Flow Immunoassay of Ferritin Using Novel Device Design Based on Gold-Enhanced Gold Nanoparticles. *Sci. Rep.* **2022**, *12*, 7831, doi:10.1038/s41598-022-11732-5.
94. Duan, H.; Ma, T.; Huang, X.; Gao, B.; Zheng, L.; Chen, X.; Xiong, Y.; Chen, X. Avoiding the Self-Nucleation Interference: A PH-Regulated Gold in Situ Growth Strategy to Enable Ultrasensitive Immunochromatographic Diagnostics. *Theranostics* **2022**, *12*, 2801–2810, doi:10.7150/thno.70092.
95. Taranova, N.A.; Bulanaya, A.A.; Zherdev, A. V.; Dzantiev, B.B. Triple Enhancement for Sensitive Immunochromatographic Assay: A Case Study for Human Fatty Acid-Binding Protein Detection. *Biosensors* **2022**, *12*, doi:10.3390/bios12121166.
96. Ruantip, S.; Pimpitak, U.; Rengpipat, S.; Pasomsub, E.; Seepiban, C.; Gajanandana, O.; Torvorapanit, P.; Hirankarn, N.; Jaruampornpan, P.; Siwamogsatham, S.; et al. Self-Enhancement Lateral Flow Immunoassay for COVID-19 Diagnosis. *Sensors Actuators B Chem.* **2023**, *389*, 133898, doi:10.1016/j.snb.2023.133898.
97. Presnova, G. V.; Zhdanov, G.A.; Filatova, L.Y.; Ulyashova, M.M.; Presnov, D.E.; Rubtsova, M.Y. Improvement of Seed-Mediated Growth of Gold Nanoparticle Labels for DNA Membrane-Based Assays. *Biosensors* **2022**, *13*, 2, doi:10.3390/bios13010002.
98. Kaur, J.; Singh, K.V.; Boro, R.; Thampi, K.R.; Raje, M.; Varshney, G.C.; Suri, C.R. Immunochromatographic Dipstick Assay Format Using Gold Nanoparticles Labeled Protein-Hapten Conjugate for the Detection of Atrazine. *Environ. Sci. Technol.* **2007**, *41*, 5028–5036, doi:10.1021/es070194j.
99. Yang, J.S.; Shin, J.; Choi, S.; Jung, H. Il Smartphone Diagnostics Unit (SDU) for the Assessment of Human Stress and Inflammation Level Assisted by Biomarker Ink, Fountain Pen, and Origami Holder for Strip Biosensor. *Sensors Actuators, B Chem.* **2017**, *241*, 80–84, doi:10.1016/j.snb.2016.10.052.
100. Sharma, A.; Tok, A.I.Y.; Alagappan, P.; Liedberg, B. Gold Nanoparticle Conjugated Magnetic Beads for Extraction and Nucleation Based Signal Amplification in Lateral Flow Assaying. *Sensors Actuators, B Chem.* **2020**, *312*, 127959, doi:10.1016/j.snb.2020.127959.

101. Han, D.K.; Oh, J.; Lee, J.; Cho, Y.G.; Park, J.S.; Choi, J.S.; Kim, D.S.; Kwon, J. Paper-Based Multiplex Analytical Device for Simultaneous Detection of Clostridioides Difficile Toxins and Glutamate Dehydrogenase. *Biosens. Bioelectron.* **2021**, *176*, 112894, doi:10.1016/j.bios.2020.112894.
102. Kim, H.-S.; Ko, H.; Kang, M.-J.; Pyun, J.-C. Highly Sensitive Rapid Test with Chemiluminescent Signal Bands. *BioChip J.* **2010**, *4*, 155–160, doi:10.1007/s13206-010-4211-z.
103. Renzi, E.; Piper, A.; Nastri, F.; Merkoçi, A.; Lombardi, A. An Artificial Miniaturized Peroxidase for Signal Amplification in Lateral Flow Immunoassays. *Small* **2023**, *2207949*, doi:10.1002/smll.202207949.
104. He, Y.; Zhang, S.; Zhang, X.; Baloda, M.; Gurung, A.S.; Xu, H.; Zhang, X.; Liu, G. Ultrasensitive Nucleic Acid Biosensor Based on Enzyme-Gold Nanoparticle Dual Label and Lateral Flow Strip Biosensor. *Biosens. Bioelectron.* **2011**, *26*, 2018–2024, doi:10.1016/j.bios.2010.08.079.
105. Cho, I.H.; Bhunia, A.; Irudayaraj, J. Rapid Pathogen Detection by Lateral-Flow Immunochromatographic Assay with Gold Nanoparticle-Assisted Enzyme Signal Amplification. *Int. J. Food Microbiol.* **2015**, *206*, 60–66, doi:10.1016/j.ijfoodmicro.2015.04.032.
106. Kim, K.; Joung, H.A.; Han, G.R.; Kim, M.G. An Immunochromatographic Biosensor Combined with a Water-Swellable Polymer for Automatic Signal Generation or Amplification. *Biosens. Bioelectron.* **2016**, *85*, 422–428, doi:10.1016/j.bios.2016.04.096.
107. Cho, J.-H.; Paek, E.-H.; Cho, I.-H.; Paek, S.-H. An Enzyme Immunoanalytical System Based on Sequential Cross-Flow Chromatography. *Anal. Chem.* **2005**, *77*, 4091–4097, doi:10.1021/ac048270d.
108. Byers, K.M.; Bird, A.R.; Cho, H.D.D.; Linnes, J.C. Fully Dried Two-Dimensional Paper Network for Enzymatically Enhanced Detection of Nucleic Acid Amplicons. *ACS Omega* **2020**, *5*, 4673–4681, doi:10.1021/acsomega.0c00115.
109. Tominaga, T. Enhanced Sensitivity of Lateral-Flow Test Strip Immunoassays Using Colloidal Palladium Nanoparticles and Horseradish Peroxidase. *Lwt* **2017**, *86*, 566–570, doi:10.1016/j.lwt.2017.08.027.
110. Parolo, C.; de la Escosura-Muñiz, A.; Merkoçi, A. Enhanced Lateral Flow Immunoassay Using Gold Nanoparticles Loaded with Enzymes. *Biosens. Bioelectron.* **2013**, *40*, 412–416, doi:10.1016/j.bios.2012.06.049.
111. Han, G.-R.; Ki, H.; Kim, M.-G. Automated, Universal, and Mass-Producible Paper-Based Lateral Flow Biosensing Platform for High-Performance Point-of-Care Testing. *ACS Appl. Mater. Interfaces* **2020**, *12*, 1885–1894, doi:10.1021/acsami.9b17888.
112. Cho, I.H.; Paek, E.H.; Kim, Y.K.; Kim, J.H.; Paek, S.H. Chemiluminometric Enzyme-Linked Immunosorbent Assays (ELISA)-on-a-Chip Biosensor Based on Cross-Flow Chromatography. *Anal. Chim. Acta* **2009**, *632*, 247–255, doi:10.1016/j.aca.2008.11.019.
113. Deng, J.; Yang, M.; Wu, J.; Zhang, W.; Jiang, X. A Self-Contained Chemiluminescent Lateral Flow Assay for Point-of-Care Testing. *Anal. Chem.* **2018**, *90*, 9132–9137, doi:10.1021/acs.analchem.8b01543.
114. Mao, X.; Ma, Y.; Zhang, A.; Zhang, L.; Zeng, L.; Liu, G. Disposable Nucleic Acid Biosensors Based on Gold Nanoparticle Probes and Lateral Flow Strip. *Anal. Chem.* **2009**, *81*, 1660–1668, doi:10.1021/ac8024653.
115. Hendrickson, O.D.; Zvereva, E.A.; Zherdev, A. V.; Dzantiev, B.B. Ultrasensitive Lateral Flow Immunoassay of Phycotoxin Microcystin-LR in Seafood Based on Magnetic Particles and Peroxidase Signal Amplification. *Food Control* **2022**, *133*, 108655, doi:10.1016/j.foodcont.2021.108655.
116. Ono, T.; Kawamura, M.; Arao, S.; Nariuchi, H. A Highly Sensitive Quantitative Immunochromatography Assay for Antigen-Specific IgE. *J. Immunol. Methods* **2003**, *272*, 211–218, doi:10.1016/S0022-1759(02)00504-5.
117. Zhang, J.; Gui, X.; Zheng, Q.; Chen, Y.; Ge, S.; Zhang, J.; Xia, N. An HRP-Labeled Lateral Flow Immunoassay for Rapid Simultaneous Detection and Differentiation of Influenza A and B Viruses. *J. Med. Virol.* **2019**, *91*, 503–507, doi:10.1002/jmv.25322.
118. Panferov, V.G.; Safenkova, I. V.; Varitsev, Y.A.; Zherdev, A. V.; Dzantiev, B.B. Enhancement of Lateral Flow Immunoassay by Alkaline Phosphatase: A Simple and Highly Sensitive Test for Potato Virus X. *Microchim. Acta* **2018**, *185*, 25, doi:10.1007/s00604-017-2595-3.
119. Chen, Y.; Sun, J.; Xianyu, Y.; Yin, B.; Niu, Y.; Wang, S.; Cao, F.; Zhang, X.; Wang, Y.; Jiang, X. A Dual-Readout Chemiluminescent-Gold Lateral Flow Test for Multiplex and Ultrasensitive Detection of Disease Biomarkers in Real Samples. *Nanoscale* **2016**, *8*, 15205–15212, doi:10.1039/C6NR04017A.
120. Han, G.-R.; Kim, M.-G. Highly Sensitive Chemiluminescence-Based Lateral Flow Immunoassay for Cardiac Troponin I Detection in Human Serum. *Sensors* **2020**, *20*, 2593, doi:10.3390/s20092593.
121. Gao, X.; Xu, L.P.; Wu, T.; Wen, Y.; Ma, X.; Zhang, X. An Enzyme-Amplified Lateral Flow Strip Biosensor for Visual Detection of MicroRNA-224. *Talanta* **2016**, *146*, 648–654, doi:10.1016/j.talanta.2015.06.060.
122. Zhang, C.; Zhang, Y.; Wang, S. Development of Multianalyte Flow-through and Lateral-Flow Assays Using Gold Particles and Horseradish Peroxidase as Tracers for the Rapid Determination of Carbaryl and Endosulfan in Agricultural Products. *J. Agric. Food Chem.* **2006**, *54*, 2502–2507, doi:10.1021/jf0531407.
123. Zhu, X.; Sarwar, M.; Zhu, J.J.; Zhang, C.; Kaushik, A.; Li, C.Z. Using a Glucose Meter to Quantitatively Detect Disease Biomarkers through a Universal Nanozyme Integrated Lateral Fluidic Sensing Platform. *Biosens. Bioelectron.* **2019**, *126*, 690–696, doi:10.1016/j.bios.2018.11.033.
124. Li, J.; Baird, M.A.; Davis, M.A.; Tai, W.; Zweifel, L.S.; Waldorf, K.M.A.; Gale, M.; Rajagopal, L.; Pierce, R.H.; Gao, X. Dramatic Enhancement of the Detection Limits of Bioassays via Ultrafast Deposition of Polydopamine. *Nat. Biomed. Eng.* **2017**, *1*, doi:10.1038/s41551-017-0082.
125. Tran, T. V.; Nguyen, B. V.; Nguyen, T.T.P.; Tran, T.T.; Pham, K.G.; Le, Q.B.; Do, B.N.; Pham, H.N.; Nguyen, C. V.; Dinh, D.P.H.; et al. Development of a Highly Sensitive Magneto-Enzyme Lateral Flow Immunoassay for Dengue NS1 Detection. *PeerJ* **2019**, *7*, e7779, doi:10.7717/peerj.7779.
126. Hendrickson, O.D.; Zvereva, E.A.; Zherdev, A. V.; Dzantiev, B.B. Cascade-Enhanced Lateral Flow Immunoassay for Sensitive Detection of Okadaic Acid in Seawater, Fish, and Seafood. *Foods* **2022**, *11*, 1691, doi:10.3390/foods11121691.

127. Huang, X.; Zhou, Y.; Ding, L.; Yu, G.; Leng, Y.; Lai, W.; Xiong, Y.; Chen, X. Supramolecular Recognition-Mediated Layer-by-Layer Self-Assembled Gold Nanoparticles for Customized Sensitivity in Paper-Based Strip Nanobiosensors. *Small* **2019**, *15*, 1903861, doi:10.1002/smll.201903861.
128. Shu, R.; Liu, S.; Xu, J.; Wang, S.; Ma, Y.; Chen, Y.; Li, Y.; Sun, J.; Zhang, D.; Wang, J. Galvanic Replacement Inspired Signal Amplification: Background-Free and Antibody-Thrift in-Situ Growth Immunochromatography. *Chem. Eng. J.* **2022**, *437*, 135362, doi:10.1016/j.cej.2022.135362.
129. Zhang, C.; Hu, J.; Wu, X.; Shi, J.; Hammock, B.D. Development of the Au@Pt-Labeled Nanobody Lateral-Flow Nanozyme Immunoassay for Visual Detection of 3-Phenoxybenzoic Acid in Milk and Lake Water. *ACS Agric. Sci. Technol.* **2022**, *2*, 573–579, doi:10.1021/acsagscitech.2c00018.
130. Cheng, N.; Song, Y.; Zeinhom, M.M.A.; Chang, Y.C.; Sheng, L.; Li, H.; Du, D.; Li, L.; Zhu, M.J.; Luo, Y.; et al. Nanozyme-Mediated Dual Immunoassay Integrated with Smartphone for Use in Simultaneous Detection of Pathogens. *ACS Appl. Mater. Interfaces* **2017**, *9*, 40671–40680, doi:10.1021/acsami.7b12734.
131. Loynachan, C.N.; Thomas, M.R.; Gray, E.R.; Richards, D.A.; Kim, J.; Miller, B.S.; Brookes, J.C.; Agarwal, S.; Chudasama, V.; McKendry, R.A.; et al. Platinum Nanocatalyst Amplification: Redefining the Gold Standard for Lateral Flow Immunoassays with Ultrabroad Dynamic Range. *ACS Nano* **2018**, *12*, 279–288, doi:10.1021/acsnano.7b06229.
132. Wei, Z.; Luciano, K.; Xia, X. Catalytic Gold-Iridium Nanoparticles as Labels for Sensitive Colorimetric Lateral Flow Assay. *ACS Nano* **2022**, *16*, 21609–21617, doi:10.1021/acsnano.2c10574.
133. Chen, R.; Chen, X.; Zhou, Y.; Lin, T.; Leng, Y.; Huang, X.; Xiong, Y. "Three-in-One" Multifunctional Nanohybrids with Colorimetric Magnetic Catalytic Activities to Enhance Immunochromatographic Diagnosis. *ACS Nano* **2022**, *16*, 3351–3361, doi:10.1021/acsnano.2c00008.
134. Jiang, T.; Song, Y.; Du, D.; Liu, X.; Lin, Y. Detection of P53 Protein Based on Mesoporous Pt-Pd Nanoparticles with Enhanced Peroxidase-like Catalysis. *ACS Sensors* **2016**, *1*, 717–724, doi:10.1021/acssensors.6b00019.
135. Yang, H.; He, Q.; Pan, J.; Shen, D.; Xiao, H.; Cui, X.; Zhao, S. A Pt-Ir Nanocube Amplified Lateral Flow Immunoassay for Dehydroepiandrosterone. *Analyst* **2021**, *146*, 2726–2733, doi:10.1039/d0an02293d.
136. Zheng, C.; Jiang, Q.; Wang, K.; Li, T.; Zheng, W.; Cheng, Y.; Ning, Q.; Cui, D. Nanozyme Enhanced Magnetic Immunoassay for Dual-Mode Detection of Gastrin-17. *Analyst* **2022**, *147*, 1678–1687, doi:10.1039/d2an00063f.
137. Jin, X.; Chen, L.; Zhang, Y.; Wang, X.; Zhou, N. A Lateral Flow Strip for On-Site Detection of Tobramycin Based on Dual-Functional Platinum-Decorated Gold Nanoparticles. *Analyst* **2021**, *146*, 3608–3616, doi:10.1039/d1an00403d.
138. Bradbury, D.W.; Trinh, J.T.; Ryan, M.J.; Cantu, C.M.; Lu, J.; Nicklen, F.D.; Du, Y.; Sun, R.; Wu, B.M.; Kamei, D.T. On-Demand Nanozyme Signal Enhancement at the Push of a Button for the Improved Detection of SARS-CoV-2 Nucleocapsid Protein in Serum. *Analyst* **2021**, *146*, 7483–7490, doi:10.1039/d1an01350e.
139. Wei, D.; Zhang, X.; Chen, B.; Zeng, K. Using Bimetallic Au@Pt Nanozymes as a Visual Tag and as an Enzyme Mimic in Enhanced Sensitive Lateral-Flow Immunoassays: Application for the Detection of Streptomycin. *Anal. Chim. Acta* **2020**, *1126*, 106–113, doi:10.1016/j.aca.2020.06.009.
140. Nicklen, F.D.; Diaz, A.J.; Lu, J.; Patel, S.T.; Zheng, E.M.; Campbell, V.R.; Wu, B.M.; Kamei, D.T. Application of the Aqueous Two-Phase System and Nanozyme Signal Enhancement for the Improved Detection of Plasmodium Lactate Dehydrogenase in Serum. *Anal. Bioanal. Chem.* **2022**, *414*, 7949–7956, doi:10.1007/s00216-022-04346-3.
141. Ouyang, H.; Lu, Q.; Wang, W.; Song, Y.; Tu, X.; Zhu, C.; Smith, J.N.; Du, D.; Fu, Z.; Lin, Y. Dual-Readout Immunochromatographic Assay by Utilizing MnO2 Nanoflowers as the Unique Colorimetric/Chemiluminescent Probe. *Anal. Chem.* **2018**, *90*, 5147–5152, doi:10.1021/acs.analchem.7b05247.
142. Bradbury, D.W.; Azimi, M.; Diaz, A.J.; Pan, A.A.; Falktoft, C.H.; Wu, B.M.; Kamei, D.T. Automation of Biomarker Preconcentration, Capture, and Nanozyme Signal Enhancement on Paper-Based Devices. *Anal. Chem.* **2019**, *91*, 12046–12054, doi:10.1021/acs.analchem.9b03105.
143. Sun, Y.; Xie, Z.; Pei, F.; Hu, W.; Feng, S.; Hao, Q.; Liu, B.; Mu, X.; Lei, W.; Tong, Z. Trimetallic Au@Pd@Pt Nanozyme-Enhanced Lateral Flow Immunoassay for the Detection of SARS-CoV-2 Nucleocapsid Protein. *Anal. Methods* **2022**, *14*, 5091–5099, doi:10.1039/d2ay01530g.
144. Yang, D.; Wang, L.; Jia, T.; Lian, T.; Yang, K.; Li, X.; Wang, X.; Xue, C. Au/Fe3O4-Based Nanozymes with Peroxidase-like Activity Integrated in Immunochromatographic Strips for Highly-Sensitive Biomarker Detection. *Anal. Methods* **2023**, *15*, 663–674, doi:10.1039/d2ay01815b.
145. Wang, B.; Moyano, A.; Duque, J.M.; Sánchez, L.; García-Santos, G.; Flórez, L.J.G.; Serrano-Pertierra, E.; Blanco-López, M.D.C. Nanozyme-Based Lateral Flow Immunoassay (LFIA) for Extracellular Vesicle Detection. *Biosensors* **2022**, *12*, 1–13, doi:10.3390/bios12070490.
146. Hendrickson, O.D.; Zvereva, E.A.; Panferov, V.G.; Solopova, O.N.; Zherdev, A. V; Sveshnikov, P.G.; Dzantiev, B.B. Application of Au@Pt Nanozyme as Enhancing Label for the Sensitive Lateral Flow Immunoassay of Okadaic Acid. *Biosensors* **2022**, *12*, 1137, doi:10.3390/bios12121137.
147. Chen, Y.; Ma, J.; Yin, X.; Deng, Z.; Liu, X.; Yang, D.; Zhao, L.; Sun, J.; Wang, J.; Zhang, D. Joint-Detection of Salmonella Typhimurium and Escherichia Coli O157:H7 by an Immersible Amplification Dip-Stick Immunoassay. *Biosens. Bioelectron.* **2023**, *224*, 115075, doi:10.1016/j.bios.2023.115075.
148. Bai, T.; Wang, L.; Wang, M.; Zhu, Y.; Li, W.; Guo, Z.; Zhang, Y. Strategic Synthesis of Trimetallic Au@Ag–Pt Nanorattles for Ultrasensitive Colorimetric Detection in Lateral Flow Immunoassay. *Biosens. Bioelectron.* **2022**, *208*, 114218, doi:10.1016/j.bios.2022.114218.

149. Liang, M.; Cai, X.; Gao, Y.; Yan, H.; Fu, J.; Tang, X.; Zhang, Q.; Li, P. A Versatile Nanozyme Integrated Colorimetric and Photothermal Lateral Flow Immunoassay for Highly Sensitive and Reliable Aspergillus Flavus Detection. *Biosens. Bioelectron.* **2022**, *213*, 114435, doi:10.1016/j.bios.2022.114435.
150. Liu, S.; Dou, L.; Yao, X.; Zhang, W.; Zhao, M.; Yin, X.; Sun, J.; Zhang, D.; Wang, J. Nanozyme Amplification Mediated On-Demand Multiplex Lateral Flow Immunoassay with Dual-Readout and Broadened Detection Range. *Biosens. Bioelectron.* **2020**, *169*, 112610, doi:10.1016/j.bios.2020.112610.
151. Cheng, N.; Shi, Q.; Zhu, C.; Li, S.; Lin, Y.; Du, D. Pt–Ni(OH)2 Nanosheets Amplified Two-Way Lateral Flow Immunoassays with Smartphone Readout for Quantification of Pesticides. *Biosens. Bioelectron.* **2019**, *142*, 1–7, doi:10.1016/j.bios.2019.111498.
152. Dou, L.; Bai, Y.; Liu, M.; Shao, S.; Yang, H.; Yu, X.; Wen, K.; Wang, Z.; Shen, J.; Yu, W. 'Three-To-One' Multi-Functional Nanocomposite-Based Lateral Flow Immunoassay for Label-Free and Dual-Readout Detection of Pathogenic Bacteria. *Biosens. Bioelectron.* **2022**, *204*, 114093, doi:10.1016/j.bios.2022.114093.
153. Liu, D.; Ju, C.; Han, C.; Shi, R.; Chen, X.; Duan, D.; Yan, J.; Yan, X. Nanozyme Chemiluminescence Paper Test for Rapid and Sensitive Detection of SARS-CoV-2 Antigen. *Biosens. Bioelectron.* **2021**, *173*, 112817, doi:10.1016/j.bios.2020.112817.
154. Duan, D.; Fan, K.; Zhang, D.; Tan, S.; Liang, M.; Liu, Y.; Zhang, J.; Zhang, P.; Liu, W.; Qiu, X.; et al. Nanozyme-Strip for Rapid Local Diagnosis of Ebola. *Biosens. Bioelectron.* **2015**, *74*, 134–141, doi:10.1016/j.bios.2015.05.025.
155. Liu, S.; Shu, R.; Ma, J.; Dou, L.; Zhang, W.; Wang, S.; Ji, Y.; Li, Y.; Xu, J.; Zhang, D.; et al. Mussel-Inspired Fe-Based Tannic Acid Nanozyme: A Renewable Bioresource-Derived High-Affinity Signal Tag for Dual-Readout Multiplex Lateral Flow Immunoassay. *Chem. Eng. J.* **2022**, *446*, 137382, doi:10.1016/j.cej.2022.137382.
156. Ren, J.; Su, L.; Hu, H.; Yin, X.; Xu, J.; Liu, S.; Wang, J.; Wang, Z.; Zhang, D. Expanded Detection Range of Lateral Flow Immunoassay Endowed with a Third-Stage Amplifier Indirect Probe. *Food Chem.* **2022**, *377*, 131920, doi:10.1016/j.foodchem.2021.131920.
157. Cai, X.; Liang, M.; Ma, F.; Zhang, Z.; Tang, X.; Jiang, J.; Guo, C.; Ramzy Mohamed, S.; Abdel Goda, A.; Dawood, D.H.; et al. Nanozyme-Strip Based on MnO2 Nanosheets as a Catalytic Label for Multi-Scale Detection of Aflatoxin B1 with an Ultrabroad Working Range. *Food Chem.* **2022**, *377*, doi:10.1016/j.foodchem.2021.131965.
158. Bu, T.; Bai, F.; Sun, X.; Tian, Y.; Zhang, M.; Zhao, S.; He, K.; Wang, X.; Jia, P.; Wang, L. An Innovative Prussian Blue Nanocubes Decomposition-Assisted Signal Amplification Strategy Suitable for Competitive Lateral Flow Immunoassay to Sensitively Detect Aflatoxin B1. *Food Chem.* **2021**, *344*, 128711, doi:10.1016/j.foodchem.2020.128711.
159. Han, J.; Zhang, L.; Hu, L.; Xing, K.; Lu, X.; Huang, Y.; Zhang, J.; Lai, W.; Chen, T. Nanozyme-Based Lateral Flow Assay for the Sensitive Detection of Escherichia Coli O157:H7 in Milk. *J. Dairy Sci.* **2018**, *101*, 5770–5779, doi:10.3168/jds.2018-14429.
160. Cai, X.; Ma, F.; Jiang, J.; Yang, X.; Zhang, Z.; Jian, Z.; Liang, M.; Li, P.; Yu, L. Fe-N-C Single-Atom Nanozyme for Ultrasensitive, on-Site and Multiplex Detection of Mycotoxins Using Lateral Flow Immunoassay. *J. Hazard. Mater.* **2023**, *441*, doi:10.1016/j.jhazmat.2022.129853.
161. Yang, D.; Lei, L.; Yang, K.; Gao, K.; Jia, T.; Wang, L.; Wang, X.; Xue, C. An Immunochromatography Strip with Peroxidase-Mimicking Ferric Oxyhydroxide Nanorods-Mediated Signal Amplification and Readout. *Microchim. Acta* **2022**, *189*, doi:10.1007/s00604-021-05085-5.
162. Panferov, V.G.; Safenkova, I. V.; Zherdev, A. V.; Dzantiev, B.B. Urchin Peroxidase-Mimicking Au@Pt Nanoparticles as a Label in Lateral Flow Immunoassay: Impact of Nanoparticle Composition on Detection Limit of Clavibacter Michiganensis. *Microchim. Acta* **2020**, *187*, doi:10.1007/s00604-020-04253-3.
163. Panferov, V.G.; Byzova, N.A.; Zherdev, A. V; Dzantiev, B.B. Peroxidase-Mimicking Nanozyme with Surface-Dispersed Pt Atoms for the Colorimetric Lateral Flow Immunoassay of C-Reactive Protein. *Microchim. Acta* **2021**, *188*, 309, doi:10.1007/s00604-021-04968-x.
164. Zhang, J.; Yu, Q.; Qiu, W.; Li, K.; Qian, L.; Zhang, X.; Liu, G. Gold-Platinum Nanoflowers as a Label and as an Enzyme Mimic for Use in Highly Sensitive Lateral Flow Immunoassays: Application to Detection of Rabbit IgG. *Microchim. Acta* **2019**, *186*, 357, doi:10.1007/s00604-019-3464-z.
165. Gao, Z.; Ye, H.; Tang, D.; Tao, J.; Habibi, S.; Minerick, A.; Tang, D.; Xia, X. Platinum-Decorated Gold Nanoparticles with Dual Functionalities for Ultrasensitive Colorimetric in Vitro Diagnostics. *Nano Lett.* **2017**, *17*, 5572–5579, doi:10.1021/acs.nanolett.7b02385.
166. Lyu, Z.; Ding, S.; Tieu, P.; Fang, L.; Li, X.; Li, T.; Pan, X.; Engelhard, M.H.; Ruan, X.; Du, D.; et al. Single-Atomic Site Catalyst Enhanced Lateral Flow Immunoassay for Point-of-Care Detection of Herbicide. *Research* **2022**, *2022*, 9823290, doi:10.34133/2022/9823290.
167. Cai, X.; Luo, Y.; Zhu, C.; Huang, D.; Song, Y. Rhodium Nanocatalyst-Based Lateral Flow Immunoassay for Sensitive Detection of Staphylococcal Enterotoxin B. *Sensors Actuators B Chem.* **2022**, *367*, 132066, doi:10.1016/j.snb.2022.132066.
168. Tian, M.; Xie, W.; Zhang, T.; Liu, Y.; Lu, Z.; Li, C.M.; Liu, Y. A Sensitive Lateral Flow Immunochromatographic Strip with Prussian Blue Nanoparticles Mediated Signal Generation and Cascade Amplification. *Sensors Actuators, B Chem.* **2020**, *309*, 127728, doi:10.1016/j.snb.2020.127728.
169. Panferov, V.G.; Safenkova, I. V.; Zherdev, A. V.; Dzantiev, B.B. The Steadfast Au@Pt Soldier: Peroxide-Tolerant Nanozyme for Signal Enhancement in Lateral Flow Immunoassay of Peroxidase-Containing Samples. *Talanta* **2021**, *225*, 121961, doi:10.1016/j.talanta.2020.121961.
170. Xu, D.; An, X.; Wang, Y.; Qian, L.; Qiu, W.; Zhang, X.; Liu, G. Ultrasensitive Lateral Flow Biosensor Based on PtAu@CNTs Nanocomposite Catalytic Chromogenic Signal Amplification Strategy for the Detection of Nucleic Acid. *Anal. Chim. Acta* **2023**, *1260*, 341205, doi:10.1016/j.aca.2023.341205.

171. Yao, N.; Li, X.; Tian, Y.; Huang, Z.; Duan, Y. Core-Shell Au@PdNPs Based Colorimetric Enhanced Lateral Flow Immunoassay for C-Reactive Protein Detection. *Sensors Actuators B Chem.* **2023**, *379*, 133247, doi:10.1016/j.snb.2022.133247.
172. Zhang, J.; Tang, L.; Yu, Q.; Qiu, W.; Li, K.; Cheng, L.; Zhang, T.; Qian, L.; Zhang, X.; Liu, G. Gold-Platinum Nanoflowers as Colored and Catalytic Labels for Ultrasensitive Lateral Flow MicroRNA-21 Assay. *Sensors Actuators, B Chem.* **2021**, *344*, 130325, doi:10.1016/j.snb.2021.130325.
173. Meng, X.; Zou, S.; Li, D.; He, J.; Fang, L.; Wang, H.; Yan, X.; Duan, D.; Gao, L. Nanozyme-Strip for Rapid and Ultrasensitive Nucleic Acid Detection of SARS-CoV-2. *Biosens. Bioelectron.* **2022**, *217*, 114739, doi:10.1016/j.bios.2022.114739.
174. Yin, X.; Hou, J.; Guo, J.; Li, J.; Yang, D.; Luo, L.; Dou, L.; Liang, Y.; Wang, Y.; Wang, J.; et al. Nanocatalyst-Triggered Cascade Immunoassay: Multi-Model Immunochromatography Assay for Sensitive Detection of Salmonella Typhimurium. *Chem. Eng. J.* **2023**, *469*, 143979, doi:10.1016/j.cej.2023.143979.
175. Huang, X.; Chen, L.; Zhi, W.; Zeng, R.; Ji, G.; Cai, H.; Xu, J.; Wang, J.; Chen, S.; Tang, Y.; et al. Urchin-Shaped Au–Ag@Pt Sensor Integrated Lateral Flow Immunoassay for Multimodal Detection and Specific Discrimination of Clinical Multiple Bacterial Infections. *Anal. Chem.* **2023**, doi:10.1021/acs.analchem.3c01631.
176. Liu, Y.; Zhang, Z.; Yu, J.; Xie, J.; Li, C.M. A Concentration−dependent Multicolor Conversion Strategy for Ultrasensitive Colorimetric Immunoassay with the Naked Eye. *Anal. Chim. Acta* **2017**, *963*, 129–135, doi:10.1016/j.aca.2017.01.034.
177. Liu, Y.; Xie, J.; Zhang, Z.; Lu, Z. An Ultrasensitive Colorimetric Strategy for Protein: O -GlcNAcylation Detection via Copper Deposition-Enabled Nonenzymatic Signal Amplification. *RSC Adv.* **2016**, *6*, 89484–89491, doi:10.1039/c6ra17119b.
178. Phan, L.M.T.; Cho, S. A Multi-Chamber Paper-Based Platform for the Detection of Amyloid β Oligomers 42 via Copper-Enhanced Gold Immunoblotting. *Biomolecules* **2021**, *11*, doi:10.3390/biom11070948.
179. Kim, J.; Park, J.; Lin, M.; Kim, S.; Kim, G.; Park, S.; Ko, G.; Nam, J. Sensitive, Quantitative Naked-Eye Biodetection with Polyhedral Cu Nanoshells. *Adv. Mater.* **2017**, *29*, 1702945, doi:10.1002/adma.201702945.
180. Zhou, G.; Bergeron, S.; Juncker, D. High-Performance Low-Cost Antibody Microarrays Using Enzyme-Mediated Silver Amplification. *J. Proteome Res.* **2015**, *14*, 1872–1879, doi:10.1021/pr501259e.
181. Lathwal, S.; Sikes, H.D. Assessment of Colorimetric Amplification Methods in a Paper-Based Immunoassay for Diagnosis of Malaria. *Lab Chip* **2016**, *16*, 1374–1382, doi:10.1039/c6lc00058d.
182. Lan, M.; Guo, Y.; Zhao, Y.; Liu, Y.; Gui, W.; Zhu, G. Multi-Residue Detection of Pesticides Using a Sensitive Immunochip Assay Based on Nanogold Enhancement. *Anal. Chim. Acta* **2016**, *938*, 146–155, doi:10.1016/j.aca.2016.07.044.
183. Li, B.S.; Zhao, L.F.; Zhang, C.; Hei, X.H.; Li, F.; Li, X.B.; Shen, J.; Li, Y.Y.; Huang, Q.; Xu, S.Q. Ultra-Sensitive Colorimetric Method to Quantitate Hundreds of Polynucleotide Molecules by Gold Nanoparticles with Silver Enhancement. *Anal. Sci.* **2006**, *22*, 1367–1370, doi:10.2116/analsci.22.1367.
184. Gupta, S.; Huda, S.; Kilpatrick, P.K.; Velev, O.D. Characterization and Optimization of Gold Nanoparticle-Based Silver-Enhanced Immunoassays. *Anal. Chem.* **2007**, *79*, 3810–3820, doi:10.1021/ac062341m.
185. Kim, D.; Daniel, W.L.; Mirkin, C.A. Microarray-Based Multiplexed Scanometric Immunoassay for Protein Cancer Markers Using Gold Nanoparticle Probes. *Anal. Chem.* **2009**, *81*, 9183–9187, doi:10.1021/ac9018389.
186. Ma, Z.; Sui, S.F. Naked-Eye Sensitive Detection of Immunoglubulin G by Enlargement of Au Nanoparticles in Vitro. *Angew. Chemie - Int. Ed.* **2002**, *41*, 2176–2179, doi:10.1002/1521-3773(20020617)41.
187. Shin, J.H.; Hong, J.; Go, H.; Park, J.; Kong, M.; Ryu, S.; Kim, K.-P.; Roh, E.; Park, J.-K. Multiplexed Detection of Foodborne Pathogens from Contaminated Lettuces Using a Handheld Multistep Lateral Flow Assay Device. *J. Agric. Food Chem.* **2018**, *66*, 290–297, doi:10.1021/acs.jafc.7b03582.
188. Watanabe, H.; Satake, A.; Kido, Y.; Tsuji, A. Monoclonal-Based Enzyme-Linked Immunosorbent Assay and Immunochromatographic Rapid Assay for Dihydrostreptomycin in Milk. *Anal. Chim. Acta* **2002**, *472*, 45–53, doi:10.1016/S0003-2670(02)00983-2.
189. Cho, J.-H.; Han, S.; Paek, E.-H.; Cho, I.; Paek, S. Plastic ELISA-on-a-Chip Based on Sequential Cross-Flow Chromatography. *Anal. Chem.* **2006**, *78*, 793–800, doi:10.1021/ac051453v.
190. Kawde, A.; Mao, X.; Xu, H.; Zeng, Q.; He, Y.; Liu, G. Moving Enzyme-Linked ImmunoSorbent Assay to the Point-of-Care Dry-Reagent Strip Biosensors. *Am. J. Biomed. Sci.* **2010**, *2*, 23–32, doi:10.5099/aj100100023.
191. Du, D.; Wang, J.; Wang, L.; Lu, D.; Lin, Y. Integrated Lateral Flow Test Strip with Electrochemical Sensor for Quantification of Phosphorylated Cholinesterase: Biomarker of Exposure to Organophosphorus Agents. *Anal. Chem.* **2012**, *84*, 1380–1385, doi:10.1021/ac202391w.
192. Wang, Y.; Fill, C.; Nugen, S.R. Development of Chemiluminescent Lateral Flow Assay for the Detection of Nucleic Acids. *Biosensors* **2012**, *2*, 32–42, doi:10.3390/bios2010032.
193. Safronova, V. a.; Samsonova, J. V.; Grigorenko, V.G.; Osipov, a. P. Lateral Flow Immunoassay for Progesterone Detection. *Moscow Univ. Chem. Bull.* **2012**, *67*, 241–248, doi:10.3103/S0027131412050045.
194. Cho, I.-H.; Irudayaraj, J. Lateral-Flow Enzyme Immunoconcentration for Rapid Detection of Listeria Monocytogenes. *Anal. Bioanal. Chem.* **2013**, *405*, 3313–3319, doi:10.1007/s00216-013-6742-3.
195. Joung, H.A.; Oh, Y.K.; Kim, M.G. An Automatic Enzyme Immunoassay Based on a Chemiluminescent Lateral Flow Immunosensor. *Biosens. Bioelectron.* **2014**, *53*, 330–335, doi:10.1016/j.bios.2013.10.004.
196. Zangheri, M.; Cevenini, L.; Anfossi, L.; Baggiani, C.; Simoni, P.; Di Nardo, F.; Roda, A. A Simple and Compact Smartphone Accessory for Quantitative Chemiluminescence-Based Lateral Flow Immunoassay for Salivary Cortisol Detection. *Biosens. Bioelectron.* **2015**, *64*, 63–68, doi:10.1016/j.bios.2014.08.048.
197. Samsonova, J. V.; Safronova, V.A.; Osipov, A.P. Pretreatment-Free Lateral Flow Enzyme Immunoassay for Progesterone Detection in Whole Cows' Milk. *Talanta* **2015**, *132*, 685–689, doi:10.1016/j.talanta.2014.10.043.

198. Zangheri, M.; Di Nardo, F.; Anfossi, L.; Giovannoli, C.; Baggiani, C.; Roda, A.; Mirasoli, M. A Multiplex Chemiluminescent Biosensor for Type B-Fumonisins and Aflatoxin B1 Quantitative Detection in Maize Flour. *Analyst* **2015**, *140*, 358–365, doi:10.1039/c4an01613k.
199. Lim, G.-S.; Seo, S.-M.; Paek, S.-H.; Kim, S.-W.; Jeon, J.-W.; Kim, D.-H.; Cho, I.-H.; Paek, S.-H. Chemiluminometric Immunosensor for High-Sensitivity Cardiac Troponin I Employing a Polymerized Enzyme Conjugate as a Tracer. *Sci. Rep.* **2015**, *5*, 14848, doi:10.1038/srep14848.
200. Ren, W.; Cho, I.-H.; Zhou, Z.; Irudayaraj, J. Ultrasensitive Detection of Microbial Cells Using Magnetic Focus Enhanced Lateral Flow Sensors. *Chem. Commun.* **2016**, *52*, 4930–4933, doi:10.1039/C5CC10240E.
201. Shin, J.H.; Park, J.K. Functional Packaging of Lateral Flow Strip Allows Simple Delivery of Multiple Reagents for Multistep Assays. *Anal. Chem.* **2016**, *88*, 10374–10378, doi:10.1021/acs.analchem.6b02869.
202. Zangheri, M.; Di Nardo, F.; Mirasoli, M.; Anfossi, L.; Nascetti, A.; Caputo, D.; De Cesare, G.; Guardigli, M.; Baggiani, C.; Roda, A. Chemiluminescence Lateral Flow Immunoassay Cartridge with Integrated Amorphous Silicon Photosensors Array for Human Serum Albumin Detection in Urine Samples. *Anal. Bioanal. Chem.* **2016**, *408*, 8869–8879, doi:10.1007/s00216-016-9991-0.
203. Santos, G.P. dos; Corrêa, C.C.; Kubota, L.T. A Simple, Sensitive and Reduced Cost Paper-Based Device with Low Quantity of Chemicals for the Early Diagnosis of Plasmodium Falciparum Malaria Using an Enzyme-Based Colorimetric Assay. *Sensors Actuators, B Chem.* **2018**, *255*, 2113–2120, doi:10.1016/j.snb.2017.09.005.
204. Zangheri, M.; Mirasoli, M.; Guardigli, M.; Di Nardo, F.; Anfossi, L.; Baggiani, C.; Simoni, P.; Benassai, M.; Roda, A. Chemiluminescence-Based Biosensor for Monitoring Astronauts' Health Status during Space Missions: Results from the International Space Station. *Biosens. Bioelectron.* **2019**, *129*, 260–268, doi:10.1016/j.bios.2018.09.059.
205. Kim, H.T.; Jin, E.; Lee, M.H. Portable Chemiluminescence-Based Lateral Flow Assay Platform for the Detection of Cortisol in Human Serum. *Biosensors* **2021**, *11*, doi:10.3390/bios11060191.
206. Zangheri, M.; Di Nardo, F.; Calabria, D.; Marchegiani, E.; Anfossi, L.; Guardigli, M.; Mirasoli, M.; Baggiani, C.; Roda, A. Smartphone Biosensor for Point-of-Need Chemiluminescence Detection of Ochratoxin A in Wine and Coffee. *Anal. Chim. Acta* **2021**, *1163*, 338515, doi:10.1016/j.aca.2021.338515.
207. Lin, D.; Li, B.; Fu, L.; Qi, J.; Xia, C.; Zhang, Y.; Chen, J.; Choo, J.; Chen, L. A Novel Polymer-Based Nitrocellulose Platform for Implementing a Multiplexed Microfluidic Paper-Based Enzyme-Linked Immunosorbent Assay. *Microsystems Nanoeng.* **2022**, *8*, doi:10.1038/s41378-022-00385-z.
208. Panraksa, Y.; Jang, I.; Carrell, C.S.; Amin, A.G.; Chailapakul, O.; Chatterjee, D.; Henry, C.S. Simple Manipulation of Enzyme-Linked Immunosorbent Assay (ELISA) Using an Automated Microfluidic Interface. *Anal. Methods* **2022**, 1774–1781, doi:10.1039/d2ay00326k.
209. Tahmasebi, M.; Bamdad, T.; Svendsen, W.E.; Forouzandeh-Moghadam, M. An Enzymatic Nucleic Acid Vertical Flow Assay. *Anal. Bioanal. Chem.* **2022**, *414*, 3605–3615, doi:10.1007/s00216-022-03988-7.
210. Jiang, T.; Song, Y.; Wei, T.; Li, H.; Du, D.; Zhu, M.J.; Lin, Y. Sensitive Detection of Escherichia Coli O157:H7 Using Pt-Au Bimetal Nanoparticles with Peroxidase-like Amplification. *Biosens. Bioelectron.* **2016**, *77*, 687–694, doi:10.1016/j.bios.2015.10.017.
211. Zhang, L.; Chen, Y.; Cheng, N.; Xu, Y.; Huang, K.; Luo, Y.; Wang, P.; Duan, D.; Xu, W. Ultrasensitive Detection of Viable Enterobacter Sakazakii by a Continual Cascade Nanozyme Biosensor. *Anal. Chem.* **2017**, *89*, 10194–10200, doi:10.1021/acs.analchem.7b01266.
212. Zhao, Y.; Yang, M.; Fu, Q.; Ouyang, H.; Wen, W.; Song, Y.; Zhu, C.; Lin, Y.; Du, D. A Nanozyme- and Ambient Light-Based Smartphone Platform for Simultaneous Detection of Dual Biomarkers from Exposure to Organophosphorus Pesticides. *Anal. Chem.* **2018**, *90*, 7391–7398, doi:10.1021/acs.analchem.8b00837.
213. Lin, B.; Guan, Z.; Song, Y.; Song, E.; Lu, Z.; Liu, D.; An, Y.; Zhu, Z.; Zhou, L.; Yang, C. Lateral Flow Assay with Pressure Meter Readout for Rapid Point-of-Care Detection of Disease-Associated Protein. *Lab Chip* **2018**, *18*, 965–970, doi:10.1039/c8lc00010g.
214. Huang, D.; Lin, B.; Song, Y.; Guan, Z.; Cheng, J.; Zhu, Z.; Yang, C. Staining Traditional Colloidal Gold Test Strips with Pt Nanoshell Enables Quantitative Point-of-Care Testing with Simple and Portable Pressure Meter Readout. *ACS Appl. Mater. Interfaces* **2019**, *11*, 1800–1806, doi:10.1021/acsami.8b15562.
215. Lin, S.; Zheng, D.; Li, A.; Chi, Y. Black Oxidized 3,3′,5,5′-Tetramethylbenzidine Nanowires (OxTMB NWs) for Enhancing Pt Nanoparticle-Based Strip Immunosensing. *Anal. Bioanal. Chem.* **2019**, *411*, 4063–4071, doi:10.1007/s00216-019-01745-x.
216. Tripathi, P.; Kumar, A.; Sachan, M.; Gupta, S.; Nara, S. Aptamer-Gold Nanozyme Based Competitive Lateral Flow Assay for Rapid Detection of CA125 in Human Serum. *Biosens. Bioelectron.* **2020**, *165*, 112368, doi:10.1016/j.bios.2020.112368.
217. Wu, S.C.; Tsai, T.T.; Li, T.H.; Tung, C.Y.; Chiu, P.Y.; Lin, J.H.; Chen, C.F. Palladium-Platinum Bimetallic Nanomaterials and Their Application in Staphylococcus Aureus Detection on Paper-Based Devices. *Biosens. Bioelectron.* **2022**, *216*, 114669, doi:10.1016/j.bios.2022.114669.
218. Chen, Y.; Ren, J.; Yin, X.; Li, Y.; Shu, R.; Wang, J.; Zhang, D. Vanadium Disulfide Nanosheet Boosts Optical Signal Brightness as a Superior Enzyme Label to Improve the Sensitivity of Lateral Flow Immunoassay. *Anal. Chem.* **2022**, *94*, 8693–8703, doi:10.1021/acs.analchem.2c01008.
219. Kong, D.Y.; Heo, N.S.; Kang, J.W.; Lee, J.B.; Kim, H.J.; Kim, M. Il Nanoceria-Based Lateral Flow Immunoassay for Hydrogen Peroxide-Free Colorimetric Biosensing for C-Reactive Protein. *Anal. Bioanal. Chem.* **2022**, *414*, 3257–3265, doi:10.1007/s00216-022-03877-z.
220. Li, N.; Xi, X.; Zhu, J.; Wu, X.; Zhang, X.; Wang, S.; Wen, W. High Sensitivity and Rapid Detection of Hepatitis B Virus DNA Using Lateral Flow Biosensors Based on Au@Pt Nanorods in the Absence of Hydrogen Peroxide. *Analyst* **2022**, *147*, 423–429, doi:10.1039/D1AN02084F.

221. Luo, Y.; Luo, H.; Zou, S.; Jiang, J.; Duan, D.; Chen, L.; Gao, L. An In Situ Study on Nanozyme Performance to Optimize Nanozyme-Strip for Aβ Detection. *Sensors* **2023**, *23*, 3414, doi:10.3390/s23073414.
222. Liang, J.; Liu, Z.; Fang, Y.; Shen, X.; Xu, Z.; Lei, H.; Huang, X.; Li, X. Two Kinds of Lateral Flow Immunoassays Based on Multi-functional Magnetic Prussian Blue Nanoenzyme and Colloidal Gold for the Detection of 38 β-Agonists in Swine Urine and Pork. *Food Chem.* **2023**, *417*, 135897, doi:10.1016/j.foodchem.2023.135897.
